# Integrated Experimental and Computational Investigation of Salicylaldehyde-Derived Imine/Amine Derivatives as Antioxidant and Cytotoxic Agents

**DOI:** 10.3390/ma19183980

**Published:** 2026-09-19

**Authors:** Rania Boukerzaza, Chamseddine Derabli, Imene Amine Khodja, Abdellatif Ben Kaida, Jose F. Costa-Rubio, Chawki Bensouici, Stephanie Hesse, Horacio Pérez-Sánchez, Houssem Boulebd

**Affiliations:** 1 Biotechnologies Laboratory, Higher National School of Biotechnology Taoufik Khaznadar, Nouveau Pôle Universitaire Ali Mendjeli, Constantine 25100, Algeria; raniaboukerzaza@gmail.com (R.B.); cderabli@yahoo.fr (C.D.); abdellatifbenkaida@gmail.com (A.B.K.); 2 Laboratory of Synthesis of Molecules with Biological Interest, Frères Mentouri Constantine 1 University, Constantine 25017, Algeria; aminekhodja.imene@yahoo.fr; 3 Structural Bioinformatics and High Performance Computing Research Group (BIO-HPC), Universidad Católica de Murcia (UCAM), 30107 Murcia, Spain; jfcosta8@ucam.edu; 4 Biotechnology Research Center, Constantine 25000, Algeria; chawkiislam@yahoo.fr; 5 LCP-A2MC, Université de Lorraine, 5700 Metz, France; stephanie.hesse@univ-lorraine.fr

**Keywords:** Schiff bases, phenolic antioxidants, density functional theory, antioxidant mechanism, MDA-MB-231, molecular modeling

## Abstract

Imines, commonly known as Schiff bases, are widely investigated compounds because of their structural versatility and biological properties. In this study, we aimed to evaluate the antioxidant potential of salicylaldehyde-derived imines and to determine how their reduction to the corresponding secondary amines affects their radical-scavenging activity and mechanism of action. Two series of phenolic derivatives, imines I1–I6 and amines A1–A6, were synthesized and assessed using an integrated experimental and theoretical strategy combining five in vitro antioxidant assays with density functional theory calculations. Antioxidant evaluation by DPPH, ABTS, FRAP, phenanthroline, and CUPRAC assays revealed distinct activity profiles for the two series. The imine derivatives were more effective in electron-transfer-based assays, whereas the reduced amines showed stronger DPPH radical-scavenging activity. Compound A6 exhibited the highest overall antioxidant activity, with IC_50_ values (e.g., DPPH IC_50_ = 26.7 ± 0.97 µM; ABTS IC_50_ = 11.69 ± 0.43 µM) lower than BHT (DPPH IC_50_ = 85.85 ± 3.69 µM) and BHA (DPPH IC_50_ = 59.39 ± 1.9 µM) in most assays. DFT calculations in aqueous medium indicated that the phenolic OH groups govern antioxidant reactivity and identified SPLET as the dominant mechanism for A6, supported by a remarkably high rate constant (*k* = 1.40 × 10^5^ M^−1^s^−1^) and a 100% branching ratio (G), completely outcompeting the HAT mechanism (*k* = 2.28 × 10^−2^ M^−1^s^−1^). In addition to the antioxidant investigation, cytotoxicity was assessed in MDA-MB-231 breast cancer cells and Vero normal cells, revealing that the chlorinated derivatives such as I5 combine moderate anticancer activity (MDA-MB-231 IC_50_ = 122.6 ± 2.23 µM) with lower toxicity toward non-tumor cells (Vero IC_50_ > 400 µM). Molecular docking studies were conducted to elucidate the binding modes of the active derivatives within the active sites of EGFR, Tubulin, and Topoisomerase IIβ, while subsequent 100 ns molecular dynamics simulations validated the structural stability and sustained interaction energies of these predicted complexes.

## 1. Introduction

Oxidative stress arises from an imbalance between the generation of reactive oxygen species (ROS) and endogenous antioxidant defenses, resulting in oxidative damage to lipids, proteins, and DNA [1,2]. ROS such as superoxide anion (O_2_^−^), hydrogen peroxide (H_2_O_2_), hydroxyl radical (^•^OH), and peroxynitrite (ONOO^−^) are produced during mitochondrial metabolism and through NADPH oxidase activity, acting as signaling mediators at low levels but promoting pathological effects when excessively accumulated [1,3]. This redox imbalance has been associated with aging and chronic diseases, including neurodegenerative disorders, cardiovascular complications, diabetes, inflammation, and cancer, mainly through mechanisms involving genomic instability and tissue injury [4,5]. Endogenous protective systems such as superoxide dismutase, catalase, glutathione peroxidase, and glutathione provide basal antioxidant capacity, whereas exogenous antioxidants from dietary sources or synthetic preparation offer additional support under conditions of oxidative overload [1,6]. Exogenous antioxidants can neutralize radicals, chelate redox-active metals, and enhance antioxidant enzyme expression to interrupt ROS-driven chain processes, thereby contributing to redox homeostasis [7,8,9].

In cancer, oxidative stress is described as a dual-function factor, where moderate ROS levels promote tumorigenesis through pathways such as NF-κB, MAPK, and PI3K/Akt, leading to enhanced proliferation, angiogenesis, and metastasis, whereas elevated ROS levels trigger apoptosis [9]. Cancer cells were characterized by increased ROS production arising from metabolic rewiring and mitochondrial dysfunction, together with reduced antioxidant capacity (e.g., decreased SOD and thioredoxin), which contributed to mutagenesis, therapy resistance, and disease progression [10,11]. Accordingly, antioxidant-based strategies were highlighted as potential interventions to restore redox balance, suppress tumor growth, and enhance sensitivity to anticancer treatments [9].

Schiff bases (imines, R_1_R_2_C=NR_3_) have emerged as versatile scaffolds due to their straightforward one-step synthesis via amine–carbonyl condensation, providing high yields, favorable atom economy, and convenient structural diversification [12]. Recent Schiff base derivatives were reported to exhibit strong antioxidant performance in DPPH and FRAP assays and effective metal-chelating ability, comparable to reference antioxidants such as ascorbic acid and BHT, while also showing selective cytotoxicity against breast, colon, lung, and prostate cancer cell lines through ROS modulation and apoptosis induction [13,14]. Representative classes such as indolyl imines, quinone imines, and thiophene-imines were described to display combined antioxidant–anticancer profiles, supporting their use as efficient frameworks for targeting oxidative stress-driven oncogenesis [15].

In the present study, the synthesis of a series of phenolic imine derivatives was carried out, and their antioxidant and anticancer activities were evaluated. To examine the influence of the imine functionality on the investigated biological properties, an additional reduction step was performed to convert the imine group into the corresponding secondary amine, affording a second series of structurally related amine analogs. The antioxidant activity of all prepared compounds was assessed using five complementary in vitro assays (DPPH, ABTS, FRAP, phenanthroline, and CUPRAC), enabling evaluation of both radical-scavenging and metal-reducing behaviors. In parallel, antiproliferative activity was determined by the MTT assay against MDA-MB-231 triple-negative breast cancer cells, while Vero cells were used as a noncancerous control to provide a preliminary estimate of selectivity. Furthermore, molecular docking studies and subsequent 100 ns molecular dynamics simulations were conducted for the most promising derivative (I5) against key cancer-relevant targets (EGFR, Tubulin, and Topoisomerase II) in order to rationalize the observed cytotoxic activity and confirm binding stability. Overall, this integrated experimental and computational strategy enabled meaningful structure–activity relationships to be established between substitution patterns, redox behavior, and selective anticancer effects, highlighting phenolic-derived imine/amine scaffolds as promising leads for further optimization.

## 2. Materials and Methods

### 2.1. General Information

Thin-layer chromatography (TLC) was carried out on silica gel 60 F_254_ plates (0.2 mm thickness; Merck, Darmstadt, Germany) to confirm the purity of the synthesized compounds. Melting points were determined using a Köfler hot-stage apparatus (Wagner & Munz GmbH, Munich, Germany, and infrared (IR) spectra were recorded using an FTIR spectrometer (Shimadzu Corporation, IRAffinity-1, Kyoto, Japan), both performed at the National School of Biotechnology, Constantine. ^1^H and ^13^C nuclear magnetic resonance (NMR) spectra were recorded at 400 MHz at the LCP-A2MC Laboratory, University of Lorraine. Peak multiplicities were assigned as singlet (s), doublet (d), doublet of doublets (dd), triplet (t), triplet of doublets (td), and multiplet (m).

### 2.2. Synthesis

#### 2.2.1. Synthesis of Imines I1–I6

The synthesis of the Schiff bases (I1–I6) was carried out following the procedure reported by [16]. Equimolar quantities (1 mmol) of salicylaldehyde, 5-bromo-salicylaldehyde, or 5-chloro-salicylaldehyde were added to a stirred ethanolic solution (5 mL) containing 3- or 4-aminophenol. The reaction mixture was maintained at room temperature for 10 min under continuous stirring. After cooling in an ice bath, the formed precipitate was filtered through a Büchner funnel, washed with a small amount of cold ethanol, and dried under vacuum.

4-((2-hydroxybenzylidene) amino) phenol (Oc2ccc(/N=C/c1ccccc1O)cc2) (I1)

Compound I1 was obtained as a yellow solid (76%). M.p: 136 °C; FTIR (νmax, cm^−1^): 1618 (C=N);

^1^H NMR (400 MHz, CDCl_3_) δ (ppm): 8.60 (s, 1H, CH), 7.39–7.34 (m, 2H), 7.24–7.20 (m, 2H), 7.02 (d, J = 8 Hz, 1H), 6.96–688 (m, 3H), ^13^C NMR (100 MHz, CDCl_3_): δ (ppm):161.1, 160.6, 155.2, 141.4, 132.9, 132.1, 122.6, 119.4, 119.2, 117.3, 116.3.

3-((2-hydroxybenzylidene) amino) phenol (Oc2cccc(/N=C/c1ccccc1O)c2) (I2)

Compound I2 was obtained as an orange solid (78%). M.p: 122 °C; FTIR (νmax, cm^−1^): 1591 (C=N);

^1^H NMR (400 MHz, CDCl_3_) δ (ppm): 13.14 (s, 1H, OH), 9.66 (s, 1H, OH), 8.90 (s, 1H, CH), 7.65 (d, J = 8 Hz, 1H), 7.43–7.38 (m, 1H), 7.24 (t, J = 8 Hz, 1H), 6.97 (t, J = 8 Hz, 2H), 6.84–6.73 (m, 3H), ^13^C NMR (100 MHz, CDCl_3_): δ (ppm): 163.7, 160.8, 158.8, 149.7, 133.7, 133.1, 130.7, 119.7, 119.6, 117.0, 114.6, 112.5, 108.6.

4-((5-Bromo-2-hydroxybenzylidene) amino) phenol (Oc2ccc(/N=C/c1cc(Br)ccc1O)cc2) (I3)

Compound I3 was obtained as a yellow solid (80%). M.p: 232 °C; FTIR (νmax, cm^−1^): 1612 (C=N);

^1^H NMR (400 MHz, CDCl_3_) δ (ppm): 13.45 (s, 1H, OH), 9.75 (s, 1H, OH), 8.88 (s, 1H, CH), 7.80 (d, J = 8 Hz, 1H), 7.50 (dd, J = 8 Hz, 2 Hz, 1H), 7.31 (dd, J = 8 Hz, 2 Hz, 2H), 6.91(d, J = 8 Hz, 1H), 6.85 (dd, J = 8 Hz, 2 Hz, 2H); ^13^C NMR (100 MHz, CDCl_3_): δ (ppm): 159.7, 159.1, 157.8, 139.3, 135.2, 134.2, 123.3, 121.8, 119.4, 116.5, 110.2.

3-((5-Bromo-2-hydroxybenzylidene) amino) phenol (Oc2cccc(/N=C/c1cc(Br)ccc1O)c2) (I4)

Compound I4 was obtained as an orange solid (83%). M.p: 170 °C; FTIR (νmax, cm^−1^): 1638 (C=N); ^1^H NMR (400 MHz, CDCl_3_) δ (ppm): 13.13 (s, 1H, OH), 9.69 (s, 1H, OH), 8.88 (s, 1H, CH), 7.86 (s, 1H), 7.54 (t, J = 8 Hz, 1H), 7.25 (t, J = 8 Hz, 1H), 6.95(t, J = 8 Hz, 1H), 6.83–6.73 (m, 3H); ^13^C NMR (100 MHz, CDCl_3_): δ (ppm):162.1, 159.9, 158.8, 149.5, 135.9, 134.5, 130.7, 121.6, 119.5, 114.9, 112.6, 110.4, 108.6.

4-((5-Chloro-2-hydroxybenzylidene) amino) phenol (Oc2ccc(/N=C/c1cc(Cl)ccc1O)cc2) (I5)

Compound I5 was obtained as a yellow solid (88%). M.p: 238 °C; FTIR (νmax, cm^−1^): 1618 (C=N);

^1^H NMR (400 MHz, CDCl_3_) δ (ppm): 13.41 (s, 1H, OH), 9.75 (s, 1H, OH), 8.88 (s, 1H, CH), 7.67 (s, 1H), 7.38 (d, J = 8 Hz, 1H),7.32 (t, J = 8 Hz, 2H), 6.96 (d, J = 8 Hz, 1H), 6.85 (t, J = 8 Hz, 2H); ^13^C NMR (100 MHz, CDCl_3_): δ (ppm):159.3, 159.2, 157.8, 139.3, 132.4, 131.3, 123.3, 122.8, 121.2, 118.9, 116.5.

3-((5-Chloro-2-hydroxybenzylidene) amino) phenol (Oc2cccc(/N=C/c1cc(Cl)ccc1O)c2) (I6)

Compound I6 was obtained as an orange solid (90%). M.p: 170 °C; FTIR (νmax, cm^−1^): 1638 (C=N);

^1^H NMR (400 MHz, CDCl_3_) δ (ppm): 13.10 (s, 1H, OH), 9.69 (s, 1H, OH), 8.88 (s, 1H, CH), 7.74 (s, 1H), 7.43 (t, J = 8 Hz, 1H), 7.25 (t, J = 8 Hz, 1H), 7.00 (t, J = 8 Hz, 1H), 6.83–6.73 (m, 3H); ^13^C NMR (100 MHz, CDCl_3_): δ (ppm):162.2, 159.6, 158.8, 149.5, 133.2, 131.5, 130.7, 123.0, 121.0, 119.1, 114.9, 112.6, 108.6.

#### 2.2.2. Synthesis of Secondary Amines A1–A6

The secondary amines (A1–A6) were synthesized by reducing the previously obtained imines (I1–I6) according to the method described by [16]. Each imine (0.7 mmol) was dissolved in 5 mL of absolute ethanol and cooled to approximately 5 °C. Sodium borohydride (0.4 mmol, corresponding to 0.57 equiv. relative to the imine) was added gradually under stirring, ensuring that the temperature did not exceed 10 °C. After 5 min of reaction, the mixture was allowed to stand. The resulting product was filtered, washed with cold ethanol, and dried under vacuum.

4-((2-hydroxybenzyl) amino)phenol (Oc2ccc(NCc1ccccc1O)cc2) (A1)

Compound A1 was obtained as a white solid (78%). M.p: 116 °C; FTIR (νmax, cm^−1^): 3447 (NH);

1H NMR (400 MHz, DMSO-d_6_) δ (ppm): 9.40 (s, 1H, OH), 8.44 (s, 1H, OH), 7.19 (dd, J = 8 Hz, 2 Hz, 1H), 7.03 (td, J = 8 Hz, 2 Hz, 1H), 6.80 (d, J = 8 Hz, 1H), 6.72 (t, J = 8 Hz, 1H), 6.54–6.51 (m, 2H), 6.47–6.44 (m, 2H), 5.31 (s, 1H, NH), 4.11 (s, 2H, CH2); 13C NMR (100 MHz, DMSO-d_6_): δ (ppm): 155.1, 148.4, 141.8, 128.3, 127.3, 126.1, 118.7, 115.6, 114.8, 113.7, 42.7.

3-((2-hydroxybenzyl) amino) phenol (Oc2cccc(NCc1ccccc1O)c2) (A2)

Compound A2 was obtained as a white solid (62%). M.p: 94 °C; FTIR (νmax, cm^−1^): 3418 (NH);

^1^H NMR (400 MHz, DMSO-d_6_) δ (ppm): 7.03 (d, J = 8 Hz, 1H), 6.88 (td, J = 8 Hz, 2 Hz, 1H), 6.77 (t, J = 8 Hz, 1H), 6.68 (d, J = 8 Hz, 1H), 6.41 (t, J = 8 Hz, 1H), 6.05–6.00 (m, 2H), 5.94–5.92 (m, 1H), 4.67 (s, 1H, NH), 4.10 (s, 2H, CH2); ^13^C NMR (100 MHz, DMSO-d_6_): δ (ppm): 160.8, 159.4, 150.6, 129.2, 127.8, 127.2, 126.5, 116.2, 114.4, 103.6, 103.3, 99.8, 43.1.

4-((5-Bromo-2-hydroxybenzyl) amino) phenol (Oc2ccc(NCc1cc(Br)ccc1O)cc2) (A3)

Compound A3 was obtained as an orange solid (86%). M.p: 138 °C; FTIR (νmax, cm^−1^): 3254 (NH);

^1^H NMR (400 MHz, DMSO-d_6_) δ (ppm): 7.31 (d, J = 8 Hz, 1H), 7.18 (dd, J = 8 Hz, 2 Hz, 1H), 6.76 (d, J = 8 Hz, 1H), 6.55–6.52 (m, 2H), 6.43–6.41 (m, 2H),5.47 (s, 1H, NH), 4.10 (s, 2H, CH2); ^13^C NMR (100 MHz, DMSO-d_6_): δ (ppm): 155.0, 148.5, 141.3, 130.3, 129.8, 129.4, 117.0, 115.7, 113.6, 110.0, 42.1.

3-((5-Bromo-2-hydroxybenzyl) amino) phenol (Oc2cccc(NCc1cc(Br)ccc1O)c2) (A4)

Compound A4 was obtained as a red solid (50%). M.p: 82 °C; FTIR (νmax, cm^−1^): 3447 (NH);

^1^H NMR (400 MHz, DMSO-d_6_) δ (ppm): 9.86 (s, 1H, OH), 8.90 (s, 1H, OH), 7.26 (d, J = 8 Hz, 1H), 7.19 (dd, J = 8 Hz, 2 Hz, 1H), 6.82 (t, J = 8 Hz, 1H), 6.78 (d, J = 8 Hz, 1H), 6.04–5.94 (m, 3H), 4.12 (s, 2H, CH2); ^13^C NMR (100 MHz, DMSO-d_6_): δ (ppm): 158.2, 154.2, 149.9, 130.0, 129.8, 129.5, 129.1, 116.9, 110.1, 103.8, 103.5, 99.0, 40.9.

4-((5-Chloro-2-hydroxybenzyl) amino) phenol (Cc1cc(Cl)ccc1O.CNc1ccc(O)cc1) (A5)

Compound A5 was obtained as a yellow solid (90%). M.p: 128 °C; FTIR (νmax, cm^−1^): 3447 (NH);

^1^H NMR (400 MHz, DMSO-d_6_) δ (ppm): 9.06 (s, 1H, OH), 8.89 (s, 1H, OH), 7.19 (d, J = 2 Hz, 1H), 7.06 (dd, J = 8 Hz, 2 Hz, 1H), 6.81 (d, J = 8 Hz, 1H), 6.54 (d, J = 8 Hz, 2H), 6.43 (d, J = 8 Hz, 2H), 5.46 (s, 1H, NH), 4.11 (s, 2H, CH2); ^13^C NMR (100 MHz, DMSO-d_6_): δ (ppm): 154.4, 149.0, 141.8, 129.3, 128.0, 127.3, 122.9, 116.8, 116.2, 114.3, 42.6.

3-((5-Chloro-2-hydroxybenzyl) amino) phenol (Oc2cccc(NCc1cc(Cl)ccc1O)c2) (A6)

Compound A6 was obtained as a white solid (62%). M.p: 90 °C; FTIR (νmax, cm^−1^): 3447 (NH);

^1^H NMR (400 MHz, DMSO-d_6_) δ (ppm): 9.83 (s, 1H, OH), 8.90 (s, 1H, OH), 7.12 (d, J = 2 Hz, 1H), 7.06 (d, J = 2 Hz, 1H), 6.83–6.79 (m, 2H), 6.03–5.95 (m, 4H), 4.12 (d, 2H, CH2); ^13^C NMR (100 MHz, DMSO-d_6_): δ (ppm): 158.6, 154.2, 150.3, 130.0, 129.0, 127.6, 127.4, 122.9, 116.8, 104.3, 103.9, 99.5, 41.4.

### 2.3. Antioxidant Activity

#### 2.3.1. ABTS Assay

The ABTS assay was performed by generating the ABTS radical cation through the reaction of 7 mM ABTS with 2.45 mM potassium persulfate, followed by incubation in the dark for 12–16 h. The resulting solution was then diluted with ethanol or distilled water to an absorbance of 0.700 ± 0.020 at 734 nm. For each measurement, 160 µL of the diluted ABTS solution was combined with 40 µL of the test sample or standard antioxidant (concentrations of 1.5625 to 100 µM), incubated for 10 min in the dark, and the absorbance was recorded at 734 nm [17]. The inhibition percentage was calculated using Equation (1).

Inhibition (%) = (Absorbance Control − Absorbance Sample)/(Absorbance Control) × 100 (1)

#### 2.3.2. DPPH Assay

The DPPH assay was used to assess the free radical scavenging ability of the samples by monitoring the reduction of the stable DPPH radical. A DPPH solution was prepared by dissolving 4 mg of DPPH in 100 mL of methanol and stored in the dark at −20 °C; its absorbance was adjusted to 0.500 ± 0.020 before analysis. For each measurement, 160 µL of the DPPH solution was combined with 40 µL of the test compound or standard antioxidant (concentrations of 1.5625 to 100 µM), incubated for 30 min in the dark at room temperature, and the absorbance was recorded at 517 nm [18]. The inhibition percentage was calculated using Equation (1).

#### 2.3.3. FRAP Assay

The ferric reducing power of the compounds was assessed by measuring the conversion of Fe^3+^ to Fe^2+^ through the formation of a Prussian blue complex. For each measurement, 10 µL of the sample was mixed with 40 µL of phosphate buffer (pH 6.6) and 50 µL of 1% potassium ferricyanide (K_3_Fe(CN)_6_), then incubated at 50 °C for 20 min. Concentrations of 3.125–200 µM were used for I2, I4, I5, I6, A2, and ascorbic acid, whereas 0.390625–25 µM were used for the remaining compounds. After incubation, 50 µL of 10% trichloroacetic acid (TCA), 40 µL of distilled water, and 10 µL of 0.1% ferric chloride (FeCl_3_) were added, and the absorbance of the resulting mixture was measured at 700 nm using a 96-well microplate reader (PerkinElmer, Waltham, MA, USA) [19].

#### 2.3.4. Phenanthroline Assay

The phenanthroline assay was used to evaluate the ability of the samples to reduce Fe^3+^ to Fe^2+^, which forms an orange-colored complex with 1,10-phenanthroline, detected at 510 nm. For the assay, 10 µL of the sample (concentrations of 0.390625 to 25 µM) was mixed with 50 µL of 0.2% FeCl_3_ solution, 30 µL of 0.5% 1,10-phenanthroline solution, and 110 µL of methanol. The mixture was incubated in the dark for 20 min at 30 °C. Absorbance was then measured at 510 nm using a 96-well microplate reader [20].

#### 2.3.5. CUPRAC Assay

The cupric reducing antioxidant capacity (CUPRAC) assay evaluates the ability of antioxidants to reduce Cu^2+^-neocuproine complex to Cu^+^, forming a yellow-colored complex measurable at 450 nm. The reaction mixture consisted of 40 µL of the test sample (concentrations of 12.5 to 800 µM), 60 µL of ammonium acetate buffer (pH ≈ 7.0), 50 µL of 7.5 mM neocuproine solution and 50 µL of 1 mM CuCl_2_-2H_2_O solution. After incubation for 1 h at room temperature in the dark, the absorbance was measured at 450 nm [21].

#### 2.3.6. Statistical Analysis

All assays were performed in triplicate, and results are expressed as mean ± standard deviation. Statistical analysis was performed using R software (version 4.5.2). Normality of residuals and homogeneity of variances were assessed for each assay using Shapiro–Wilk and Levene’s tests, respectively. For the ABTS and FRAP assays, data were log-transformed prior to analysis to satisfy these assumptions. One-way analysis of variance (ANOVA) followed by Tukey’s honestly significant difference (HSD) post-hoc test was used to assess differences among the tested compounds for the DPPH, ABTS, FRAP, and CUPRAC assays. For the phenanthroline assay, normality could not be achieved even after transformation. Therefore, the non-parametric Kruskal-Wallis test was applied, followed by Dunn’s post-hoc test with Bonferroni correction. Statistical significance was set at α = 0.05. For statistical calculations, values exceeding the maximum tested concentration (reported as >200 µM) were conservatively treated as equal to 200 µM. Different letters indicate statistically significant differences among compounds.

### 2.4. Cytotoxicity Evaluation

#### 2.4.1. Cell Lines and Culture Conditions

The two cell lines used in this study, MDA-MB-231 (human triple-negative breast cancer adenocarcinoma cell line) and Vero (normal kidney cell line derived from an adult African green monkey), were sourced from ATCC and cultured in Dulbecco’s Modified Eagle Medium (DMEM) supplemented with 10% fetal bovine serum (FBS). No antibiotics were added to avoid potential interference with the assay. Cells were routinely tested for mycoplasma contamination using a commercial detection kit, MycoFluor™ Mycoplasma Detection Kit and were confirmed free of contamination prior to experiments. The cells exhibited a viability of approximately 98% before testing [22].

#### 2.4.2. Cell Seeding and Attachment

Cells were trypsinized with 0.25% trypsin-EDTA and counted using a hemocytometer or automated cell counter (Thermo Countess 3 FL). The cell suspension was diluted to a density of 2000 cells per well in 100 µL of culture medium and seeded into sterile 96-well flat-bottom microplates. Plates were incubated at 37 °C in a humidified atmosphere containing 5% CO_2_ for 12 h to allow for proper attachment [23].

#### 2.4.3. Treatment with Test Molecules

After 12 h of initial seeding, the culture medium was carefully aspirated, and the wells were washed with phosphate-buffered saline (PBS). Fresh medium containing test molecules was added to each well, with the molecules dissolved in dimethyl sulfoxide (DMSO). The final concentrations ranged from 400 μM to 12.5 μM, prepared by serial dilution. The maximum DMSO concentration used was 0.1%, which was confirmed not to affect cell viability by vehicle control experiments. Control wells containing medium with 0.1% DMSO (vehicle control) were included to account for any solvent effects. All treatments were performed in triplicate. The plates were then incubated for 36 h under standard cell culture conditions (37 °C, 5% CO_2_) [23].

#### 2.4.4. Cell Viability Assay (MTT)

After 36 h of incubation, 10 μL of a fresh solution of MTT reagent (3-(4,5-dimethylthiazol-2-yl)-2,5-diphenyltetrazolium bromide, 4 mg/mL in sterile PBS) was added to each well. The plates were further incubated for 6 h at 37 °C to allow formazan crystal formation. Subsequently, the medium and MTT solution were carefully aspirated, and 100 μL of 10% SDS in 0.01 M HCl was added to each well to solubilize the formazan crystals. Plates were gently shaken overnight at room temperature to ensure complete dissolution, then Absorbance was measured at 570 nm using a microplate reader. The optical density (OD) readings directly correlated with cell viability. The percentage viability was calculated relative to the vehicle control wells, which were set to 100% [23].

#### 2.4.5. Assessment of Potential Assay Interference by Test Compounds

To account for potential intrinsic reducing or chromogenic properties of the tested phenolic compounds, cell-free interference controls were integrated into the experimental design. Each compound was incubated with the MTT reagent under identical conditions as the primary assay, but in the absence of cells. Additionally, the intrinsic absorbance of each test compound alone (without cells or MTT reagent) was measured to establish baseline optical interference.

Raw absorbance values were corrected according to Equation (2):

A_corr_ = A_treated_ − A_comp+MTT_ − A_comp_ (2)

where A_corr_ is the corrected absorbance, A_treated_ is the absorbance measured for the treated cells, A_comp+MTT_ is the absorbance of the compound and MTT reagent in a cell-free system, and A_comp_ is the intrinsic absorbance of the compound.

Percentage cell viability was subsequently calculated relative to the corrected vehicle control absorbance using Equation (3):

(3) $$\%Viability=\frac{A_{corr,treated}}{A_{corr,control}}\times 100$$

### 2.5. DFT Calculations

All quantum-chemical calculations were performed using Gaussian 09 E.01 [24]. Full geometry optimizations of all studied compounds were carried out using the M06-2X hybrid meta-GGA functional in combination with the 6-311++G(d,p)basis set, which has been shown to provide reliable thermochemical data for radical reactions and antioxidant studies [25,26,27]. Solvent effects were taken into account using the SMD implicit solvation model, with water selected as the solvent to better approximate biological conditions [28]. Frequency calculations at the same level of theory were conducted to confirm that all optimized structures correspond to true minima (no imaginary frequencies) and to obtain zero-point energy and thermal corrections for the evaluation of thermodynamic parameters at 298.15 K.

To elucidate the possible antioxidant mechanisms, key thermodynamic descriptors associated with the hydrogen atom transfer (HAT), single-electron transfer–proton transfer (SETPT), and sequential proton loss–electron transfer (SPLET) pathways were calculated. The bond dissociation enthalpy (BDE), indicative of the HAT mechanism, was computed according to:

BDE = H(ArO^•^) + H(H^•^) − H(ArOH) (4)

The proton affinity (PA), which characterizes the first step of the SPLET mechanism, was calculated as:

PA = H(ArO^−^) + H(H^+^) − H(ArOH) (5)

The ionization potential (IP), which characterizes the first step of the SETPT mechanism, was calculated using the following expression:

IP = H(ArOH^•+^) + H(e−) − H(ArOH) (6)

In addition, the thermodynamic feasibility of radical scavenging was further assessed by computing the Gibbs free energy change (ΔG) for the reaction between the investigated compounds and the hydroperoxyl radical (HOO^•^). Frontier molecular orbitals (HOMO and LUMO) were calculated and analyzed to gain insight into the electronic properties governing redox behavior and antioxidant reactivity. Orbital energies and distributions were analyzed using Multiwfn 3.8 [29], and molecular structures and orbital isosurfaces were visualized using VMD 1.9.3 (Visual Molecular Dynamics) [30].

### 2.6. Docking Studies

**Figure 1:**
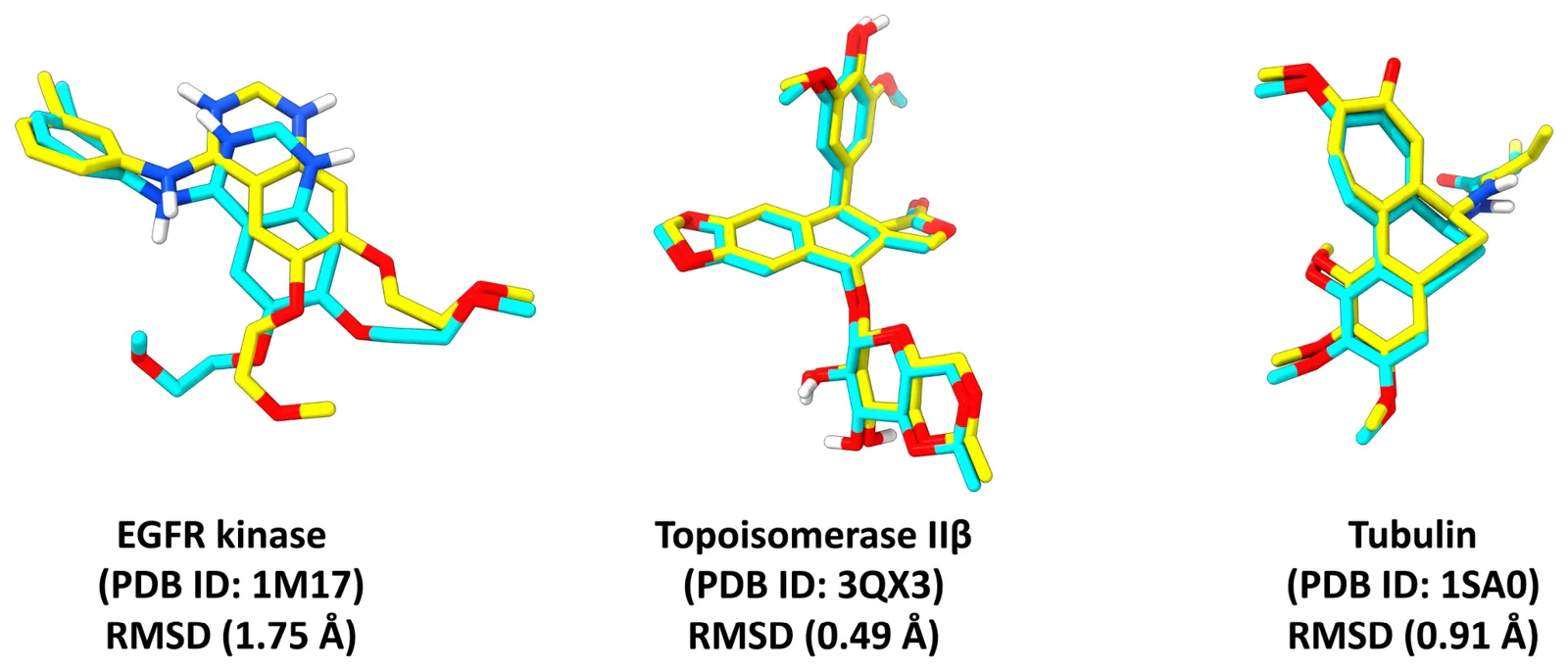
Validation of the molecular docking protocol by re-docking of the co-crystallized reference ligands into their respective binding sites. Superposition of the experimental crystal poses (yellow) and the corresponding re-docked conformations (cyan) for EGFR kinase (PDB ID: 1M17; RMSD = 1.75 Å), human Topoisomerase IIβ (PDB ID: 3QX3; RMSD = 0.49 Å), and Tubulin (PDB ID: 1SA0; RMSD = 0.91 Å).

Crystal structures of the EGFR kinase domain (PDB ID: 1M17), human Topoisomerase IIβ in complex with DNA and etoposide (PDB ID: 3QX3), and Tubulin at the colchicine-binding site (PDB ID: 1SA0) were retrieved from the Protein Data Bank. Protein structures were visually inspected, and all non-essential molecules, including crystallographic water molecules and co-crystallized ligands, were removed prior to docking, except for the DNA component retained in the Topoisomerase IIβ complex to preserve the integrity of the binding site. The molecular structure of the most promising compound I5 was optimized using density functional theory (DFT) at the M06-2X/6-311++G(d,p) level of theory with the SMD solvation model (water) using Gaussian 09, as described in the Computational Details section. The optimized geometry was subsequently used as input for molecular docking simulations. Protein and ligand preparation was performed using AutoDockTools v1.5.7 [5]. Polar hydrogens were added, AutoDock atom types were assigned, and Gasteiger partial charges were computed. The ligand was protonated to represent physiological pH conditions, rotatable bonds were defined, and all prepared structures were saved in PDBQT format. Molecular docking simulations were carried out using AutoDock Vina v1.2.7 [6], which employs an empirical scoring function combined with a stochastic global search algorithm. For all docking calculations, the exhaustiveness parameter was set to 40, with eight binding modes generated per ligand. Docking grid boxes were defined to fully encompass the active sites of each target protein based on reported binding regions. The grid parameters were set as follows: for EGFR, the grid center was positioned at (22.01, 0.25, 52.79 Å); for Topoisomerase IIβ, at (27.60, 103.64, 36.62 Å); and for Tubulin, at (41.09, 52.68, −9.12 Å), with grid dimensions of 20 × 20 × 20 Å^3^ for all targets Docking poses were ranked according to their predicted binding free energies (kcal/mol), and the top-ranked conformations were selected for further interaction analysis. Post-docking visualization and analysis of ligand–protein interactions were performed using Discovery Studio Visualizer (Dassault Systèmes BIOVIA) and UCSF Chimera [7]. To validate the docking protocol, reference ligands co-crystallized with each target—4-anilinoquinazoline for EGFR, etoposide for Topoisomerase IIβ, and colchicine for Tubulin—were re-docked into their respective binding sites using the same docking parameters. The re-docked poses successfully reproduced the experimental binding orientations with acceptable root-mean-square deviation (RMSD) values, confirming the reliability and accuracy of the applied docking methodology (Figure 1).

### 2.7. Molecular Dynamics Simulations

In order to gain additional insight into the I5 interactions with EGFR, Topoisomerase IIβ and Tubulin binding sites, molecular dynamics simulations were performed from the I5 docking pose. Molecular dynamics simulations of each system with their respective co-crystallized ligand were also performed under identical conditions to serve as a baseline for comparison. The original PDB files of 1M17, 3QX3 and 1SA0 crystal structures were processed using the Schrödinger Maestro suite (version 14.7.163) [31]. All crystallographic water molecules and co-crystallized ligands were removed, except for the DNA component in Topoisomerase IIβ and the Mg^2+^ atoms in Tubulin and Topoisomerase IIβ structures. All missing side chains and hydrogen atoms were automatically restored using the Protein Preparation tool (Schrödinger Suite 2026-1, Build 163) [32]. To avoid problems with the chain discontinuity of the original EGFR PDB structure, 1M17, its last 19 C-terminal amino acids, which are not directly connected to the main structure and do not play a relevant role in ligand binding, were removed. In parallel, each crystallographic reference ligand (4-anilinoquinazoline, etoposide and colchicine) was isolated from their respective PDB structures, retaining their original coordinates. LigPrep tool (Schrödinger Suite 2026-1, Build 163) was used to restore information about hydrogens, partial charges and bonds to the ligand, and then exported in mol2 format. Similarly, the I5 structure was converted from PDBQT to PDB format using PyMol 3.1.7, and then imported into the Schrödinger Maestro suite for preprocessing using LigPrep [33]. The prepared I5 structure was aligned and exported to mol2 format with each of the best predicted docking poses for the three targets, retaining the corresponding docking coordinates. A total of six molecular dynamics simulations were run up to 100 ns using GROMACS 2022.3 [34] on the processed EGFR, Topoisomerase IIβ and Tubulin structures, with their respective reference ligand and the best predicted I5 docking pose. MD simulations were carried out on the cajal HPC cluster (UCAM) using a GPU (NVIDIA GeForce RTX 3090) and 8 GB RAM. Firstly, each ligand topology was generated using ACPYPE (version 2015-11-02 Rev: 8810) [35,36], with the mol2 ligand files as input. The protein topologies were generated using the gmx pdb2gmx command, with the AMBER99sb force field for the EGFR and Tubulin systems, and the AMBER99bsc1 force field for Topoisomerase IIβ systems, which is more suitable for DNA simulations. The simulation box was set to a dodecahedron for EGFR and Topoisomerase systems, and triclinic for the Tubulin system in order to significantly reduce the box volume. The systems were solvated using the TIP3P water model and neutralized with ions, with a minimum distance of 0.9 nm between the protein surface and the box boundary for EGFR and Topoisomerase simulations, and 1.0 nm for Tubulin. Systems were energy-minimized, equilibrated in the NVT and NPT ensembles at 300 K (one 400 ps NVT phase, followed by four sequential 400 ps NPT phases), and simulated for 100 ns with a 2fs time step, LINCS constraints [37] on all bonds, and long-range electrostatics treated by the particle mesh Ewald (PME) method. The AMBER ff99SB force [38] field was used for protein–ligand (EGFR-I5, EGFR- 4-anilinoquinazoline, Tubulin-I5 and Tubulin-colchicine) systems and AMBER ff99bsc1 [39] for the protein–DNA–ligand (Topoisomerase IIβ-I5 and Topoisomerase IIβ-etoposide) system. The results obtained by the MD simulation were analyzed using ASGARD “https://github.com/bio-hpc/ASGARD (accesed on 5 June 2026)”, an in-house tool developed by the BIO-HPC group [40]. For each system, structural stability and flexibility (RMSD, RMSF, dssp and radius of gyration) and ligand–protein interactions (Electrostatic and Lennard-Jones interactions, hydrogen bonds and MM-PBSA) were analyzed. For the Topoisomerase IIβ-I5 systems, protein–DNA and ligand–DNA interactions were also extracted. MMPBSA analysis was performed by the g_mmpbsa tool (version 5.1.2) [41].

## 3. Results and Discussion

### 3.1. Synthesis

**Figure 2:**
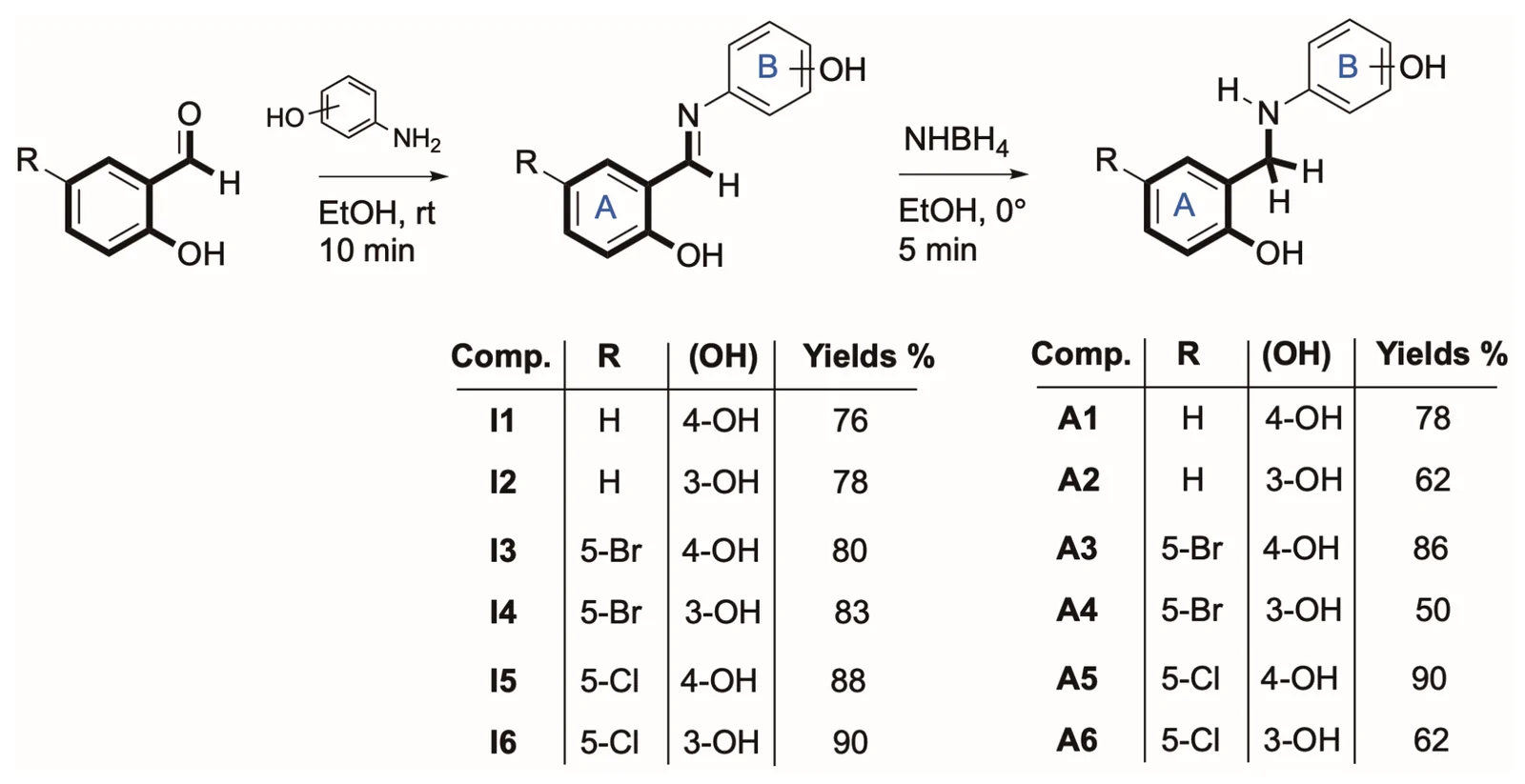
Two-step synthetic route employed for the synthesis of phenolic Schiff bases I1–I6 and their reduced secondary amines A1–A6, showing the chemical structures of all compounds and their corresponding isolated yields.

The target phenolic Schiff bases (I1–I6) and their corresponding secondary amines (A1–A6) were synthesized via a two-step procedure following a previously reported protocol [16], as outlined in Figure 2. Notably, some of the synthesized compounds have been previously described in the literature, and the spectral data obtained in this study are in full agreement with the reported values, thereby confirming their structural identity [42,43,44,45,46,47]. The final isolated yields ranged from 50% to 90%. Overall, the Schiff bases (I1–I6) were obtained in good to excellent yields (76–90%), with the non-halogenated derivatives (I1 and I2) affording the lowest yields (76–78%), while the halogenated analogs (I3–I6) were obtained in higher yields (80–90%). In contrast, the yields of the reduced secondary amines were found to depend markedly on the position of the phenolic hydroxyl group. Amines bearing the hydroxyl group in the para position (A1, A3, and A5) were obtained in higher yields (78–90%), whereas the corresponding meta-hydroxyl derivatives (A2, A4, and A6) gave significantly lower yields (50–62%). The molecular structures of all synthesized compounds were confirmed by ^1^H NMR, ^13^C NMR, and FTIR spectroscopy, with the obtained spectral features consistent with those reported in the literature.

### 3.2. In Vitro Antioxidant Activity Evaluation

**Figure 3:**
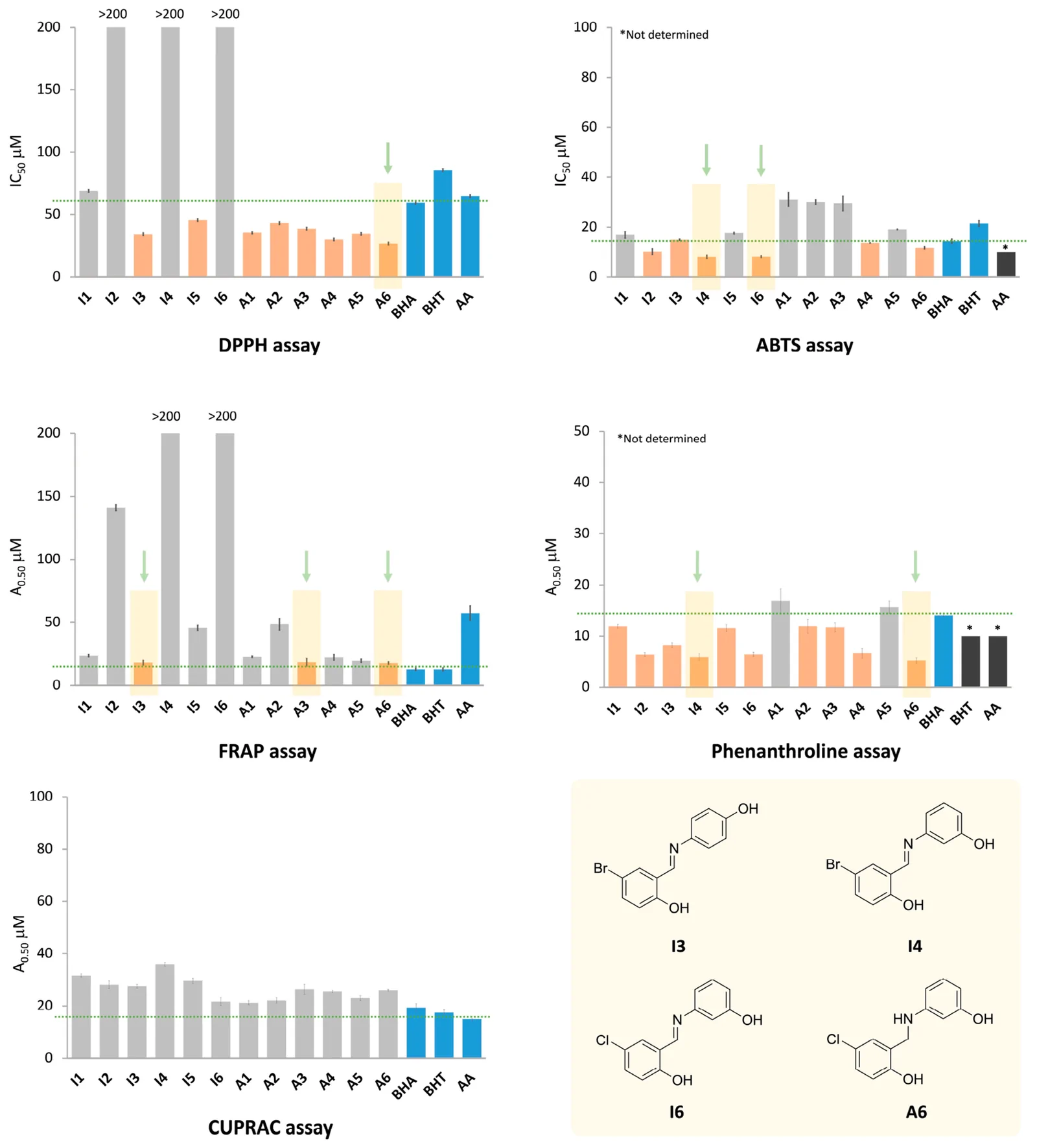
Antioxidant activities of the synthesized imine (I1–I6) and secondary amine (A1–A6) compounds evaluated using DPPH, ABTS, FRAP, phenanthroline, and CUPRAC assays, expressed as IC_50_ or A_0.5_ values. BHA, BHT, and ascorbic acid were used as reference antioxidants. The green dashed line indicates the reference standard’s activity. Arrows and shaded areas highlight the most active synthesized compounds, whose structures are shown in the bottom right panel.

To evaluate the antioxidant activity of the twelve synthesized compounds, five complementary assays were employed. The DPPH and ABTS assays measure the radical-scavenging capacity of compounds toward stable free radicals, with DPPH active in organic media and ABTS applicable in both aqueous and organic solvents. The FRAP and CUPRAC methods assess the ability of antioxidants to reduce Fe^3+^ and Cu^2+^ ions, respectively, the former under acidic conditions and the latter at neutral pH. Finally, the phenanthroline assay is based on the reduction of Fe^3+^ to Fe^2+^, detected through the formation of an Fe^2+^-phenanthroline complex, which involves both reduction and metal chelation. Statistical analysis across all assays was performed using R software (version 4.5.2). After verifying normality and homogeneity of variances—applying log-transformation to the ABTS and FRAP data where necessary—a one-way ANOVA confirmed highly significant differences among the tested compounds (*p* < 0.001). Subsequently, Tukey’s HSD post-hoc test was utilized to classify the compounds into statistically distinct activity groups. For the phenanthroline assay, normality could not be achieved even after transformation, so a Kruskal–Wallis test was used instead, followed by Dunn’s post hoc test with Bonferroni correction. The results, expressed as IC_50_ or A_0.5_ values, are presented in Figure 3 and Table S1.

The DPPH radical scavenging assay revealed a pronounced variability in antioxidant reactivity among the tested compounds, with IC_50_ values ranging from 26.7 ± 0.97 to 342.26 ± 4.24 µM, indicating a strong dependence of activity on molecular structure and functional group nature. Overall, the secondary amines exhibited significantly higher antioxidant activity than the imine derivatives, as evidenced by their consistently lower IC_50_ values. This trend highlights the potential role of the secondary amine (–NH–) functionality in enhancing radical scavenging efficiency, likely through improved hydrogen-donating ability and stabilization of the resulting radical. Among all tested molecules, A6 (26.7 ± 0.97 µM) and A4 (29.95 ± 0.63 µM) were identified as the most potent DPPH scavengers, followed by A1 (35.45 ± 0.63 µM), A5 (34.42 ± 0.75 µM), and A3 (38.81 ± 0.64 µM). In contrast, the corresponding imine derivatives, particularly I2 (340.8 ± 0.9 µM), I4 (322.95 ± 2.07 µM), and I6 (342.26 ± 4.24 µM), displayed markedly weaker activity. Comparison with reference antioxidants further emphasizes the efficiency of the amine derivatives. Notably, A6 and A4 exhibited stronger DPPH scavenging activity than BHA (59.39 ± 1.9 µM), BHT (85.85 ± 3.69 µM), and ascorbic acid (64.98 ± 1.84 µM).

The ABTS scavenging assay revealed a distinct antioxidant profile compared to the DPPH results, with IC_50_ values ranging from 8.15 ± 0.62 µM to 31.1 ± 2.79 µM, indicating overall stronger reactivity toward the ABTS radical cation. In contrast to the DPPH assay, the imine derivatives exhibited superior ABTS scavenging activity compared to the secondary amines. The most active compounds were I4 (8.15 ± 0.62 µM) and I6 (8.23 ± 0.33 µM), followed closely by I2 (10.18 ± 1.1 µM) and A6 (11.69 ± 0.43 µM). A relatively moderate activity was observed for I3 (14.89 ± 0.22 µM), I1 (16.88 ± 1.35 µM), and I5 (17.57 ± 0.41 µM), while most secondary amines (A1–A3) showed weaker scavenging ability, with IC_50_ values around 29–31 µM, indicating limited effectiveness in this assay. Comparison with the reference antioxidants further emphasizes the strong performance of the imine derivatives. Notably, I4, I6, and I2 exhibited significantly higher ABTS scavenging activity than BHA (14.31 ± 0.85 µM) and BHT (21.49 ± 1.13 µM). The A6 derivative, on the other hand, also demonstrated a scavenging ability higher than that of BHA and BHT.

The FRAP assay revealed A_0.5_ values ranging from 12.80 ± 1.15 to >200 µM. Overall, the secondary amines demonstrated superior ferric reducing activity compared to the imine derivatives. Most secondary amines exhibited A_0.5_ values between 17.76 ± 0.81 and 48.53 ± 4.6 µM, whereas several imine derivatives, notably I4 and I6, showed very weak activity with A_0.5_ values exceeding 200 µM, reflecting limited Fe^3+^ reduction capability. Among all tested compounds, A6 (17.76 ± 0.81 µM), A3 (18.43 ± 2.83 µM), A5 (19.53 ± 1.29 µM), and I3 (18.08 ± 1.76 µM) emerged as the most effective reducers, exhibiting A_0.5_ values comparable to those of the reference antioxidants BHA (12.83 ± 0.86 µM) and BHT (12.80 ± 1.15 µM). In contrast, ascorbic acid (57.38 ± 5.75 µM) displayed weaker reducing power under these experimental conditions, being outperformed by most imine compounds.

The phenanthroline assay showed A_0.5_ values ranging from 5.25 ± 0.5 µM to 16.89 ± 2.4 µM, indicating generally strong reducing capacity across the series. Compound A6 (5.25 ± 0.50 µM) exhibited the most pronounced reducing activity, significantly outperforming A1 (16.89 ± 2.40 µM) and A5 (15.69 ± 1.17 µM), which showed the weakest activity in this assay. The remaining compounds, including I4 (5.89 ± 0.62 µM), I2 (6.38 ± 0.37 µM), I6 (6.41 ± 0.38 µM), A4 (6.68 ± 0.95 µM), I3 (8.27 ± 0.45 µM), I5 (11.54 ± 0.67 µM), I1 (11.90 ± 0.36 µM), A2 (11.94 ± 1.37 µM), A3 (11.71 ± 0.86 µM), and the reference antioxidant BHA (14.09 ± 0.21 µM), displayed intermediate A_0.5_ values that did not differ significantly from one another.

The CUPRAC assay revealed A_0.5_ values ranging from 15.0 ± 0.99 µM to 35.94 ± 0.69 µM, reflecting differences in Cu^2+^-reducing capacity under near-physiological conditions. This assay predominantly probes single-electron transfer (SET) mechanisms. Overall, the secondary amines exhibited superior or comparable CUPRAC activity relative to the imines. The most potent compounds were I6 (21.67 ± 1.51 µM), A1 (21.26 ± 0.78 µM), and A2 (22.14 ± 1.03 µM), with activities close to those of the reference antioxidants BHA (19.37 ± 1.54 µM), BHT (17.48 ± 0.97 µM), and ascorbic acid (15.0 ± 0.99 µM). Moderate reducing capacity was observed for A5 (23.05 ± 0.96 µM), A4 (25.49 ± 0.5 µM), A6 (25.98 ± 0.35 µM), and A3 (26.34 ± 1.89 µM), whereas most imine derivatives, particularly I1 (31.69 ± 0.63 µM) and I4 (35.94 ± 0.69 µM), showed weaker activity.

Taken together, the results obtained from the five complementary antioxidant assays clearly demonstrate an assay-dependent structure–activity relationship governed by the nature of the nitrogen functionality. The secondary amines consistently exhibited superior performance in the DPPH assay, where several derivatives (notably A4 and A6) outperformed classical antioxidants like BHA, BHT, and ascorbic acid, and showed the strongest reducing power among all tested compounds in the FRAP assay, although still significantly less active than BHA and BHT. Notably, in the phenanthroline assay, A6 was the only compound to significantly outperform the reference antioxidant BHA. In contrast, the imines showed enhanced efficiency in the ABTS assay, where multiple imine derivatives displayed lower IC_50_ values than the reference standards. The CUPRAC assay revealed intermediate behavior, with secondary amines generally matching or slightly surpassing imines. These findings suggest that the synthesized compounds exhibit distinct redox profiles depending on the specific testing environment, and their reactivity is influenced by the nature of the nitrogen functionality. This antioxidant behavior underscores the importance of functional group modulation in tailoring redox properties and validates the use of multiple assays to accurately assess antioxidant potential.

### 3.3. Computational Evaluation of the Antioxidant Properties

#### 3.3.1. Structural Analysis

**Figure 4:**
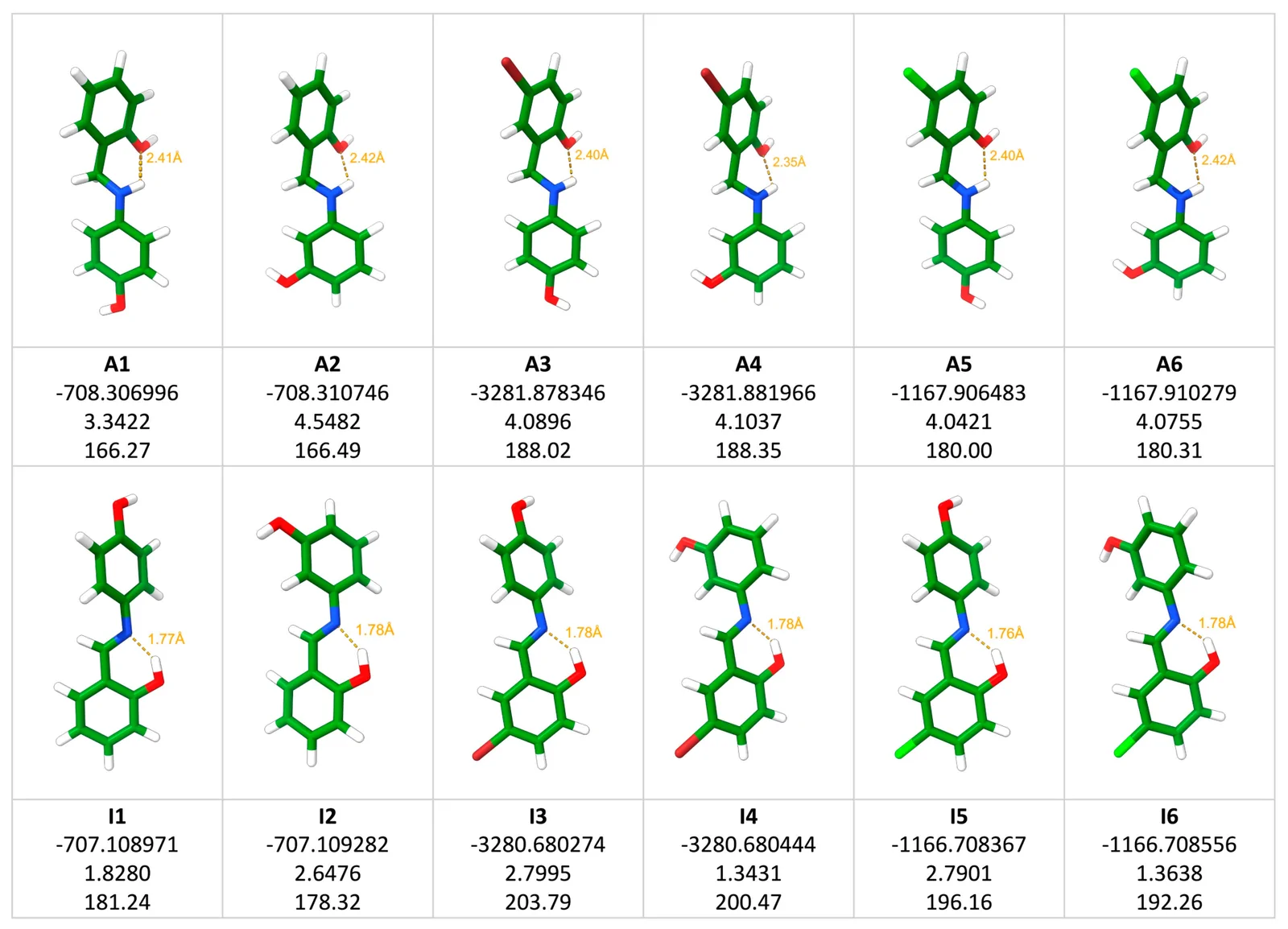
Optimized molecular geometries of the amine derivatives (**A1**–**A6**) and their corresponding imine analogs (**I1**–**I6**) obtained at the M06-2X/6-311++G(d,p) level of theory, together with the calculated total electronic energies (a.u.), dipole moments (D), and isotropic polarizabilities (a.u.).

To elucidate the radical scavenging properties of the synthesized compounds, a computational study was carried out at the M06-2X/6-311++G(d,p) level of theory, focusing on molecular geometry, chemical reactivity, and key electronic descriptors relevant to antioxidant activity. The initial step consisted of identifying the most stable conformations of all compounds, which were subsequently used to assess their chemical reactivity. The optimized geometries of all the studied compounds, along with total electronic energies, dipole moments, and isotropic polarizabilities, are shown in Figure 4. Geometry optimization reveals that all compounds adopt non-planar conformations stabilized by intramolecular hydrogen bonding involving the nitrogen center and neighboring OH substituent. However, a marked difference is observed between the two series. In the amine derivatives, the N–H···O interaction distances fall in the range of approximately 2.35–2.42 Å, indicative of moderate hydrogen bonding. In contrast, the imine analogs exhibit significantly shorter interaction distances (1.76–1.78 Å), reflecting stronger intramolecular interactions. This effect is evident for all the imine and amine analogs, demonstrating that the imine functionality promotes a more rigid and internally stabilized molecular framework. The calculated dipole moments further differentiate the two series. Amine derivatives exhibit higher dipole moments (approximately 3.3–4.5 D), consistent with the increased polarity and conformational flexibility of the C–NH group. In contrast, the imine analogs show lower dipole moments (about 1.3–2.8 D), reflecting more effective internal charge delocalization. In addition, the imine series consistently displays higher isotropic polarizabilities than the corresponding amines, indicating greater electronic flexibility and an enhanced ability to stabilize charge or spin density.

**Figure 5:**
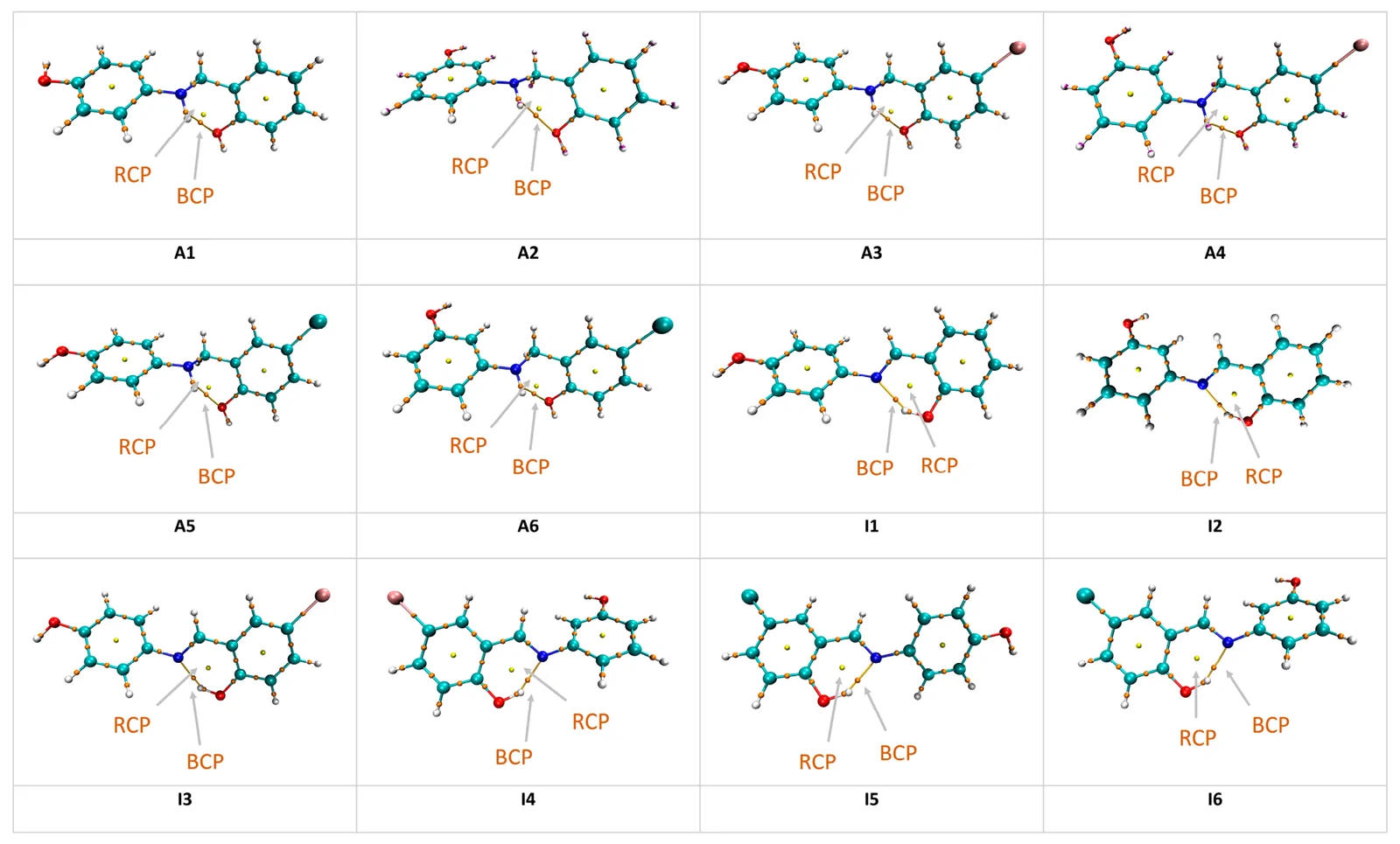
Topological molecular graphs of the optimized amines (**A1**–**A6**) and imines (**I1**–**I6**) derivatives showing the locations of the ring critical points (RCP, type (3, +1)) and bond critical points (BCP, type (3, −1)) associated with the intramolecular interactions, as identified by Atoms in Molecules (AIM) analysis at the M06-2X/6-311++G(d,p) level of theory.

To further characterize the nature and strength of the intramolecular interactions identified in the optimized structures, an Atoms in Molecules (AIM) analysis was performed. AIM theory, developed by Bader, provides a rigorous topological description of the electron density and enables quantitative identification of bonding interactions through the analysis of critical points and associated electron density properties [48]. The results of the AIM analysis are summarized in Table 1 and illustrated by the topological molecular graphs in Figure 5. For all compounds, the presence of a bond critical point (BCP, type (3, −1)) together with an associated ring critical point (RCP, type (3, +1)) confirms the existence of a well-defined intramolecular interaction. The BCPs are characterized by relatively low electron density values, with ρ(r) ranging from 0.011 to 0.012 a.u. for the amine derivatives and 0.044–0.046 a.u. for the imine analogs, accompanied by positive Laplacians of the electron density (∇^2^ρ ≈ 0.043–0.048 a.u. for amines and 0.116–0.117 a.u. for imines), consistent with closed-shell, noncovalent interactions. The corresponding RCPs display lower electron density values (ρ(r) ≈ 0.010–0.011 a.u. for amines and 0.017–0.018 a.u. for imines) and positive Laplacians (∇^2^ρ ≈ 0.051–0.056 a.u. and 0.111–0.113 a.u., respectively), reflecting the topological signature of the intramolecular ring formed upon interaction. Notably, the imine derivatives exhibit more negative total energy densities at the BCPs (H(r) ≈ −0.006 to −0.007 a.u.) compared to the amine series (H(r) ≈ +0.001–0.002 a.u.), indicating stronger and more stabilized intramolecular interactions in the imine analogs. These trends are consistent across all compounds and correlate well with the optimized geometries.

#### 3.3.2. Reactivity Descriptors

**Figure 6:**
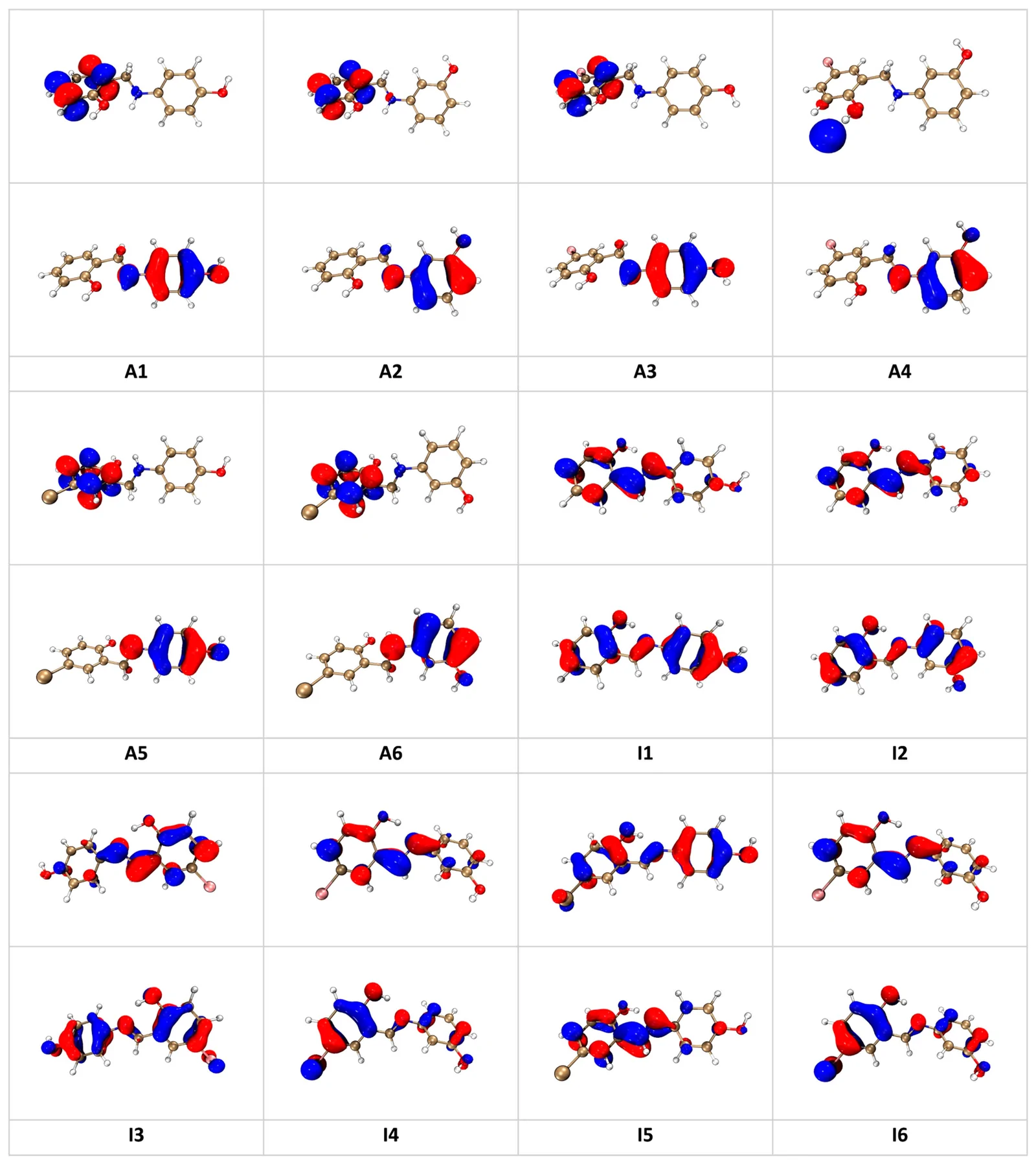
Frontier molecular orbitals (HOMO and LUMO) distributions of the optimized amine (**A1**–**A6**) and imine (**I1**–**I6**) derivatives calculated at the M06-2X/6-311++G(d,p) level of theory.

To further elucidate the electronic structure and reactivity of the synthesized compounds, their electronic properties were investigated through analysis of the frontier molecular orbitals (FMOs) and a set of global reactivity descriptors derived from density functional theory calculations. The energies and spatial distributions of the highest occupied molecular orbital (HOMO) and lowest unoccupied molecular orbital (LUMO) are depicted in Figure 6, while the corresponding HOMO and LUMO energies, together with the calculated ionization potential (I), electron affinity (A), chemical hardness (η), chemical potential (μ), electronegativity (χ), global softness (σ), electrophilicity index (ω), and nucleophilicity index (ξ), are summarized in Table 2.

As shown in Figure 6, the spatial distributions of the frontier molecular orbitals differ markedly between the amine and imine series. For all amine derivatives, the HOMO is predominantly localized on the aniline moiety (ring B), indicating that this fragment acts as the primary electron-donating region of the molecule. In contrast, the corresponding LUMO is mainly distributed over the salicylaldehyde moiety, reflecting an intrinsic separation between electron-rich and electron-deficient regions within the amine framework. Upon imine formation, this orbital localization pattern changes significantly; in the imine derivatives, both the HOMO and LUMO are largely delocalized over the entire molecular skeleton. This enhanced delocalization is consistent with increased π-conjugation across the molecule and suggests improved charge-transfer capability in the imine series.

The data reported in Table 2 provide insight into the electronic properties of the studied compounds. The amine derivatives, characterized by relatively higher HOMO energies (−6.48 to −6.85 eV) and larger nucleophilicity indices (ξ ≈ 0.47–0.53 eV), exhibit enhanced electron-donating ability, which is favorable for both hydrogen atom transfer (HAT) and electron-transfer processes involved in radical scavenging. In contrast, the imine analogs display lower HOMO energies (−7.26 to −7.59 eV) but significantly higher electron affinities (1.04–1.42 eV) and electrophilicity indices (ω ≈ 2.77–3.28 eV), indicating an improved capacity to stabilize incoming electron density. This electronic profile suggests that the imine derivatives are better suited to single-electron transfer (SET) mechanisms. The comparable HOMO–LUMO gaps observed for both series indicate similar overall kinetic stability, while the differences in charge-transfer descriptors rationalize the distinct antioxidant pathways favored by the amine and imine compounds.

**Figure 7:**
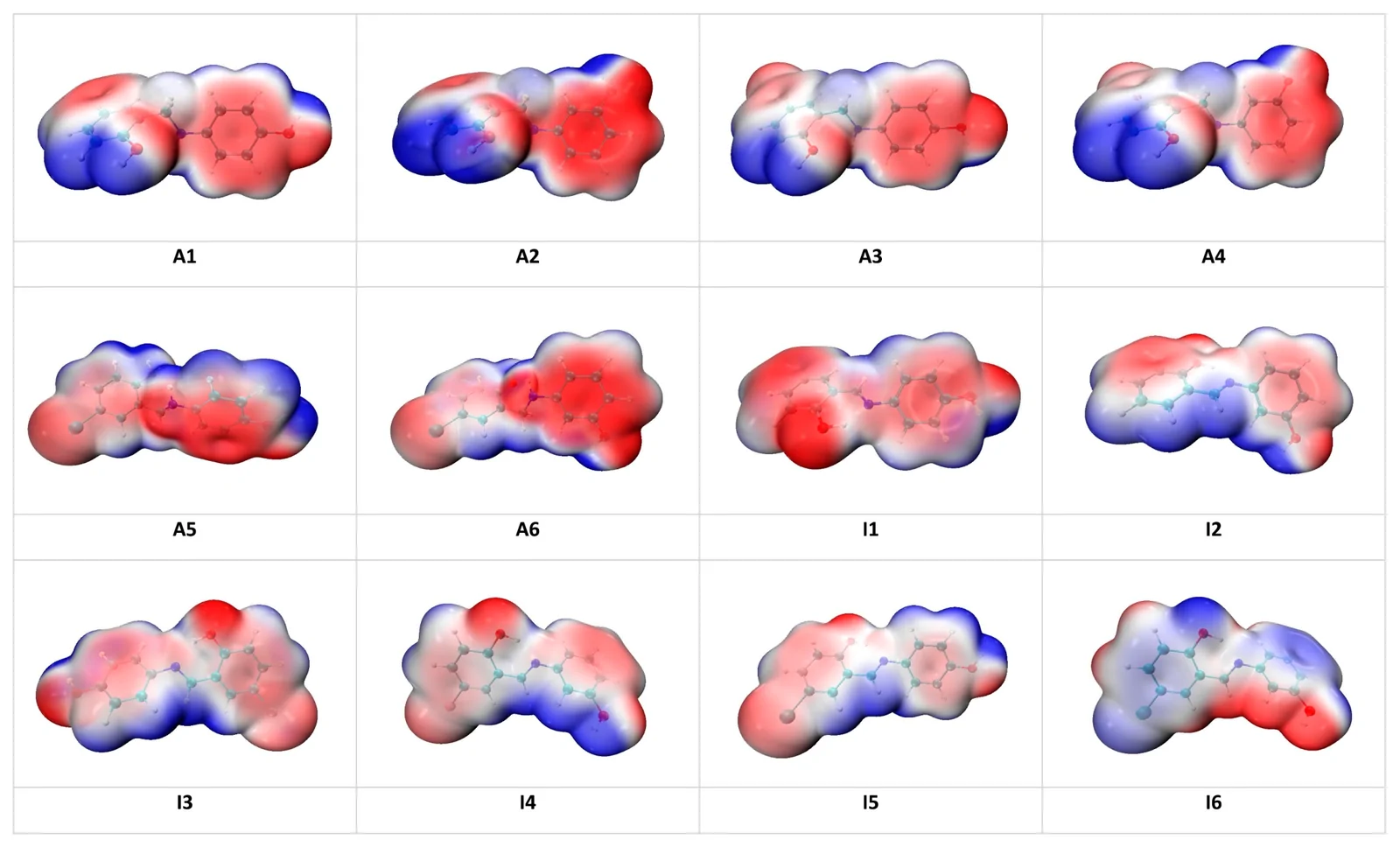
Molecular electrostatic potential (ESP) maps of the optimized amine (**A1**–**A6**) and imine (**I1**–**I6**) derivatives calculated at the M06-2X/6-311++G(d,p) level of theory. Regions of negative electrostatic potential (red) and positive electrostatic potential (blue) indicate electron-rich and electron-deficient sites, respectively.

The molecular electrostatic potential (ESP) maps shown in Figure 7 provide further insight into the charge distribution and reactive sites of the amine and imine derivatives. For the amine series (A1–A6), regions of negative electrostatic potential (red) are predominantly localized on the phenolic oxygen atoms, while positive potential regions (blue) are mainly associated with the amino hydrogen and adjacent aromatic framework. This clear separation of electron-rich and electron-deficient regions suggests preferential sites for electrophilic and radical attack and supports hydrogen atom donation from the phenolic group during radical scavenging. In contrast, the imine derivatives (I1–I6) display a more delocalized electrostatic potential distribution over the entire molecular framework. The negative potential is not confined to a single functional group but extends across the conjugated π-system, including the imine linkage, reflecting enhanced electronic delocalization.

#### 3.3.3. Evaluation of the Antioxidant Mechanisms

**Figure 8:**
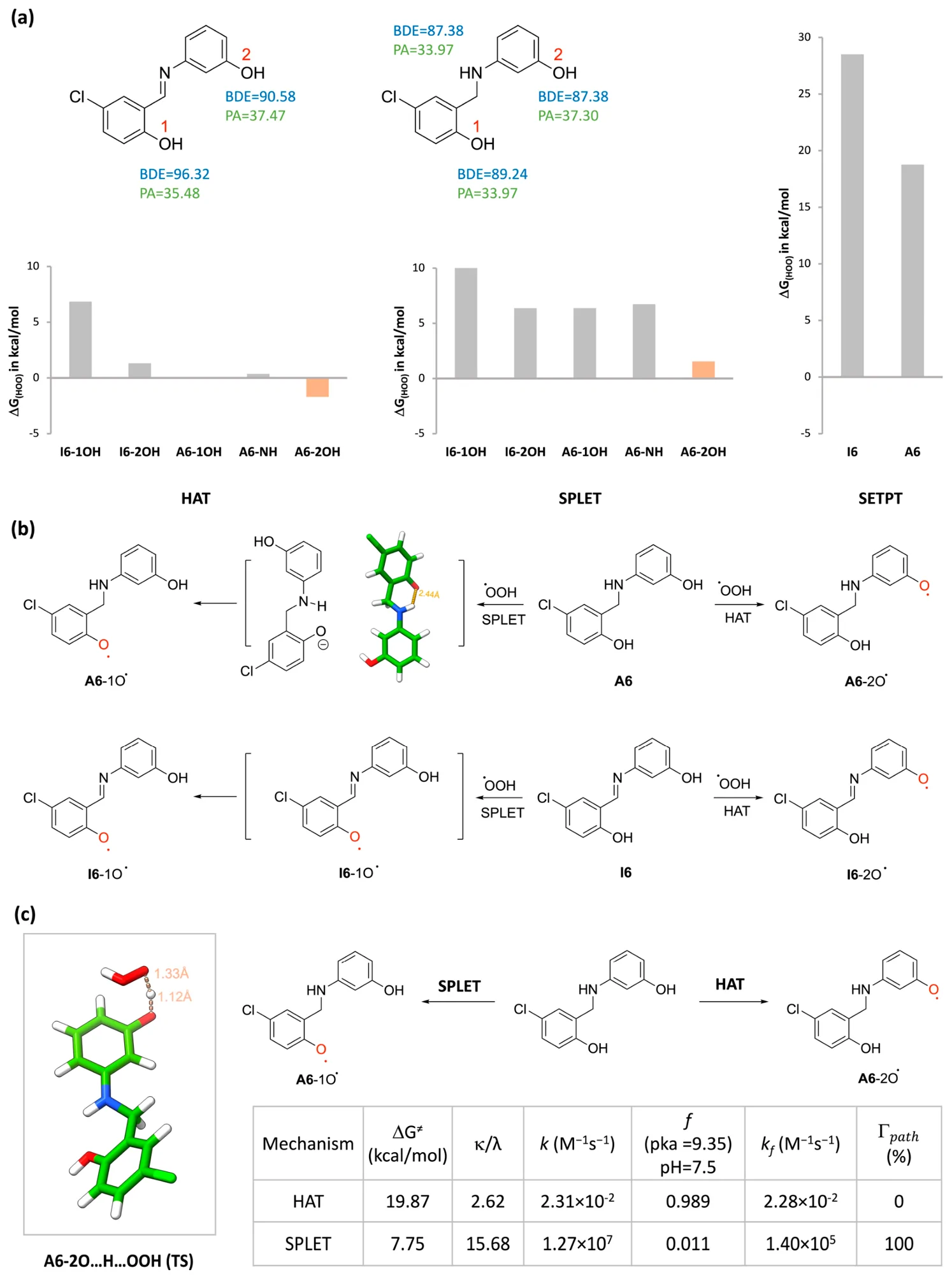
(**a**) Calculated thermodynamic descriptors and reaction free energies for the antioxidant mechanisms of the most active derivatives A6 and I6 in aqueous solution. (**b**) The proposed preferred scavenging routes and the corresponding radical intermediates. (**c**) Kinetic data for the reactions of A6 with HOO radical.

To better understand the antioxidant behavior of the studied compounds, we investigated the likely antioxidant mechanisms of the most active derivative, the amine A6 and its imine analog I6. Two key thermodynamic descriptors, the bond dissociation enthalpy (BDE) and the proton affinity (PA), were calculated for both compounds while considering the effect of an aqueous environment. In addition, the Gibbs free energies (ΔG) for their reactions with the HOO· radical, used as a model reactive oxygen species (ROS), were computed in water for the three main antioxidant pathways: hydrogen atom transfer (HAT), sequential proton loss–electron transfer (SPLET), and sequential electron transfer–proton transfer (SETPT). The results are summarized in Figure 8.

The calculated BDE values show that hydrogen abstraction is generally more favorable for A6 than for I6, particularly at the 2-OH site, where A6 displays the lowest BDE (≈87.4 kcal/mol), indicating an enhanced ability to donate a hydrogen atom. In contrast, I6 exhibits higher BDE values (≈90.6–96.3 kcal/mol), suggesting a less favorable HAT process. Consistently, the computed ΔG(HOO·) values for HAT indicate that A6 is thermodynamically more reactive, with the 2-OH position being the most favorable and slightly exergonic, whereas I6 remains endergonic at both hydroxyl sites. The PA results further support the higher antioxidant potential of A6 under polar conditions. The lower PA values obtained for A6 (≈34.0–37.3 kcal/mol) indicate that deprotonation is more accessible compared with I6, especially at the most reactive hydroxyl group. Nevertheless, the SPLET pathway remains overall endergonic for both compounds in water, as shown by the positive ΔG values obtained for all investigated positions. This suggests that SPLET is not the thermodynamically preferred route for HOO· scavenging under the present conditions. While thermodynamic parameters evaluate the overall energy states of the reaction, they do not exclusively dictate the dominant active pathway in polar media. Although SPLET is not the most favored route thermodynamically for A6 under these conditions, it can entirely dominate kinetically due to significantly lower activation energy barriers, as reported for several natural antioxidants [49,50]. On the other hand, the SETPT pathway is clearly the least favorable for both derivatives, as indicated by the large positive ΔG values (≈29 kcal/mol for I6 and ≈19 kcal/mol for A6).

A comparison between the two aromatic fragments, ring A (salicylaldehyde moiety) and ring B (aniline moiety), reveals distinct reactivity patterns toward the different antioxidant pathways for both I6 and A6. The calculated PA values indicate that the hydroxyl group on ring A is the easiest site to deprotonate, suggesting that this ring is the most prone to initiate the SPLET mechanism. In contrast, the hydroxyl group on ring B consistently shows the lowest BDE among all examined sites, indicating that this position is the most favorable for HAT and therefore represents the main hydrogen-donating center. For the NH group, the computed BDE is comparable to that of the OH group; however, the corresponding ΔG(HOO·) values are more favorable for hydrogen abstraction from the OH site (0.36 vs. −1.70 kcal/mol), confirming that the OH group is thermodynamically more reactive than the NH group in water. Regarding deprotonation, our calculations further show that removal of the proton from the NH group is accompanied by an intramolecular proton transfer from the adjacent ring A–OH to the nitrogen atom, leading to an anionic species localized on the oxygen atom. As a result, the PA values associated with deprotonation at the NH and ring A–OH sites become essentially identical, reinforcing that the ring A–OH bond is the most susceptible to deprotonation. Based on these thermodynamic results, the most plausible antioxidant pathways for both A6 and I6 in water are proposed in Figure 8.

To further refine this mechanistic interpretation and explicitly distinguish between thermodynamic feasibility and kinetic dominance, we next performed kinetic calculations for the most active compound A6. As a first step, we evaluated the acid–base properties of this phenolic system by computing its pKa value in water using a well-established protocol [51]. This point is particularly important because the acid–base equilibrium directly controls the availability of the deprotonated species, which is a key prerequisite for the SPLET mechanism [52,53]. The obtained pKa value and the corresponding molar fractions of the neutral and dissociated forms of A6 in aqueous solution are shown in Figure 8. As we can see, A6 exhibits a calculated pKa of 9.35, indicating that the neutral form is the dominant species in water at physiological pH, representing approximately 98.9% of the total population, while only a small fraction of the deprotonated form is present (1.1%). This minor proportion is not expected to significantly influence the overall reactivity through the HAT mechanism, which mainly involves the neutral species. However, even at low concentration, the deprotonated form is the key reactive species for the SPLET mechanism, since deprotonation is a prerequisite for this pathway. Therefore, the neutral species was considered for the HAT mechanism, whereas the dissociated species was used to describe the SPLET mechanism.

Rate constants were determined using conventional transition state theory (TST) at 298.15 K under a 1 M standard state, according to Equation (7):

(7) $$k=\sigma \kappa \frac{k_{B}T}{h}e^{-({\Delta G}^{\neq })/RT}$$

where σ is the reaction symmetry number [54,55], κ is the tunneling correction factor evaluated using the Eckart barrier model [56], k_B_ is the Boltzmann constant, h is Planck’s constant, and ΔG^‡^ is the Gibbs free energy of activation.

To estimate the relative contribution of competing pathways, branching ratios (Γ, %) were calculated using Equation (8):

(8) $$\Gamma_{path}=\frac{k}{k_{overall}}\times 100$$

For single-electron transfer (SET) processes, activation free energies were estimated within the framework of Marcus theory using Equation (9):

(9) $${∆G}_{SET}^{\neq }=\frac{\lambda }{4}{\left(1+\frac{{∆G}_{SET}^{0}}{\lambda }\right)}^{2}$$

where ${∆G}_{SET}^{0}$ is the Gibbs free energy of reaction and λ is the reorganization energy, approximated according to Equation (10):

(10) $$\lambda \approx {\Delta E}_{SET}+{∆G}_{SET}^{0}$$

where ${\Delta E}_{SET}$ corresponds to the vertical nonadiabatic energy difference between the neutral reactants and their oxidized or reduced states at the reactant geometry.

The computed rate constants for the HAT and SPLET mechanisms are summarized in Figure 8, together with the corresponding optimized transition-state structure for the HAT pathway. The results show that the HAT reaction proceeds with a slow rate constant of 2.28 × 10^−2^ M^−1^ s^−1^, indicating that HAT is not a kinetically significant process for A6. This behavior can be attributed to the high activation free-energy barrier (ΔG^‡^ = 19.87 kcal mol^−1^), which markedly slows the reaction. In contrast, the calculated rate constant for the SPLET pathway is 1.40 × 10^5^ M^−1^ s^−1^, which is several orders of magnitude higher than that of HAT, suggesting that A6 predominantly scavenges radicals via SPLET rather than HAT in aqueous solution. Importantly, the overall rate constant for A6 is comparable to that of BHT (k = 2.51 × 10^5^ M^−1^ s^−1^ [57]) and slightly higher than that of Trolox (k = 8.96 × 10^4^ M^−1^ s^−1^ [58]), indicating that A6 is a potent radical scavenger in polar media.

### 3.4. Cytotoxicity Evaluation

The cytotoxicity of the synthesized compounds was subsequently evaluated against the human triple-negative breast cancer (TNBC) cell line MDA-MB-231, alongside noncancerous Vero cells. Compounds were tested at six concentrations ranging from 12.5 to 400 μM, and cell viability was quantified relative to untreated controls. IC_50_ values were determined using four-parameter logistic regression (DoseResp model), where dose–response curves exhibited monotonic behavior [59,60]. Prior to evaluating the cytotoxicity, cell-free controls confirmed that the compounds do not react directly with the MTT reagent, ensuring the results reflect actual cell death.

MDA-MB-231 is a highly aggressive and invasive TNBC model characterized by the absence of estrogen receptor (ER), progesterone receptor (PR), and HER2 expression, making it resistant to hormone-based and HER2-targeted therapies [61]. This cell line exhibits high metastatic capacity and is widely employed as a clinically relevant in vitro model for screening novel anticancer agents targeting difficult-to-treat breast cancer subtypes [62,63]. Consequently, activity against MDA-MB-231 cells is considered indicative of potential therapeutic relevance for aggressive breast cancers.

**Figure 9:**
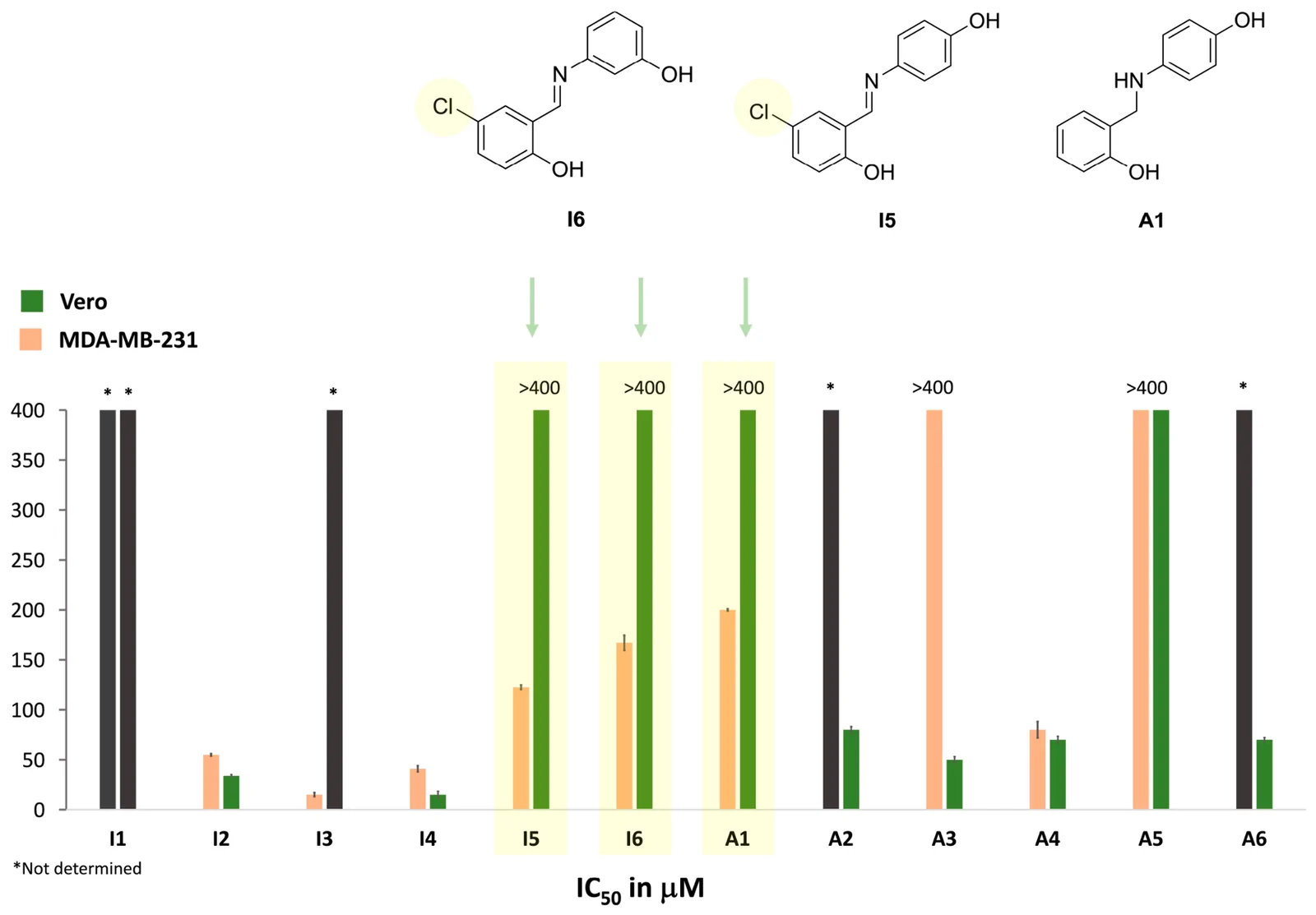
Antiproliferative activity of the synthesized imine (I1–I6) and amine (A1–A6) derivatives against human triple-negative breast cancer MDA-MB-231 cells and noncancerous Vero cells, as determined by the MTT assay. Data are expressed as IC_50_ values (μM) and represent the mean ± SD of at least three independent experiments.

The cytotoxicity data obtained from the MTT assay allowed the determination of the antiproliferative effects of the synthesized compounds, as well as their selectivity toward cancerous versus noncancerous cells. The results are presented in Figure 9 and Table S2 in the Supporting Information. Overall, the imine derivatives (I1–I6) exhibited stronger cytotoxic effects than the amine derivatives (A1–A6) toward both cell lines. Notably, compounds I2, I3, and I4 displayed pronounced cytotoxicity against MDA-MB-231 cells, with IC_50_ values of 54.9 ± 1.09 µM, 15.1 ± 1.99 µM, and 40.99 ± 2.94 µM, respectively. However, I2 and I4 also showed high toxicity toward normal Vero cells, with IC_50_ values of 33.9 ± 1.29 µM and 14.97 ± 3.45 µM, respectively. For compound I3, the IC_50_ toward Vero cells could not be determined due to a non-monotonic or biphasic dose–response curve, making its in vitro safety profile unreliable. Among all tested derivatives, the chloro-substituted imine compounds I5 and I6 demonstrated a more favorable selectivity profile (with lower-limit SI values of >3.26 and >2.39, respectively). Although they exhibited moderate cytotoxicity toward MDA-MB-231 cells (IC_50_ = 122.6 ± 2.23 μM for I5 and 167.1 ± 7.58 μM for I6), both compounds showed minimal toxicity toward Vero cells (IC_50_ > 400 μM). Similarly, the non-substituted amine derivative A1 displayed selective antiproliferative activity (SI > 2.00) against MDA-MB-231 cells (IC_50_ = 200 ± 1.09 μM) while exhibiting minimal cytotoxicity to Vero cells at the tested concentrations (IC_50_ > 400 μM). In contrast, several amine derivatives, including A2, A3, and A4, exhibited either limited activity against MDA-MB-231 cells or comparable cytotoxicity toward both cancerous and noncancerous cells, indicating reduced selectivity. Collectively, these results suggest that while imine derivatives generally confer higher cytotoxic potency, selected chloro-substituted imines and non-substituted amines provide a better balance between anticancer activity and lower cytotoxicity toward the non-tumor cell model. However, because Vero cells are of non-human kidney origin rather than a tissue-matched human mammary control, these findings represent preliminary in vitro selectivity rather than absolute safety. Subsequent evaluations utilizing appropriate human somatic cell lines will be necessary to establish definitive safety profiles.

Overall, these findings are in good agreement with previously reported studies demonstrating that imine derivatives and their reduced amine analogs can exert potent cytotoxic activity against cancer cell lines, including the triple-negative breast cancer MDA-MB-231 cells, highlighting the relevance of this chemical class for anticancer drug discovery [64,65].

### 3.5. Docking Studies

To explore hypothetical molecular targets that might contribute to the observed anticancer activity, an exploratory molecular docking study was performed against selected cancer-relevant targets using derivative I5, which was selected due to its optimal balance of moderate cytotoxicity against breast cancer cells and high selectivity over normal cells. EGFR, tubulin, and Topoisomerase II were chosen based on previously reported studies demonstrating that imine (Schiff base) derivatives and their analogs can exert antiproliferative effects through modulation of these targets in breast cancer cells, including MDA-MB-231 [66,67,68,69,70]. EGFR plays a key role in the proliferation and survival of triple- negative breast cancer cells, and several imine-based compounds have been reported to interact with the EGFR kinase domain [67]. Tubulin is a well-established anticancer target, and numerous imine derivatives have been shown to disrupt microtubule dynamics, leading to cell-cycle arrest and apoptosis [67,68,69]. In addition, Topoisomerase II is a relevant nuclear target since imine-containing compounds have been reported to interfere with DNA topology and induce cancer cell death [70].

The binding affinities of the reference ligands and the investigated compound I5 toward the selected molecular targets are summarized in Table 3. For the EGFR kinase domain (PDB ID: 1M17), the reference inhibitor 4-anilinoquinazoline exhibited a binding energy of −7.42 kcal/mol, while I5 showed a slightly stronger interaction with a binding energy of −7.72 kcal/mol. This improvement suggests that I5 is capable of efficiently occupying the ATP-binding pocket of EGFR and may display comparable inhibitory potential relative to the known ligand. In the case of Tubulin at the colchicine-binding site (PDB ID: 1SA0), colchicine displayed a markedly strong binding affinity (−14.45 kcal/mol), reflecting its well-established high potency toward this target. Although I5 demonstrated a weaker interaction (−8.54 kcal/mol), the obtained value still indicates a favorable binding within the colchicine site, suggesting that I5 may partially mimic the binding mode of colchicine, albeit with reduced affinity. For human Topoisomerase IIβ (PDB ID: 3QX3), the reference drug etoposide showed a binding energy of −8.91 kcal/mol, whereas I5 exhibited a binding energy of −7.16 kcal/mol. While the interaction of I5 with Topo IIβ is weaker than that of etoposide, the negative binding energy nonetheless indicates a possible stable ligand–protein complex, supporting a potential inhibitory interaction.

**Figure 10:**
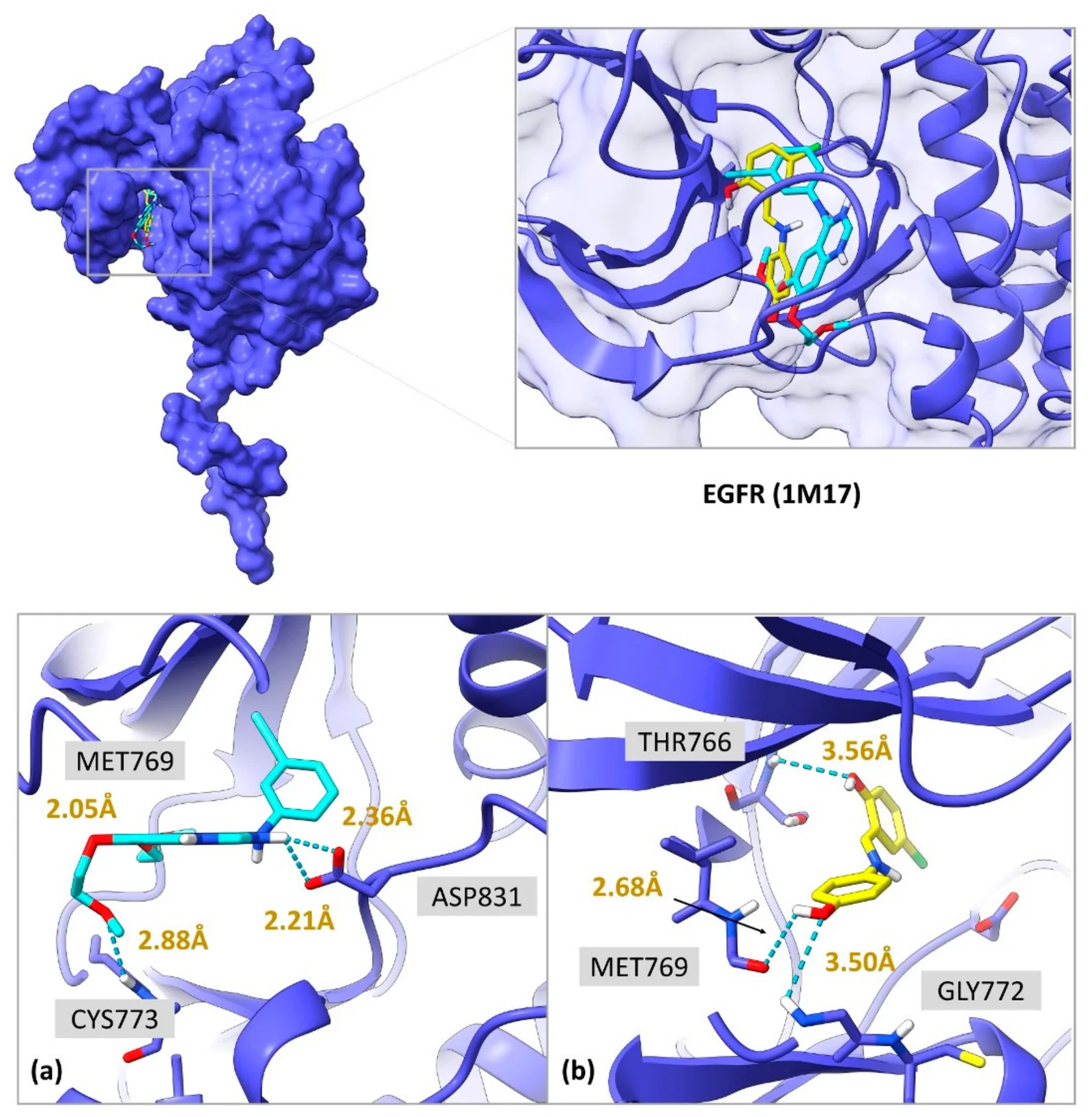
Molecular docking poses and binding interactions of (**a**) the reference inhibitor 4-anilinoquinazoline and (**b**) compound I5 within the ATP-binding site of the EGFR kinase domain (PDB ID: 1M17).

The docking analysis of the EGFR kinase domain, as shown in Figure 10, reveals that both the reference inhibitor and compound I5 bind within the ATP-binding pocket through a conserved hinge-region interaction. The reference ligand, 4-anilinoquinazoline, establishes a strong hydrogen bond with the hinge residue MET769 at a distance of 2.05 Å, which is a hallmark interaction for effective EGFR inhibition. In addition, it forms two hydrogen bonds with ASP821 (2.21 and 2.36 Å) and an additional interaction with CYS773 (2.88 Å), resulting in a dense hydrogen-bond network that contributes to its stable binding and favorable inhibitory profile. In comparison, compound I5 also engages the hinge region via a hydrogen bond with MET769 (2.68 Å), confirming its proper orientation within the active site. Furthermore, I5 forms supplementary interactions with THR766 (3.56 Å) and GLY772 (3.50 Å), which help stabilize the ligand within the binding cavity, although these interactions are slightly longer and fewer than those observed for the reference compound. Overall, while the reference inhibitor exhibits a more optimized interaction pattern, the ability of I5 to maintain the critical hinge interaction with MET769 supports its favorable docking score and suggests a plausible EGFR inhibitory potential.

**Figure 11:**
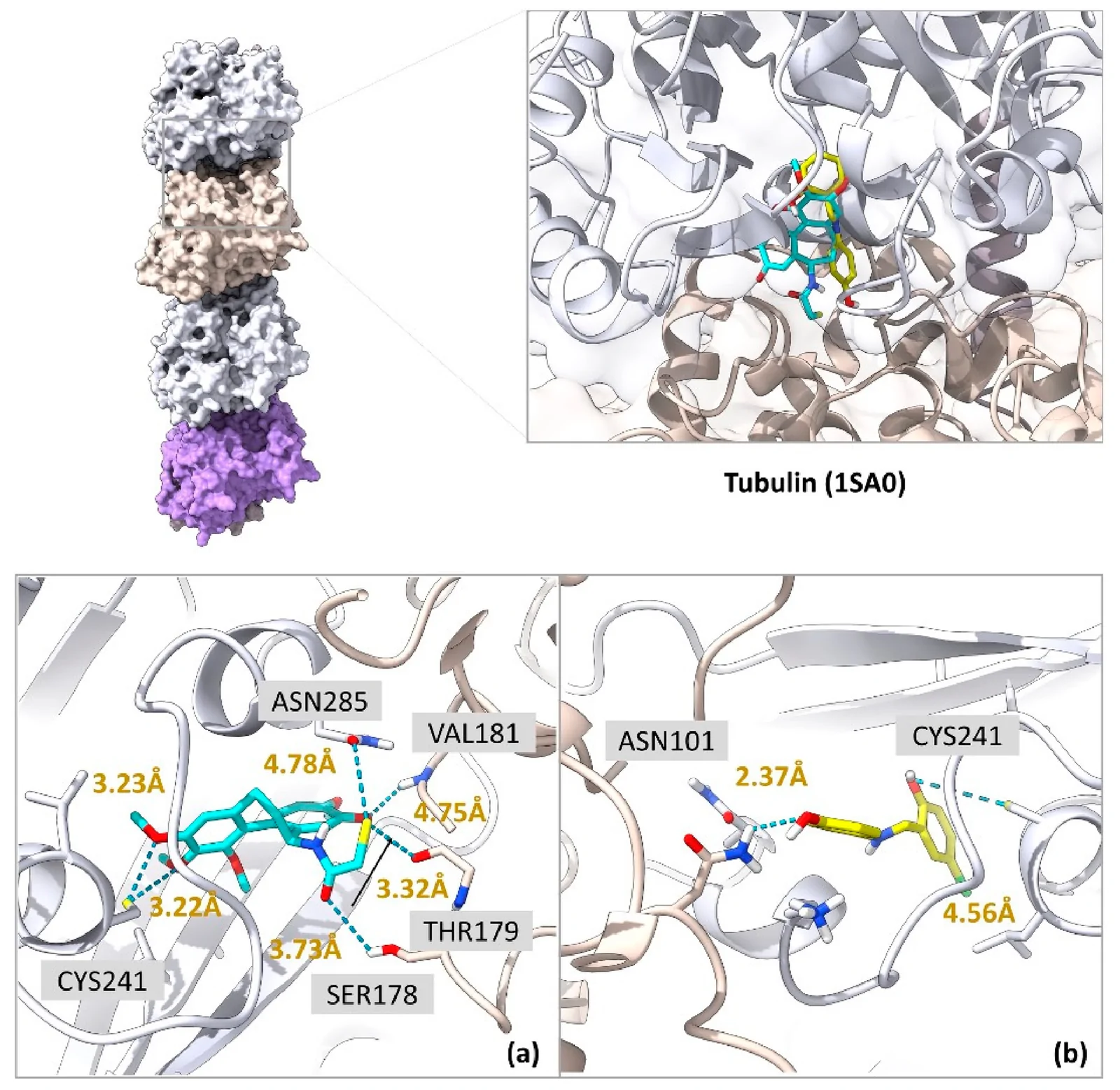
Molecular docking poses and binding interactions of (**a**) the reference ligand colchicine and (**b**) compound I5 within the colchicine-binding site of tubulin (PDB ID: 1SA0).

The docking analysis at the colchicine-binding site of tubulin highlights clear differences between the interaction patterns of the reference ligand colchicine and compound I5 (Figure 11). Colchicine displays an extensive and well-distributed network of stabilizing interactions with key residues lining the binding pocket, including CYS241, THR179, SER178, ASN285, and VAL181, with interaction distances ranging from approximately 3.2 to 4.8 Å. This dense interaction pattern is consistent with the strong binding affinity observed for colchicine and reflects its well-established ability to tightly occupy and stabilize the colchicine site, thereby effectively disrupting microtubule polymerization. In contrast, compound I5 exhibits a more limited interaction profile within the same binding cavity. The most prominent interaction is a strong hydrogen bond with ASN101 at a distance of 2.37 Å, indicating a well-defined anchoring point for the ligand. An additional, weaker contact with CYS241 (4.56 Å) suggests partial accommodation within the colchicine pocket but with reduced surface complementarity compared to colchicine. Overall, the reduced number and longer distances of interactions observed for I5 explain its lower binding affinity relative to the reference ligand. Nevertheless, the presence of a strong hydrogen bond and its correct positioning within the colchicine-binding site support the potential of I5 as a tubulin-interacting scaffold that could be further optimized to enhance binding strength and interaction coverage.

**Figure 12:**
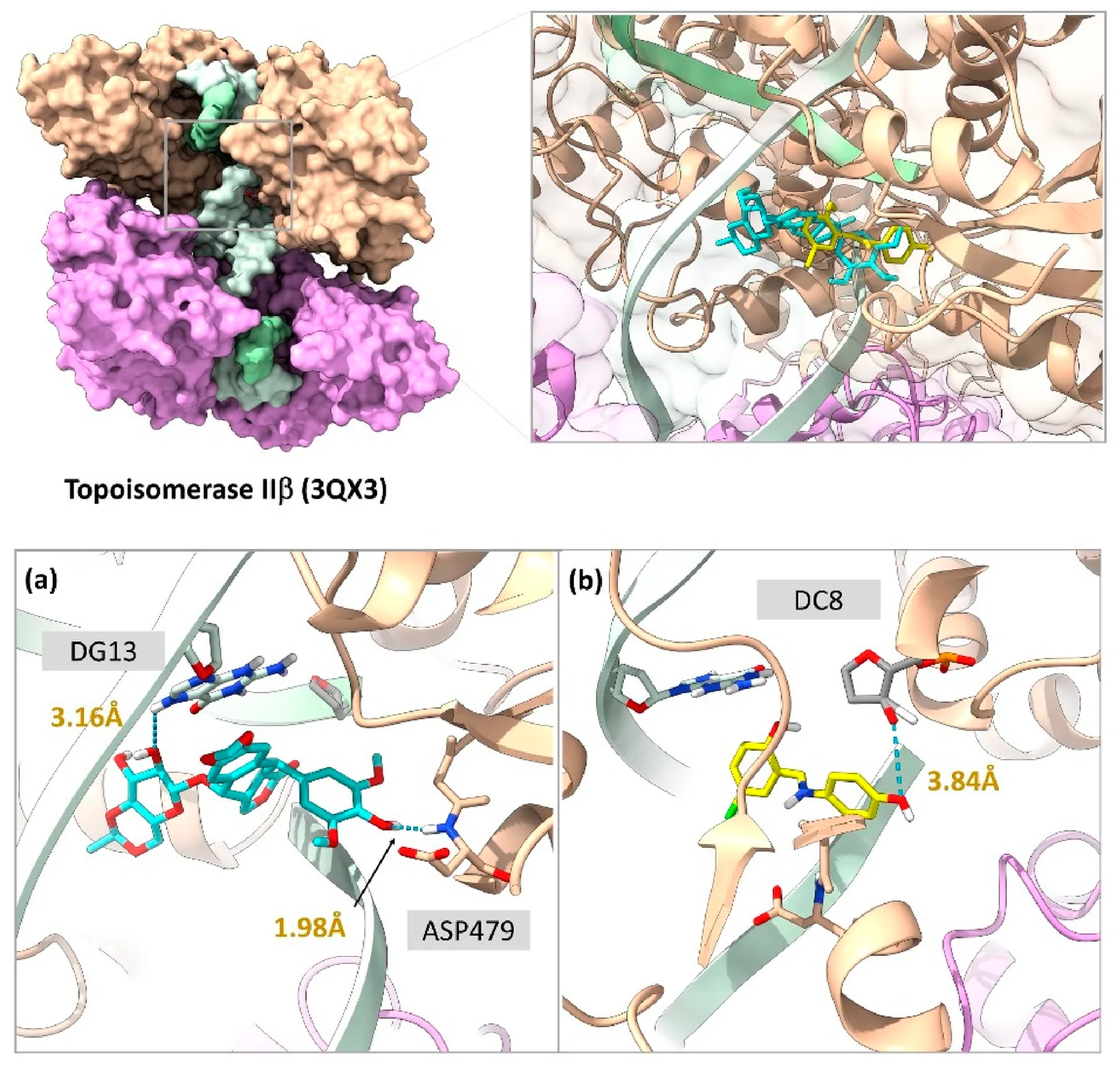
Molecular docking poses and binding interactions of (**a**) the reference inhibitor etoposide and (**b**) compound I5 within the DNA–enzyme cleavage complex of human Topoisomerase IIβ (PDB ID: 3QX3).

The docking analysis of human Topoisomerase Iiβ reveals distinct binding behaviors for the reference ligand etoposide and compound I5 within the DNA–enzyme cleavage complex (Figure 12). Etoposide exhibits a well-defined and stabilized binding mode, characterized by a strong hydrogen bond with the catalytic residue ASP479 at a short distance of 1.98 Å, along with an additional interaction with the DNA base DG13 at 3.16 Å. This dual interaction pattern, involving both the protein and the DNA component of the complex, is consistent with the known mechanism of action of etoposide as a Topoisomerase II poison, where stabilization of the cleavage complex leads to inhibition of DNA religation. In contrast, compound I5 shows a more limited interaction profile within the same binding region. The main observed interaction is with the DNA base DC8 at a distance of 3.84 Å, with no strong hydrogen bonds detected with key catalytic protein residues. This suggests that I5 may interact preferentially with the DNA component rather than directly targeting the active-site residues of the enzyme. Consequently, the reduced number and weaker nature of interactions observed for I5 provide a structural rationale for its lower binding affinity compared to etoposide. Overall, while I5 is capable of occupying the Topoisomerase IIβ binding region, further structural optimization would be required to enhance its interactions with critical protein residues and improve its inhibitory potential.

Overall, the molecular docking study provides an exploratory structural rationale for compound I5. The favorable binding affinities and conserved hinge-region interactions observed computationally demonstrate the theoretical compatibility of I5 within the EGFR kinase domain; however, these in silico findings do not constitute evidence of direct EGFR inhibition. Furthermore, while I5 demonstrated the capacity to occupy the binding sites of tubulin and Topoisomerase IIβ, these interactions similarly reflect theoretical compatibility rather than confirmed biological activity. Taken together, these exploratory computational results suggest a potential multitarget profile that warrants future experimental enzymatic validation to confirm the true mechanism of action, while justifying the continued optimization of this scaffold

### 3.6. Molecular Dynamics Simulations

Molecular dynamics simulations were performed for the EGFR, Tubulin and Topoisomerase IIβ systems, with compound I5 bound to each structure in the position proposed by their respective molecular docking studies. As a reference baseline, molecular dynamics simulations with their respective co-crystallized ligands (4-anilinoquinazoline for EGFR, colchicine for Tubulin and etoposide for Topoisomerase IIβ) were also performed. These simulations assessed the structural integrity of the systems and the interaction energies between the ligands and their respective binding sites over time.

#### 3.6.1. EGFR

For the EGFR-4-anilinoquinazoline system, the protein backbone’s RMSD remains stable at approximately 0.4 nm after 75 ns of simulation, indicating that its global structure remained stable. For the EGFR-I5 system, the protein backbone’s RMSD stabilizes at around 0.3 nm. EGFR’s radius of gyration also remains stable throughout the simulation, and secondary structure analysis shows that no major changes occur to EGFR in any of both simulations. Detailed structural analyses and trajectories are provided in the Supplementary Information (Figure S1).

**Figure 13:**
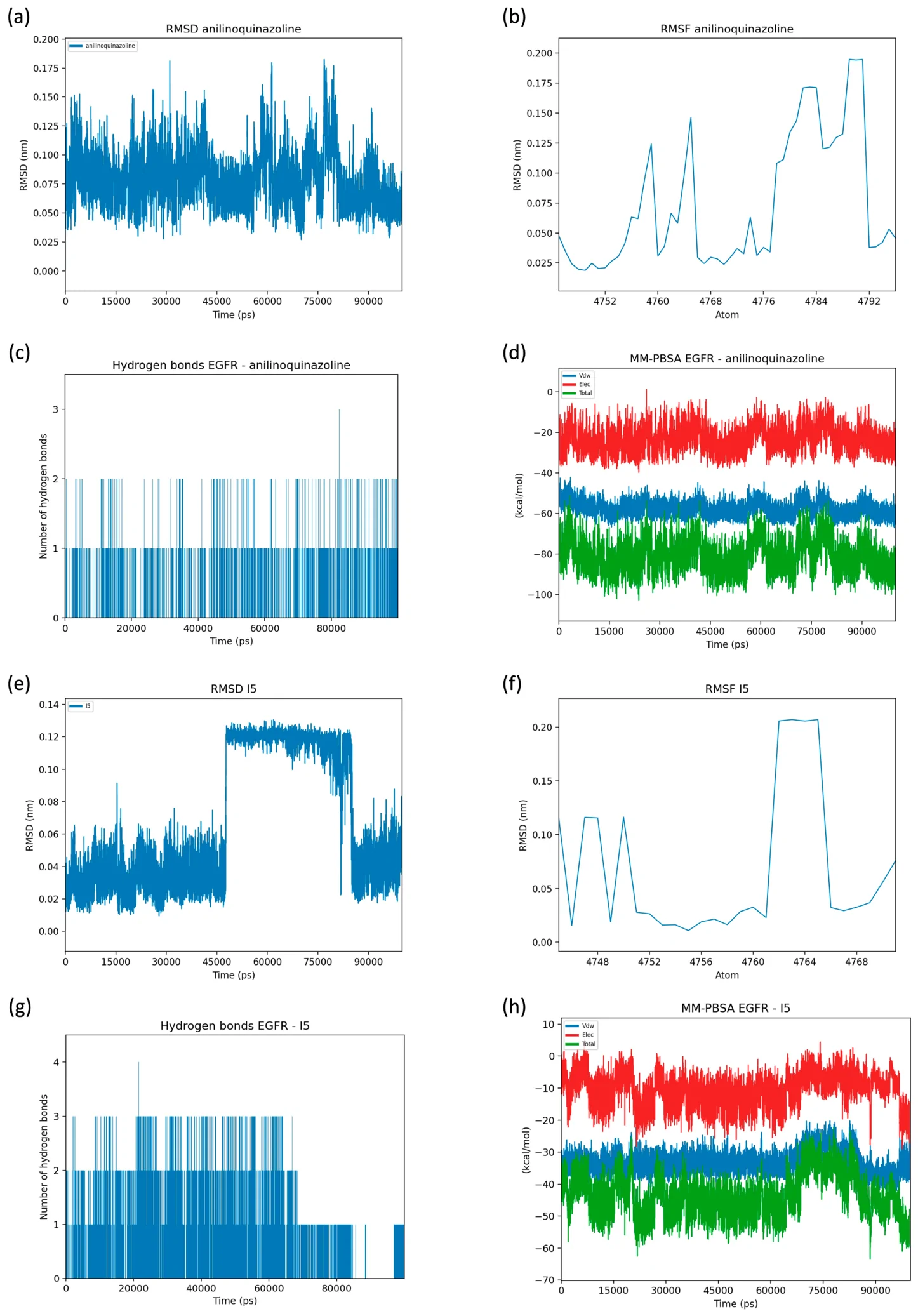
Molecular dynamics simulation analysis for EGFR ligand complexes. (**a**) Global RMSD of the reference ligand 4-anilinoquinazoline. (**b**) Per-atom RMSF of 4-anilinoquinazoline. (**c**) Number of hydrogen bonds established between 4-anilinoquinazoline and the EGFR binding site. (**d**) MM-PBSA analysis for 4-anilinoquinazoline, displaying the estimations of the electrostatic (red) and Van der Waals (blue) contributions to the total binding energy (green) during the 100 ns simulation. (**e**) Global RMSD of compound I5 during the simulation. (**f**) Per-atom ligand RMSF of compound I5. (**g**) Number of hydrogen bonds established between compound I5 and the EGFR binding site. (**h**) MM-PBSA analysis for compound I5, displaying the estimations of the electrostatic (red) and Van der Waals (blue) contributions to the total binding energy (green) during the 100 ns simulation.

The reference ligand exhibited a stable RMSD of approximately 0.1 nm inside the binding pocket during the simulation. Figure 13a,b illustrates the global and per-atom RMSD of the reference ligand, showing the especially stable RMSD of the 4-anilinoquinazoline scaffold atoms, with RMSD < 0.75 nm. Figure 13b,c, on the other hand, shows the EGFR-reference ligand interaction details. 4-anilinoquinazoline consistently establishes between 1 and 2 hydrogen bonds throughout the entire simulation. An estimate of the protein–ligand binding free energy was obtained through Molecular Mechanics Poisson–Boltzmann Surface Area (MMPBSA) analysis. As expected for a reference EGFR inhibitor, it showed a high total binding energy of around −84 kcal/mol.

A deeper, per-residue analysis registered the interaction energy of the ligand with its surrounding residues. Table 4 summarizes the interaction energies, showing notable contributions from relevant residues already noticed in the docking studies, such as the hinge residue MET769 (−3.2 ± 1.3 kcal/mol), ASP831 (−2.2 ± 0.6 kcal/mol), CYS773 (−6.8 ± 2.4 kcal/mol) and THR766 (−3.8 ± 0.9 kcal/mol). Other residues such as ALA719, Gly772, Leu694, Leu820, Met769, Thr830 or Val702 also contribute notably to the stabilization of the reference drug inside the pocket.

Compound I5 also shows high stability inside the binding pocket throughout the entire simulation. Figure 13e,f shows its RMSD inside the binding pocket, which presents a stable plateau of approximately 0.12 nm from 45 ns to 80 ns of simulation. Per-atom RMSF shows that the RMSD contribution primarily comes from the ortho and meta-atoms of the p-hydroxyphenyl ring, probably due to certain rotational freedom of the aromatic ring plane. The I5 compound’s interactions with the EGFR kinase domain are displayed in Figure 13g,h. Compound I5 consistently maintains 2 to 3 hydrogen bonds until the last 30 ns of simulation, when they decrease to 1 or none. MM-PBSA analysis estimates a total binding energy of −44 ± 6 kcal/mol, with a transient weakening to −39 ± 7 kcal/mol between 60 and 80 ns that follows the decrease in hydrogen bond interactions, although it recovers to −45 ± 7 kcal/mol over the last 20 ns.

A summary of the most notable per-residue interactions is shown in Table 5. Similar to the reference drug, compound I5 also interacts with key residues such as the hinge residue MET769 (−1.7 ± 1.8 kcal/mol), THR766 (−4.6 ± 1.8 kcal/mol), ASP831 (−2.3 ± 3.0 kcal/mol), as well as notable interactions with ALA719 (−2.3 ± 0.8 kcal/mol), LEU764 (−5.8 ± 2.1 kcal/mol) and LYS721 (−3.7 ± 1.0 kcal/mol).

#### 3.6.2. Tubulin

During both of the Tubulin molecular dynamics simulations, with the I5 compound and its own reference ligand, colchicine, the protein structure stabilizes its RMSD at around 0.4 nm and 0.5 nm, respectively. The radius of gyration in both simulations was also regular, around 5.25 and 5.35 nm in the simulation with the I5 compound, and slightly longer, around 5.35 and 5.45, in the reference ligand simulation. For any simulation, no changes are observed in Tubulin’s secondary structure. Detailed structural analyses and trajectories are provided in the Supplementary Information (Figure S2).

**Figure 14:**
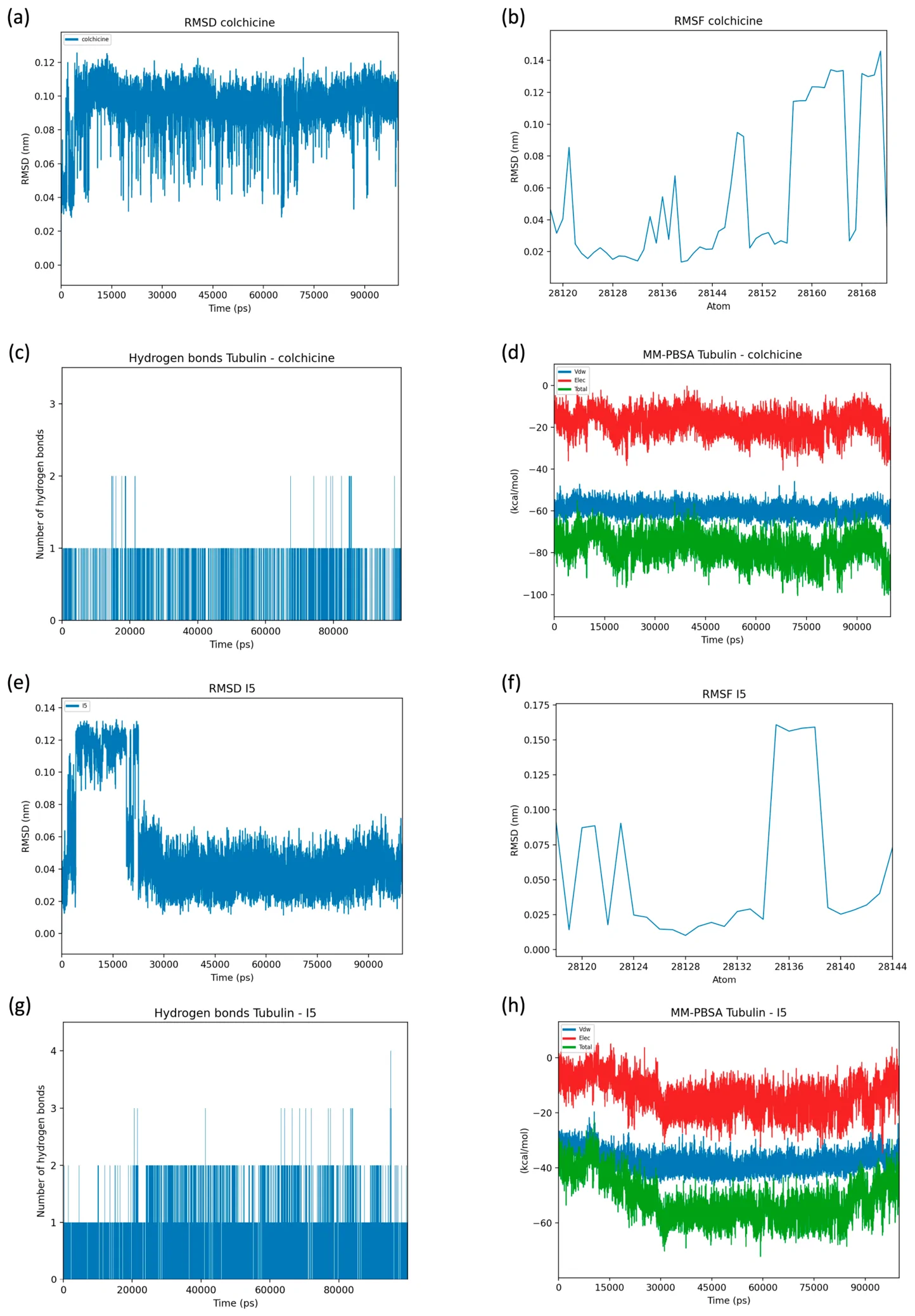
Molecular dynamics simulation analysis for Tubulin ligand complexes. (**a**) Global RMSD of colchicine during the simulation. (**b**) Per-atom ligand RMSF of colchicine. (**c**) Number of hydrogen bonds established between colchicine and the Tubulin binding site. (**d**) MM-PBSA analysis for colchicine, displaying the estimations of the electrostatic (red) and Van der Waals (blue) contributions to the total binding energy (green) during the 100 ns simulation. (**e**) Global RMSD of compound I5 during the simulation. (**f**) Per-atom ligand RMSF of compound I5. (**g**) Number of hydrogen bonds established between compound I5 and the Tubulin binding site. (**h**) MM-PBSA analysis for compound I5, displaying the estimations of the electrostatic (red) and Van der Waals (blue) contributions to the total binding energy (green) during the 100 ns simulation.

The reference ligand, colchicine, remained inside the binding pocket throughout the simulation, with a stable RMSD of approximately 0.09 nm, to which the main contributions are hydrogen atoms, as shown in Figure 14a,b. Colchicine established one hydrogen bond intermittently with Tubulin, and MM-PBSA analysis estimates a total binding energy range of approximately −72 to −84 kcal/mol (Figure 14c,d).

Per-residue interactions are shown in Table 6. During de molecular dynamics simulation, colchicine interacts with key residues already identified through molecular docking: CYS241 (−2.4 ± 1.6 kcal/mol), THR179 (−0.8 ± 0.5 kcal/mol), SER178 (−0.4 ± 0.9 kcal/mol), ASN258 (−3.8 ± 1.0 kcal/mol) and VAL181 (−4.1 ± 1.2 kcal/mol), along with other significative contributions from ALA180 (−3.8 ± 0.7 kcal/mol), ALA316 (−2.4 ± 0.7 kcal/mol), LEU248 (−5.4 ± 1.0 kcal/mol), LEU255 (−5.8 ± 0.8 kcal/mol) and LYS352 (−10.6 ± 2.1 kcal/mol) residues.

During the Tubulin-I5 simulation, the I5 compound remained inside the binding pocket with an RMSD value stable at around 0.04 nm after 30 ns of simulation, as shown in Figure 14e,f. The per-atom RMSF plot reveals an identical RMSD fluctuation profile of I5 to that of the same compound during EGFR simulation, with the rotation of the p-hydroxyphenyl ring the main contributor to the ligand RMSD. Figure 14g,h shows that compound I5 consistently maintains between one and two hydrogen bonds, especially after the first 20 ns of simulation. After that time, the MM-PBSA total energy prediction lowers from an oscillation between −36 and −48 kcal/mol to a much more stable one at around −57 kcal/mol. The increase in hydrogen bonds and total energy lowering from the MM-PBSA analysis after the first 20 ns coincides in time with the adoption of a pose with very low RMSD, implying that the I5 compound adopts a more energetically favorable state when its pose resembles that from the molecular docking.

Per-residue I5 interactions with the Tubulin binding site during the molecular dynamics simulation are summarized in Table 7. Indeed, CYS241, a residue already noticed in molecular docking studies, contributes to the stabilization of the ligand (−2.0 ± 0.6 kcal/mol), but no interaction has been registered with the promising residue ASN101. However, many other strong interactions can be seen with ALA317 (−6.3 ± 1.3 kcal/mol), ALA250 (−2.3 ± 0.8 kcal/mol) ASP251 (−2.8 ± 1.1 kcal/mol), LEU255 (−4.3 ± 0.7 kcal/mol), LYS254 (−3.8 ± 1.5 kcal/mol), LYS352(−4.4 ± 1.7 kcal/mol), SER178 (−4.3 ± 3.6 kcal/mol) and VAL318 (−2.1 ± 0.5 kcal/mol) residues, as well as other minor interactions with residues that take an important role on Tubulin–colchicine binding, such as THR179, ASN258, ALA180, ALA316 and LEU248.

#### 3.6.3. Topoisomerase IIβ

During the simulation of both Topoisomerase IIβ systems, not only the protein but also the DNA structural integrity was monitored. For Topoisomerase IIβ in complex with the reference ligand etoposide, protein RMSD stabilized at around 0.6 nm, and DNA at 0.3 nm. For the Topoisomerase IIβ—I5 system, RMSD values for the protein and DNA were slightly lower, being approximately 0.4 and 0.25 nm, respectively. The radius of gyration of the protein in complex with etoposide was around 3.95 and 4.00 nm, while that of the protein in complex with the I5 compound was again slightly lower, around 3.90 and 3.85 nm. While the secondary structure in the I5 simulation remained stable, the secondary structure in the reference simulation started slightly differently in comparison, decreasing in the number of amino acids interpreted as forming β-sheets and turns, and increasing in the number of amino acids interpreted as forming alpha-helices and coils. As for the DNA structure integrity in etoposide and I5 simulations, RMSD stabilizes at around 0.35 and 0.25 nm, respectively. Furthermore, DNA establishes between two and four hydrogen bonds throughout the reference ligand simulation, and more consistently in the I5 simulation, where up to 5 and 6 hydrogen bonds are frequently established. Detailed structural analyses and trajectories are provided in the Supplementary Information (Figure S3).

**Figure 15:**
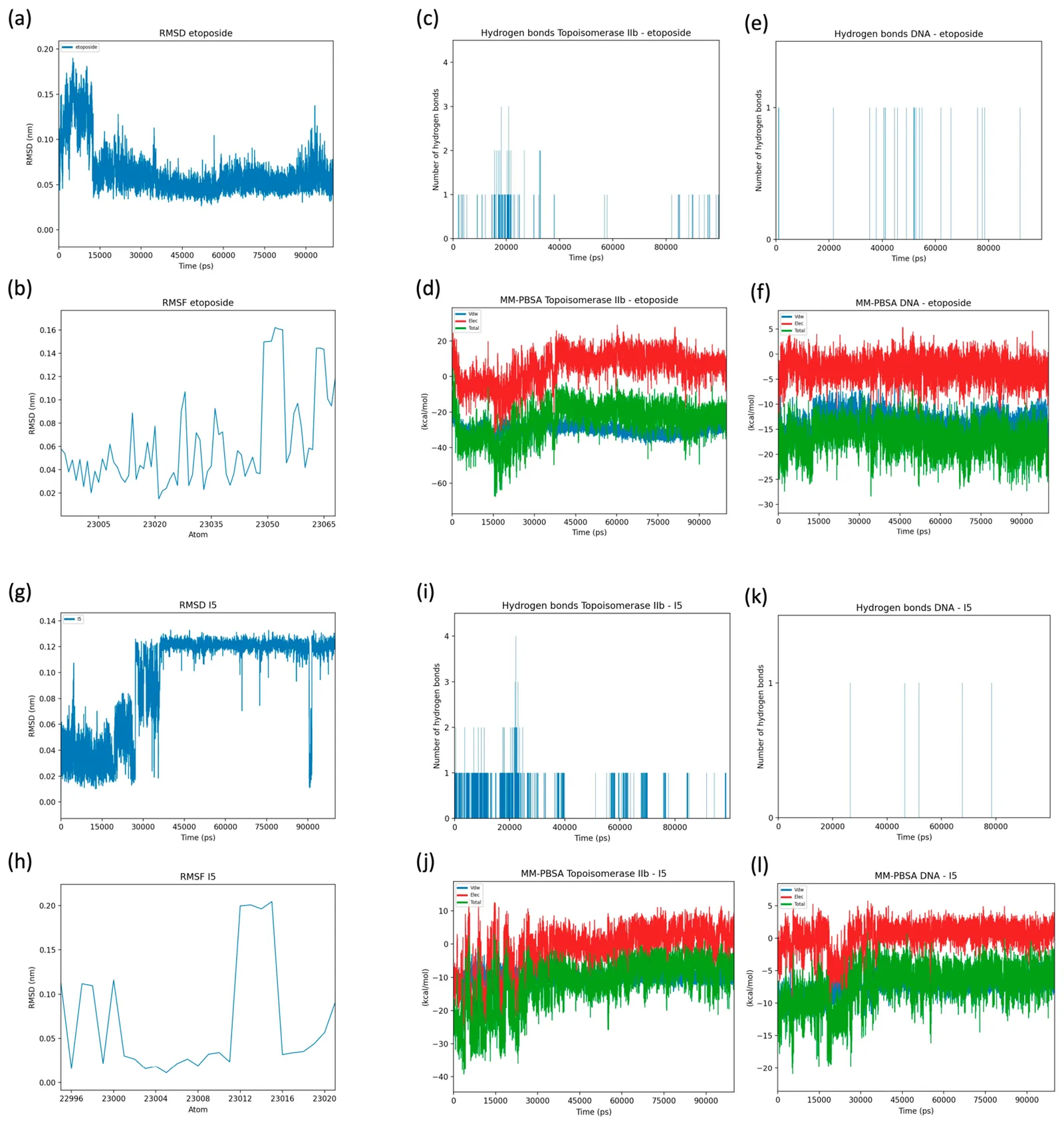
Molecular dynamics simulation analysis for Topoisomerase IIβ ligand complexes. (**a**) Global RMSD of etoposide during the simulation. (**b**) Per-atom ligand RMSF of etoposide. (**c**) Number of hydrogen bonds established between etoposide and the protein inside the Topoisomerase IIβ binding site. (**d**) Number of hydrogen bonds established between etoposide and DNA inside the Topoisomerase IIβ binding site. (**e**,**f**) MM-PBSA analysis for etoposide, displaying the estimations of the electrostatic (red) and Van der Waals (blue) contributions to the total binding energy (green) during the 100 ns simulation, for the etoposide-protein and etoposide-DNA binding, respectively. (**g**) Global RMSD of compound I5 during the simulation. (**h**) Per-atom ligand RMSF of compound I5. (**i**) Number of hydrogen bonds established between compound I5 and the protein inside the Topoisomerase IIβ binding site. (**j**) Number of hydrogen bonds established between compound I5 and the DNA portion. (**k**,**l**) MM-PBSA analysis for compound I5, displaying the estimations of the electrostatic (red) and Van der Waals (blue) contributions to the total binding energy (green) during the 100 ns simulation, for the I5-protein and I5-DNA binding, respectively.

The reference ligand etoposide remained within the Topoisomerase Iiβ-binding pocket during the whole simulation. In Figure 15a,b, it can be seen that the ligand has a low and stable RMSD of approximately 0.05 nm after 15 ns. Hydrogen bonds and the estimated binding energy of the reference ligand etoposide with the protein and DNA structures are shown in Figure 15c–f. With the protein structure, etoposide establishes up to two or three hydrogen bonds at around 20 ns of simulation. At that same time, MM-PBSA estimates a binding energy minimum of approximately −48 to −60 kcal/mol. During the remaining simulation time, hydrogen bonds are scarce and, as a result, the electrostatic contribution becomes unfavorable, and the total estimated binding energy ascends up to around −24 kcal/mol, supported only by Van der Waals interactions. Etoposide interactions with the DNA strands, as expected, appear to be complementary to those with the protein, displaying hydrogen bonds more frequently after the first 40 ns of simulation, after the interactions with the protein weaken. MM-PBSA binding energy estimations for the etoposide-DNA binding oscillate around −17 kcal/mol.

A detailed view of the main interactions that etoposide establishes with the Topoisomerase IIβ /DNA system can be seen in Table 8. No interaction with the catalytic ASP479 residue has been registered. Instead, the main interactions with the protein during the 100 ns simulation take place with the ARG820 (−2.7 ± 1.9 kcal/mol), GLN778 (−3.2 ± 1.0 kcal/mol), MET781 (−4.8 ± 1.7 kcal/mol) and MET782 (−6.0 ± 1.2 kcal/mol) residues from chain B. Etoposide also interacts with close nucleotides DC8 (−6.1 ± 2.7 kcal/mol) and DG10 (−2.7 ± 1.0 kcal/mol) from the DNA strand.

On the other hand, the I5 compound also maintained its position inside the binding pocket, giving its low RMSD, shown in Figure 15g,h. After 40 ns of simulation, its RMSD stabilizes at approximately 0.12 nm. Once again, its main pose change comes from the rotation of the aforementioned aromatic ring. As for the I5 interactions with the Topoisomerase IIβ/DNA system, Figure 15i–l shows that the main contributions come from the protein residues. During the first 40 ns, I5 consistently establishes between 1 and 2 hydrogen bonds with the protein. After that time, they become scarce, and the electrostatic contribution to the binding is practically zero, lowering the total estimated binding energy from −24 kcal/mol to −10 kcal/mol, approximately. With DNA, almost no hydrogen bond has been established throughout the entire simulation, and as a result, the electrostatic contribution to the total energy (approximately −7 kcal/mol), except for a brief period around 20 ns, is practically zero.

The main interactions of I5 with the protein residues and DNA strands is shown in Table 9. The main residue contributions to the stabilization of I5 come from ARG503 (−3.6 ± 0.9 kcal/mol) and GLN778 (−4.2 ± 2.4 kcal/mol). Again, no interactions occur with the catalytic ASP479, but it does establish strong interactions with nucleotide DC8 (−11.5 ± 8.1 kcal/mol).

## 4. Conclusions

In conclusion, two related series of salicylaldehyde-derived phenolic derivatives incorporating either an imine (I1–I6) or a secondary amine (A2–A6) linkage were successfully synthesized in good to excellent yields. In vitro antioxidant assays demonstrated that the activity of these compounds strongly depends on their structural features and the specific assay conditions. The imine derivatives exhibited superior performance in the ABTS and phenanthroline assays, whereas the amine analogs were generally more active in the DPPH test. Among all tested compounds, A6 showed the highest antioxidant efficiency, surpassing the reference standards BHT and BHA. DFT calculations performed in aqueous medium provided mechanistic insight into the observed trends. The results indicate that antioxidant behavior is primarily governed by the phenolic hydroxyl groups and is highly mechanism-dependent. The salicylaldehyde moiety represents the most favorable deprotonation site, facilitating the SPLET pathway under polar conditions, while hydrogen atom transfer is mainly associated with the aniline hydroxyl group. Although HAT is thermodynamically feasible, kinetic analysis revealed that SPLET is the dominant mechanism for A6 in aqueous solution. In addition to antioxidant activity, the chloro-substituted derivatives (I5, I6, and A6) displayed moderate antiproliferative effects against MDA-MB-231 triple-negative breast cancer cells, with comparatively lower toxicity toward Vero cells, indicating a degree of selectivity. Molecular docking studies of I5 suggested favorable interactions with key cancer-related targets, including EGFR, Tubulin, and Topoisomerase II, while 100 ns molecular dynamics simulations confirmed the long-term structural stability and sustained binding free energies of these theoretical complexes. These computational findings serve as an exploratory hypothesis for potential multimodal activity, which will require direct experimental validation through targeted biological assays. The findings of this study identify salicylaldehyde-derived imine and amine derivatives as accessible multifunctional scaffolds with possible antioxidant and anticancer properties. The mechanistic insights obtained herein provide a rational basis for further structural optimization aimed at enhancing biological performance.

## Figures and Tables

**Table 1:** Atoms in Molecules (AIM) descriptors for ring critical points (RCP, type (3, +1)) and bond critical points (BCP, type (3, −1)) associated with intramolecular interactions in A1–A6 and I1–I6 derivatives.

Comp.	CP Type	ρ(r) (a.u.)	∇^2^ρ(r) (a.u.)	G(r) (a.u.)	V(r) (a.u.)	H(r) (a.u.)	Ellipticity
A1	RCP	0.01053	0.05202	0.01084	−0.00867	0.00217	−1.472
	BCP	0.01126	0.04386	0.0096	−0.00824	0.00136	0.43
A2	RCP	0.01044	0.05153	0.01072	−0.00857	0.00216	−1.504
	BCP	0.01104	0.04348	0.00949	−0.0081	0.00139	0.489
A3	RCP	0.01037	0.05137	0.01069	−0.00854	0.00215	−1.456
	BCP	0.01117	0.04335	0.00948	−0.00813	0.00135	0.389
A4	RCP	0.01081	0.05563	0.01149	−0.00907	0.00242	−1.39
	BCP	0.01217	0.04782	0.01037	−0.00879	0.00158	0.313
A5	RCP	0.01041	0.05143	0.01071	−0.00856	0.00215	−1.471
	BCP	0.0112	0.0435	0.00951	−0.00816	0.00136	0.404
A6	RCP	0.01039	0.05124	0.01066	−0.00851	0.00215	−1.512
	BCP	0.01098	0.04331	0.00944	−0.00805	0.00139	0.49
I1	RCP	0.01758	0.11137	0.02298	−0.01812	0.00486	−1.265
	BCP	0.04411	0.11666	0.03508	−0.04099	−0.00591	0.036
I2	RCP	0.0177	0.11228	0.02318	−0.01829	0.00489	−1.265
	BCP	0.04502	0.11685	0.03563	−0.04205	−0.00642	0.035
I3	RCP	0.0176	0.11147	0.023	−0.01814	0.00486	−1.264
	BCP	0.04437	0.11604	0.0351	−0.04119	−0.00609	0.035
I4	RCP	0.01754	0.11106	0.02291	−0.01805	0.00485	−1.264
	BCP	0.04388	0.11615	0.03485	−0.04067	−0.00581	0.035
I5	RCP	0.01779	0.11307	0.02335	−0.01843	0.00492	−1.261
	BCP	0.04579	0.11701	0.03612	−0.04298	−0.00686	0.034
I6	RCP	0.01761	0.11163	0.02303	−0.01816	0.00488	−1.263
	BCP	0.04439	0.11661	0.03523	−0.04131	−0.00608	0.035

**Table 2:** HOMO and LUMO energies and global reactivity descriptors of the amine (A1–A6) and imine (I1–I6) derivatives, including ionization potential (I), electron affinity (A), chemical hardness (η), chemical potential (μ), electronegativity (χ), global softness (σ), electrophilicity index (ω), and nucleophilicity index (ξ), calculated at the M06-2X/6-311++G(d,p) level of theory.

Comp.	HOMO (eV)	LUMO (eV)	Gap (eV)	I (eV)	A (eV)	η (eV)	μ (eV)	χ (eV)	σ (eV^−1^)	ω (eV)	ξ
A1	−6.48	−0.32	6.16	6.48	0.32	3.08	−3.40	3.40	0.32	1.87	0.53
A2	−6.72	−0.36	6.36	6.72	0.36	3.18	−3.54	3.54	0.31	1.98	0.51
A3	−6.59	−0.46	6.13	6.59	0.46	3.07	−3.53	3.53	0.33	2.03	0.49
A4	−6.85	−0.50	6.35	6.85	0.50	3.18	−3.67	3.67	0.31	2.13	0.47
A5	−6.59	−0.45	6.15	6.59	0.45	3.07	−3.52	3.52	0.33	2.02	0.50
A6	−6.84	−0.48	6.36	6.84	0.48	3.18	−3.66	3.66	0.31	2.11	0.47
I1	−7.26	−1.04	6.22	7.26	1.04	3.11	−4.15	4.15	0.32	2.77	0.36
I2	−7.50	−1.18	6.33	7.50	1.18	3.16	−4.34	4.34	0.32	2.98	0.34
I3	−7.37	−1.29	6.08	7.37	1.29	3.04	−4.33	4.33	0.33	3.08	0.32
I4	−7.58	−1.42	6.17	7.58	1.42	3.08	−4.50	4.50	0.32	3.28	0.30
I5	−7.36	−1.29	6.07	7.36	1.29	3.04	−4.33	4.33	0.33	3.08	0.32
I6	−7.59	−1.40	6.19	7.59	1.40	3.10	−4.50	4.50	0.32	3.27	0.31

**Table 3:** Molecular docking binding energies (kcal/mol) of the reference ligands and compound I5 against EGFR kinase domain (PDB ID: 1M17), Tubulin colchicine-binding site (PDB ID: 1SA0), and human Topoisomerase IIβ (PDB ID: 3QX3).

Target	Ligand	Binding Energy in kcal/mol
EGFR (1M17)	4-anilinoquinazoline	−7.42
	I5	−7.72
Tubulin (1SA0)	Colchicine	−14.45
	I5	−8.54
Topo IIβ (3QX3)	Etoposide	−8.91
	I5	−7.16

**Table 4:** Interaction energies (kcal/mol) between EGFR and anilinoquinazoline. Values are averages over the production trajectory (mean ± standard deviation); the last column reports the value in the final frame of the trajectory. The Total row is the sum over all the entries of the table.

Residue	Lennard-Jones (kcal/mol)	Coulomb (kcal/mol)	Total (kcal/mol)	Last Step (kcal/mol)
ALA719	−2.5 ± 0.5	−0.4 ± 0.4	−3.0 ± 0.5	−2.9
ARG817	−1.6 ± 0.8	−0.5 ± 0.6	−2.1 ± 1.1	−2.9
ASN818	−0.6 ± 0.2	−0.4 ± 0.2	−0.9 ± 0.4	−1.5
ASP776	−1.1 ± 0.6	−0.6 ± 0.5	−1.7 ± 1.0	−1.1
ASP831	−1.3 ± 0.6	−0.9 ± 0.6	−2.2 ± 0.6	−1.7
CYS773	−4.0 ± 1.1	−2.8 ± 1.7	−6.8 ± 2.4	−9.1
GLN767	−0.4 ± 0.4	−1.9 ± 0.6	−2.3 ± 0.5	−2.4
GLU738	−0.1 ± 0.1	−0.2 ± 0.1	−0.3 ± 0.2	−0.2
GLY695	−1.2 ± 0.6	−0.3 ± 0.6	−1.6 ± 1.0	−2.7
GLY772	−2.1 ± 0.6	−1.4 ± 0.7	−3.6 ± 0.9	−3.5
ILE720	−1.7 ± 0.4	−0.0 ± 0.2	−1.8 ± 0.5	−1.5
ILE765	−0.9 ± 0.3	0.2 ± 0.3	−0.7 ± 0.4	−1.1
LEU694	−5.0 ± 0.8	−0.8 ± 0.5	−5.7 ± 1.0	−5.7
LEU764	−0.8 ± 0.8	−0.6 ± 0.5	−1.4 ± 0.8	−2.4
LEU768	−2.0 ± 0.6	−0.1 ± 0.2	−2.1 ± 0.7	−1.4
LEU775	−0.3 ± 0.3	0.0 ± 0.0	−0.3 ± 0.3	−0.1
LEU820	−3.8 ± 0.6	−0.1 ± 0.1	−3.9 ± 0.6	−4.4
LYS721	−3.0 ± 0.5	0.7 ± 0.3	−2.3 ± 0.6	−2.4
MET742	−0.9 ± 0.4	−0.0 ± 0.1	−1.0 ± 0.4	−1.2
MET769	−2.3 ± 0.7	−0.9 ± 0.8	−3.2 ± 1.3	−2.4
PHE699	−1.1 ± 0.6	−0.1 ± 0.3	−1.1 ± 0.7	−2.3
PHE771	−0.5 ± 0.4	−0.1 ± 0.2	−0.6 ± 0.5	−0.5
PRO770	−0.3 ± 0.3	−0.0 ± 0.2	−0.3 ± 0.4	−0.1
SER696	−0.5 ± 0.4	−0.1 ± 0.2	−0.6 ± 0.5	−1.2
THR766	−2.6 ± 0.7	−1.2 ± 0.7	−3.8 ± 0.9	−4.8
THR830	−2.1 ± 0.4	−0.2 ± 0.1	−2.4 ± 0.5	−2.2
VAL702	−3.1 ± 0.6	0.0 ± 0.1	−3.1 ± 0.6	−4.6
Total	−46.2 ± 14.2	−12.7 ± 10.9	−58.9 ± 19.4	−66.5

**Table 5:** Interaction energies (kcal/mol) between EGFR and I5. Values are averages over the production trajectory (mean ± standard deviation); the last column reports the value in the final frame of the trajectory. The Total row is the sum over all the entries of the table.

Residue	Lennard-Jones (kcal/mol)	Coulomb (kcal/mol)	Total (kcal/mol)	Last Step (kcal/mol)
ALA719	−2.1 ± 0.6	−0.2 ± 0.8	−2.3 ± 0.8	−1.2
ASP831	−1.5 ± 1.2	−0.8 ± 2.7	−2.3 ± 3.0	−16.6
CYS751	−0.3 ± 0.3	−0.0 ± 0.1	−0.3 ± 0.3	−0.5
GLN767	−0.4 ± 0.3	−0.4 ± 0.5	−0.8 ± 0.7	−0.0
GLY772	−0.4 ± 0.3	0.1 ± 0.3	−0.3 ± 0.4	−0.0
ILE720	−1.3 ± 0.3	0.5 ± 0.4	−0.9 ± 0.5	−1.9
ILE765	−0.9 ± 0.3	−0.2 ± 0.3	−1.2 ± 0.6	−0.4
LEU694	−1.2 ± 0.6	0.0 ± 0.2	−1.2 ± 0.7	−0.1
LEU753	−0.8 ± 0.7	0.1 ± 0.1	−0.7 ± 0.6	−1.7
LEU764	−0.9 ± 1.2	−4.9 ± 2.8	−5.8 ± 2.1	−2.9
LEU768	−1.0 ± 0.7	−0.1 ± 0.4	−1.0 ± 0.9	−0.0
LEU820	−1.6 ± 1.1	−0.1 ± 0.1	−1.7 ± 1.2	−0.0
LYS721	−4.1 ± 0.7	0.4 ± 0.6	−3.7 ± 1.0	−3.3
MET742	−1.4 ± 0.5	−0.2 ± 0.2	−1.6 ± 0.6	−0.3
MET769	−1.0 ± 0.9	−0.6 ± 1.4	−1.7 ± 1.8	−0.0
PHE699	−0.2 ± 0.4	−0.0 ± 0.1	−0.2 ± 0.5	−0.1
PHE832	−0.9 ± 1.1	−0.1 ± 0.2	−1.0 ± 1.2	−3.3
THR766	−2.7 ± 0.8	−1.9 ± 1.8	−4.6 ± 1.8	−2.9
THR830	−1.3 ± 0.5	0.0 ± 0.2	−1.3 ± 0.6	−1.4
VAL702	−1.8 ± 0.6	0.0 ± 0.1	−1.8 ± 0.7	−1.7
Total	−25.7 ± 13.3	−8.5 ± 13.3	−34.1 ± 20.1	−38.4

**Table 6:** Interaction energies (kcal/mol) between Tubulin and colchicine. Values are averages over the production trajectory (mean ± standard deviation); the last column reports the value in the final frame of the trajectory. The Total row is the sum over all the entries of the table.

Residue	Lennard-Jones (kcal/mol)	Coulomb (kcal/mol)	Total (kcal/mol)	Last Step (kcal/mol)
ALA180B	−2.0 ± 0.7	−1.8 ± 0.5	−3.8 ± 0.7	−3.6
ALA316B	−2.2 ± 0.7	−0.3 ± 0.3	−2.4 ± 0.7	−2.5
ALA317B	−1.5 ± 0.5	−0.5 ± 0.3	−2.0 ± 0.6	−2.2
ALA354B	−1.3 ± 0.4	−0.1 ± 0.1	−1.4 ± 0.4	−1.3
ASN258D	−4.9 ± 0.8	1.1 ± 0.6	−3.8 ± 1.0	−4.4
ASN349B	−0.8 ± 0.5	−0.4 ± 0.7	−1.2 ± 0.9	−0.4
ASN350B	−0.7 ± 0.2	−0.3 ± 0.4	−1.0 ± 0.5	−1.5
ASP251D	−0.3 ± 0.2	−0.4 ± 0.3	−0.7 ± 0.5	−0.2
CYS241B	−1.3 ± 0.7	−1.1 ± 1.1	−2.4 ± 1.6	−1.3
GLN247B	−0.4 ± 0.5	−0.3 ± 1.1	−0.7 ± 1.4	−8.0
ILE378B	−0.8 ± 0.3	0.0 ± 0.0	−0.8 ± 0.3	−0.8
LEU242D	−0.5 ± 0.4	0.1 ± 0.1	−0.4 ± 0.3	−0.1
LEU248D	−5.1 ± 0.9	−0.3 ± 0.3	−5.4 ± 1.0	−7.0
LEU255B	−5.6 ± 0.8	−0.1 ± 0.2	−5.8 ± 0.8	−5.1
LYS254B	−2.9 ± 0.6	1.2 ± 0.6	−1.8 ± 0.7	−2.1
LYS352D	−7.4 ± 1.1	−3.2 ± 1.8	−10.6 ± 2.1	−9.4
MET259B	−1.7 ± 0.5	−0.4 ± 0.3	−2.1 ± 0.6	−3.2
MET325B	−0.3 ± 0.3	−0.0 ± 0.1	−0.3 ± 0.3	−0.0
SER178C	−0.5 ± 0.3	0.1 ± 0.8	−0.4 ± 0.9	−1.2
THR179B	−1.0 ± 0.3	0.2 ± 0.4	−0.8 ± 0.5	−0.7
THR314B	−1.0 ± 0.3	0.5 ± 0.3	−0.5 ± 0.5	0.4
THR353B	−1.3 ± 0.4	0.5 ± 0.3	−0.7 ± 0.3	−0.5
VAL181C	−1.9 ± 0.7	−2.2 ± 1.0	−4.1 ± 1.2	−2.7
VAL182C	−0.2 ± 0.1	−0.1 ± 0.1	−0.3 ± 0.1	−0.2
VAL238B	−0.8 ± 0.6	−0.1 ± 0.3	−1.0 ± 0.7	−2.1
VAL315B	−0.9 ± 0.3	−0.5 ± 0.4	−1.4 ± 0.5	−1.0
VAL318B	−1.3 ± 0.4	−0.0 ± 0.1	−1.3 ± 0.4	−2.0
VAL351B	−1.0 ± 0.3	0.1 ± 0.2	−0.9 ± 0.4	−0.5
Total	−49.6 ± 13.5	−8.2 ± 12.5	−57.8 ± 19.9	−63.7

**Table 7:** Interaction energies (kcal/mol) between Tubulin and I5. Values are averages over the production trajectory (mean ± standard deviation); the last column reports the value in the final frame of the trajectory. The Total row is the sum over all the entries of the table.

Residue	Lennard-Jones (kcal/mol)	Coulomb (kcal/mol)	Total (kcal/mol)	Last Step (kcal/mol)
ALA180B	−0.8 ± 0.4	−0.1 ± 0.3	−0.8 ± 0.5	−0.3
ALA250B	−2.2 ± 0.7	−0.1 ± 0.3	−2.3 ± 0.8	−2.1
ALA316B	−1.2 ± 0.4	0.3 ± 0.3	−0.9 ± 0.5	−1.0
ALA317B	−0.2 ± 1.1	−6.1 ± 1.8	−6.3 ± 1.3	−8.0
ALA354B	−2.0 ± 0.4	0.3 ± 0.3	−1.6 ± 0.5	−2.5
ASN249D	−0.5 ± 0.4	−0.2 ± 0.5	−0.7 ± 0.7	−0.1
ASN258D	−1.1 ± 0.5	0.1 ± 0.5	−1.1 ± 0.7	−2.4
ASP251D	−2.0 ± 0.7	−0.8 ± 0.5	−2.8 ± 1.1	−2.3
CYS241B	−1.8 ± 0.6	−0.2 ± 0.2	−2.0 ± 0.6	−2.2
ILE378B	−0.9 ± 0.5	0.0 ± 0.0	−0.9 ± 0.5	−0.4
LEU248D	−1.2 ± 1.0	−0.3 ± 0.7	−1.5 ± 1.3	−0.0
LEU252D	−1.2 ± 0.5	−0.1 ± 0.1	−1.3 ± 0.6	−0.5
LEU255B	−4.3 ± 0.7	−0.0 ± 0.2	−4.3 ± 0.7	−4.3
LYS254B	−3.3 ± 1.0	−0.6 ± 1.1	−3.8 ± 1.5	−3.4
LYS352D	−3.6 ± 1.1	−0.8 ± 1.1	−4.4 ± 1.7	−3.7
SER178C	−0.6 ± 1.1	−3.7 ± 4.1	−4.3 ± 3.6	−1.2
THR179B	−0.7 ± 0.4	−0.3 ± 1.0	−1.0 ± 1.0	−1.5
THR353B	−1.6 ± 0.4	0.8 ± 0.6	−0.8 ± 0.7	−0.1
THR376B	−0.1 ± 0.1	−0.1 ± 0.1	−0.3 ± 0.1	−0.1
VAL238B	−0.5 ± 0.3	0.2 ± 0.2	−0.3 ± 0.2	−0.1
VAL318B	−1.6 ± 0.4	−0.6 ± 0.2	−2.1 ± 0.5	−1.7
Total	−31.5 ± 12.7	−12.1 ± 14.0	−43.6 ± 19.3	−37.9

**Table 8:** Interaction energies (kcal/mol) between etoposide and the Topoisomerase IIβ/DNA complex. Values are averages over the production trajectory (mean ± standard deviation); the last column reports the value in the final frame of the trajectory. The Total row is the sum over all the entries of the table.

**Residue**	**Lennard-Jones** **(kcal/mol)**	**Coulomb** **(kcal/mol)**	**Total** **(kcal/mol)**	**Last Step** **(kcal/mol)**
ARG503B	−1.5 ± 0.9	0.1 ± 0.7	−1.4 ± 1.5	−0.5
ARG820A	−0.2 ± 0.3	−0.1 ± 0.4	−0.3 ± 0.6	−1.4
ARG820B	−2.6 ± 1.1	−0.1 ± 1.7	−2.7 ± 1.9	−2.7
GLN778B	−2.8 ± 0.9	−0.4 ± 0.7	−3.2 ± 1.0	−3.2
GLY504B	−0.9 ± 1.2	−0.0 ± 0.5	−0.9 ± 1.4	−0.2
ILE784B	−0.6 ± 0.6	−0.0 ± 0.1	−0.6 ± 0.6	−0.4
ILE822B	−0.8 ± 0.6	0.0 ± 0.0	−0.7 ± 0.6	−1.4
LYS505B	−1.1 ± 1.5	0.2 ± 0.4	−1.0 ± 1.3	−0.0
MET781B	−4.0 ± 1.3	−0.8 ± 1.1	−4.8 ± 1.7	−5.5
MET782B	−5.6 ± 1.1	−0.4 ± 0.7	−6.0 ± 1.2	−4.7
PRO819B	−0.2 ± 0.3	−0.1 ± 0.2	−0.3 ± 0.4	−0.1
TYR821B	−0.8 ± 0.5	−0.4 ± 0.4	−1.2 ± 0.7	−1.8
VAL785B	−0.8 ± 0.5	−0.0 ± 0.1	−0.8 ± 0.6	−1.5
Total	−22.0 ± 10.8	−2.1 ± 7.0	−24.0 ± 13.5	−23.4
**Nucleotide**	**Lennard-Jones** **(kcal/mol)**	**Coulomb** **(kcal/mol)**	**Total** **(kcal/mol)**	**Last step** **(kcal/mol)**
DC8	−5.3 ± 2.2	−0.8 ± 0.9	−6.1 ± 2.7	−6.5
DG10	−2.5 ± 0.8	−0.2 ± 0.7	−2.7 ± 1.0	−2.7
Total	−7.9 ± 3.0	−1.0 ± 1.6	−8.8 ± 3.7	−9.2

**Table 9:** Interaction energies (kcal/mol) between I5 and the Topoisomerase IIβ/DNA complex. Values are averages over the production trajectory (mean ± standard deviation); the last column reports the value in the final frame of the trajectory. The Total row is the sum over all the entries of the table.

**Residue**	**Lennard-Jones** **(kcal/mol)**	**Coulomb** **(kcal/mol)**	**Total** **(kcal/mol)**	**Last Step** **(kcal/mol)**
ALA779B	−0.3 ± 0.3	0.0 ± 0.0	−0.3 ± 0.3	−0.6
ARG503B	−2.8 ± 1.0	−0.8 ± 0.6	−3.6 ± 0.9	−1.5
GLN778B	−2.4 ± 1.8	−1.7 ± 3.1	−4.2 ± 2.4	−2.8
GLU477B	−0.3 ± 0.6	−0.3 ± 1.3	−0.6 ± 1.3	−0.0
GLY478B	−0.2 ± 0.4	−0.0 ± 0.3	−0.3 ± 0.6	−0.0
GLY504B	−0.5 ± 0.3	−0.0 ± 0.1	−0.5 ± 0.3	−0.9
Total	−6.6 ± 4.5	−2.8 ± 5.4	−9.4 ± 5.8	−5.8
**Nucleotide**	**Lennard-Jones** **(kcal/mol)**	**Coulomb** **(kcal/mol)**	**Total** **(kcal/mol)**	**Last Step** **(kcal/mol)**
DC8	−4.0 ± 2.6	−7.5 ± 7.2	−11.5 ± 8.1	−21.5
DG10	−0.8 ± 0.8	−0.2 ± 1.2	−1.0 ± 1.5	−0.4
DG7	−0.7 ± 0.5	−0.5 ± 0.5	−1.2 ± 1.0	−1.8
Total	−5.5 ± 3.8	−8.2 ± 9.0	−13.7 ± 10.6	−23.7

## Data Availability

The original contributions presented in this study are included in the article/Supplementary Material. Further inquiries can be directed to the corresponding authors.

## Supplementary Materials

The following supporting information can be downloaded at https://www.mdpi.com/article/10.3390/ma19183980/s1, Figure S1: Conformational stability of the EGFR complexes over 100 ns of molecular dynamics: reference ligand 4-anilinoquinazoline versus I5; Figure S2: Conformational stability of the Tubulin complexes over 100 ns of molecular dynamics: reference ligand colchicine versus I5; Figure S3: Conformational stability of the Topoisomerase IIβ complexes over 100 ns of molecular dynamics: reference ligand etoposide versus I5; Table S1: Antioxidant activity of compounds I1–I6 and A1–A6; Table S2: Summary of cytotoxicity profiles and selectivity indices for all tested compounds; Table S3–S14: Cartesian coordinates and energies of compounds A1–A6 and I1–I6. ^1^H and ^13^C NMR spectra for the synthesized compounds and concentration-response curves for the DPPH, ABTS, FRAP, phenanthroline, and CUPRAC assays. Molecular dynamics inputs, trajectories and raw analysis data are available at https://doi.org/10.5281/zenodo.22671331.
