# Supplementary material for: Integrated Experimental and Computational Investigation of Salicylaldehyde-Derived Imine/Amine Derivatives as Antioxidant and Cytotoxic Agents
Source: Materials (Basel). 2026 Sep 19;19(18):3980. doi: 10.3390/ma19183980 (PMC13608854; doi:10.3390/ma19183980)
Supplement: Supplementary file 1 [file materials-19-03980-s001.zip › materials-4533518-supplementary.pdf]

Supplementary Material

Integrated Experimental and Computational Investigation of Salicylaldehyde-Derived Imine/Amine Derivatives as Antioxidant and Cytotoxic Agents

Rania Boukerzaza 1 , Chamseddine Derabli 1, Imene Amine Khodja 2, Abdellatif Ben Kaida 1 , Jose F. Costa-Rubio 3 , Chawki Benssouici 4, Stephanie Hesse 5 , Horacio Pérez-Sánchez 3,* and Houssem Boulebd 2,*

[1] Biotechnologies Laboratory, Higher National School of Biotechnology Taoufik Khaznadar, Nouveau Pôle Universitaire Ali Mendjeli, Constantine 25100, Algeria; raniaboukerzaza@gmail.com (R.B.); cderabli@yahoo.fr (C.D.); abdellatifbenkaida@gmail.com (A.B.K.)
[2] Laboratory of Synthesis of Molecules with Biological Interest, Frères Mentouri Constantine 1 University, Constantine 25017, Algeria; aminekhodja.imene@yahoo.fr
[3] Structural Bioinformatics and High Performance Computing Research Group (BIO-HPC), Universidad Católica de Murcia (UCAM), 30107 Murcia, Spain; jfcosta8@ucam.edu
[4] Biotechnology Research Center, Constantine 25000, Algeria; chawkiislam@yahoo.fr
[5] LCP-A2MC, Université de Lorraine, 5700 Metz, France; stephanie.hesse@univ-lorraine.fr

* Correspondence: hperez@ucam.edu (H.P.-S.); boulebd.houssem@umc.edu.dz (H.B.)

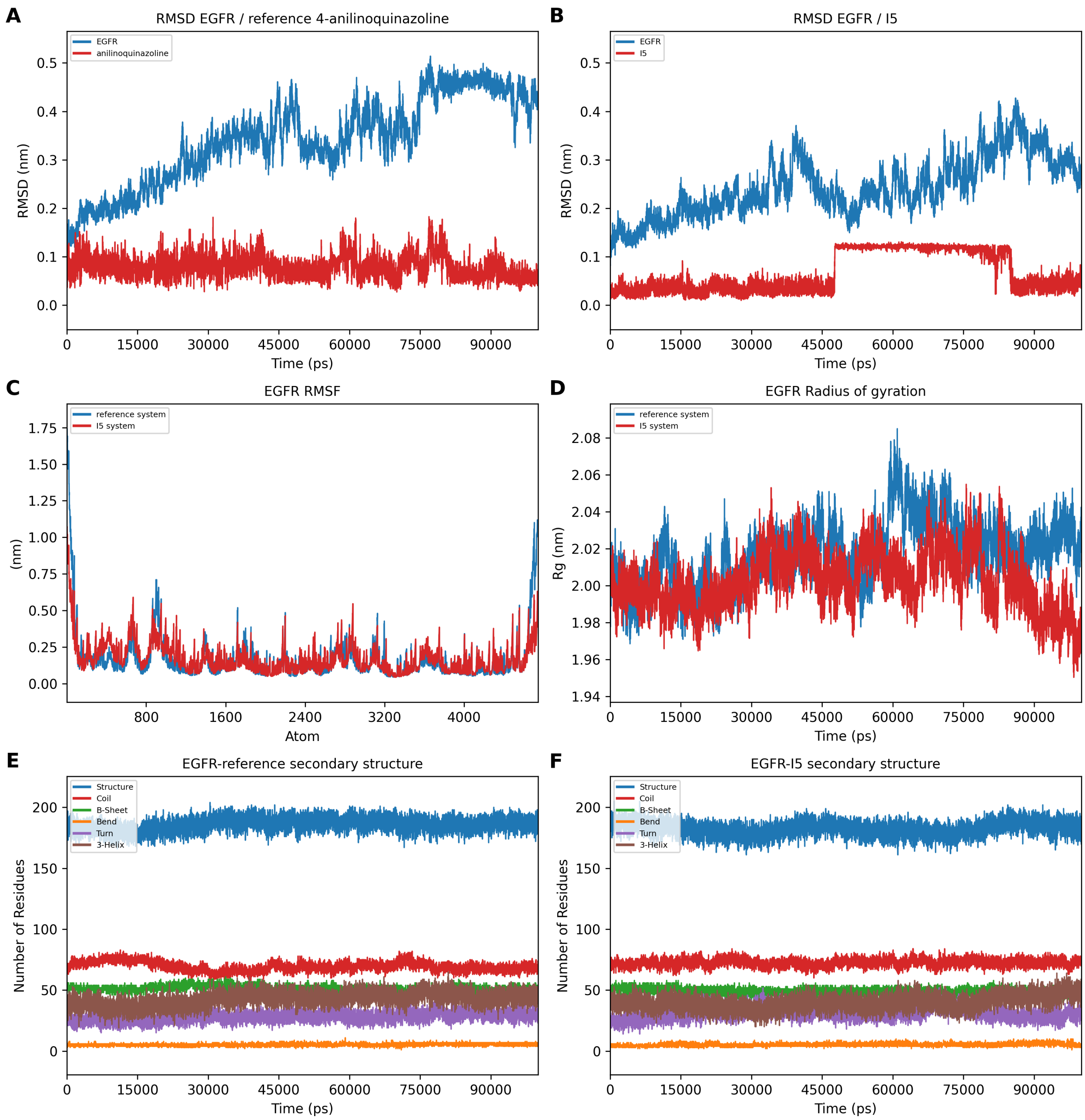

Figure S1. Conformational stability of the EGFR complexes over 100 ns of molecular dynamics: reference ligand 4-anilinoquinazoline versus I5. (A, B) RMSD of the EGFR backbone (blue) and of the bound ligand (red), for the reference and the I5 complex, respectively. Protein and ligand values were computed after least-squares fitting of the backbone and ligand onto themselves. (C) Per-atom root-mean-square fluctuation of EGFR for the reference (blue) and the I5 (red) system. (D) Total radius of gyration of EGFR for the reference (blue) and the I5 (red) system. (E, F) Number of residues assigned to each secondary-structure element by DSSP along the trajectory, for the reference and the I5 complex, respectively.

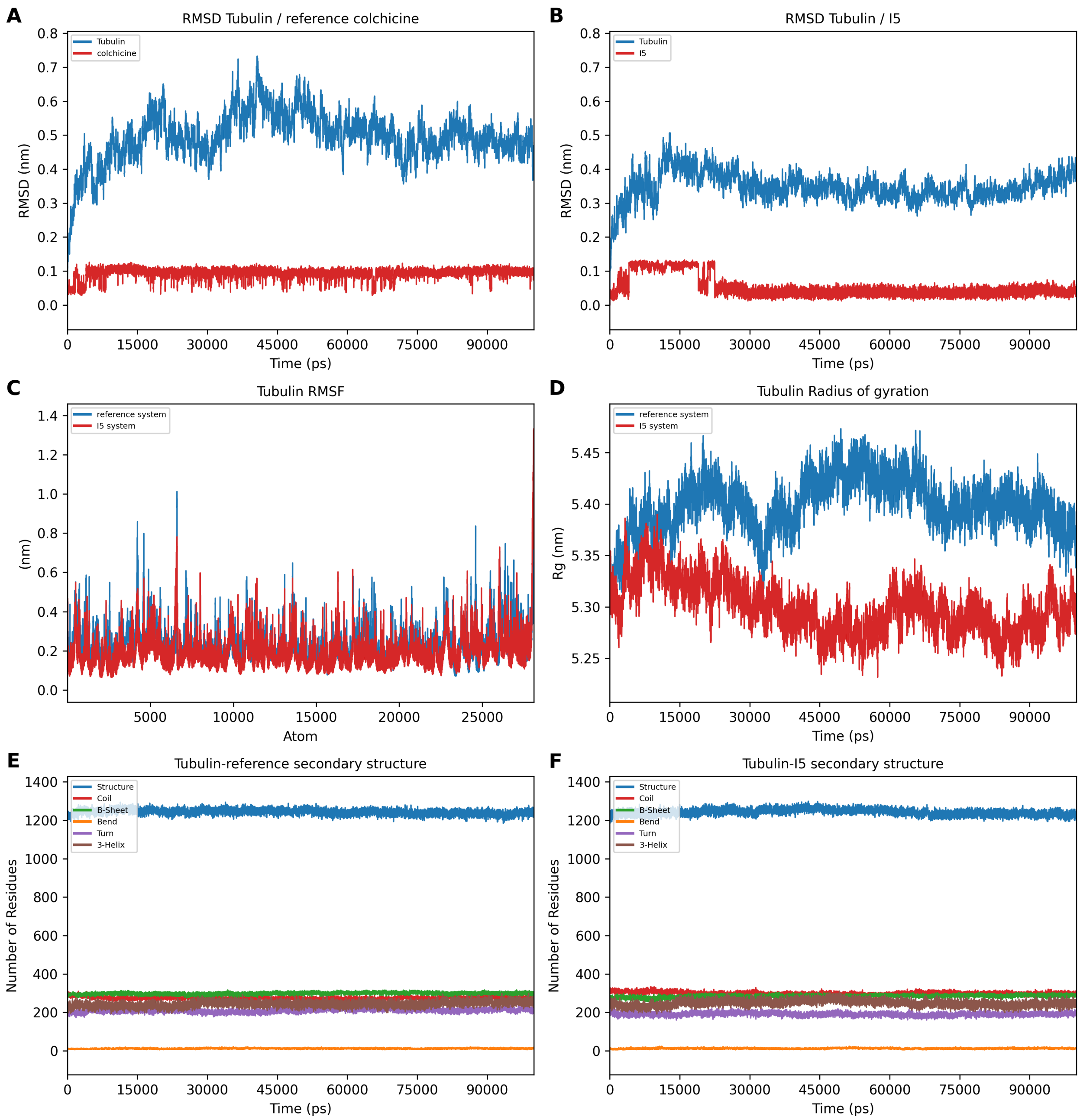

Figure S2. Conformational stability of the tubulin complexes over 100 ns of molecular dynamics: reference ligand colchicine versus I5. (A, B) RMSD of the tubulin backbone (blue) and of the bound ligand (red), for the reference and the I5 complex, respectively. Protein and ligand values were computed after least-squares fitting of the backbone and ligand onto themselves. (C) Per-atom root-mean-square fluctuation of tubulin for the reference (blue) and the I5 (red) system. (D) Total radius of gyration of tubulin for the reference (blue) and the I5 (red) system. (E, F) Number of residues assigned to each secondary-structure element by DSSP along the trajectory, for the reference and the I5 complex, respectively.

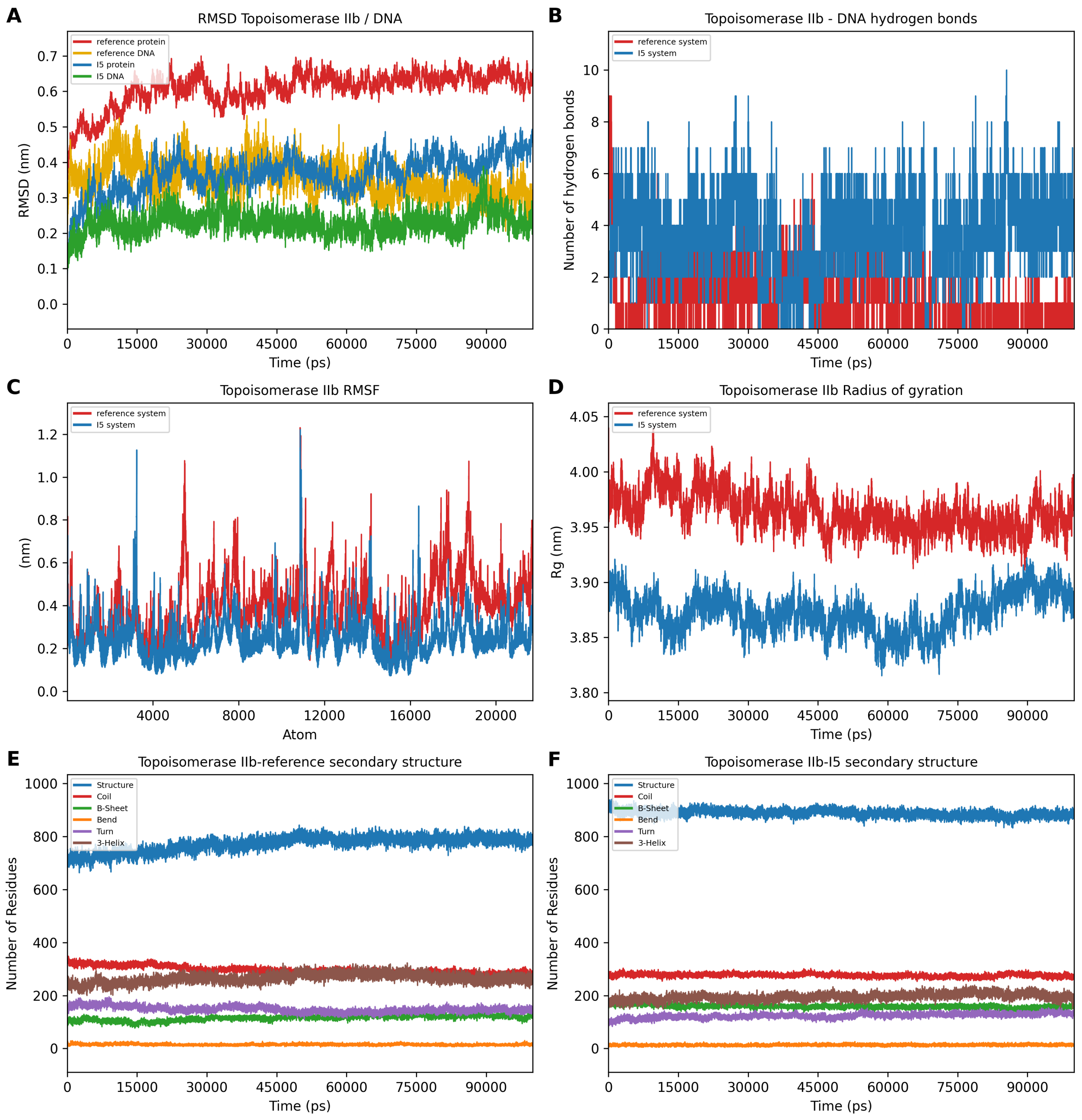

Figure S3. Conformational stability of the topoisomerase IIb complexes over 100 ns of molecular dynamics: reference ligand etoposide versus I5. (A) RMSD of the protein backbone and of the DNA for both systems: reference protein (red), reference DNA (yellow), I5 protein (blue) and I5 DNA (green); the ligand is not shown. Protein and DNA values were computed after least-squares fitting of the backbone and of the DNA onto themselves. (B) Number of hydrogen bonds between protein and DNA along the trajectory, for the reference (red) and the I5 (blue) system. (C) Per-atom root-mean-square fluctuation of topoisomerase IIb for the reference (red) and the I5 (blue) system. (D) Total radius of gyration of topoisomerase IIb for the reference (red) and the I5 (blue) system. (E, F) Number of residues assigned to each secondary-structure element by DSSP along the trajectory, for the reference and the I5 complex, respectively.

Table S1: Antioxidant activity (IC_{50} and $A_{0.50}$ in μM) of compounds I1-I6 and A1-A6.

Compounds	DPPH IC_{50}	ABTS IC_{50}	FRAP $A_{0.5}$	Phenanthroline $A_{0.5}$	CUPRAC $A_{0.5}$
I1	68.99 ± 3.19 ^{g}	16.88 ± 1.35 ^{efg}	23.48 ± 0.83 ^{d}	11.90 ± 0.36 ^{ab}	31.69 ± 0.63 ^{h}
I2	340.8 ± 0.9 ^{j}	10.18 ± 1.1 ^{b}	140.85 ± 2.27 ^{g}	6.38 ± 0.37 ^{ab}	28.06 ± 1.48 ^{fg}
I3	34.09 ± 2.58 ^{bc}	14.89 ± 0.22 ^{def}	18.08 ± 1.76 ^{b}	8.28 ± 0.45 ^{ab}	27.52 ± 0.68 ^{fg}
I4	322.95 ± 2.07 ^{i}	8.15 ± 0.62 ^{a}	> 200 ^{h}	5.89 ± 0.62 ^{ab}	35.94 ± 0.69 ^{i}
I5	45.69 ± 1.12 ^{e}	17.57 ± 0.41 ^{fg}	45.62 ± 1.94 ^{e}	11.54 ± 0.67 ^{ab}	29.59 ± 1.02 ^{gh}
I6	342.26 ± 4.24 ^{j}	8.23 ± 0.33 ^{a}	> 200 ^{h}	6.41 ± 0.38 ^{ab}	21.67 ± 1.51 ^{cd}
A1	35.45 ± 0.63 ^{bc}	31.1 ± 2.79 ^{i}	22.64 ± 0.47 ^{cd}	16.89 ± 2.4 ^{b}	21.26 ± 0.78 ^{cd}
A2	43.32 ± 0.36 ^{de}	30.05 ± 0.96 ^{i}	48.53 ± 4.6 ^{ef}	11.94 ± 1.37 ^{ab}	22.14 ± 1.03 ^{cd}
A3	38.81 ± 0.64 ^{cd}	29.44 ± 2.95 ^{i}	18.43 ± 2.83 ^{bc}	11.71 ± 0.86 ^{ab}	26.34 ± 1.89 ^{fg}
A4	29.95 ± 0.63 ^{ab}	13.59 ± 0.05 ^{cd}	22.16 ± 2.15 ^{bcd}	6.68 ± 0.95 ^{ab}	25.49 ± 0.5 ^{ef}
A5	34.42 ± 0.75 ^{bc}	19.15 ± 0.1 ^{gh}	19.53 ± 1.29 ^{bcd}	15.69 ± 1.17 ^{b}	23.05 ± 0.96 ^{de}
A6	26.7 ± 0.97 ^{a}	11.69 ± 0.43 ^{bc}	17.76 ± 0.81 ^{b}	5.25 ± 0.5 ^{a}	25.98 ± 0.35 ^{ef}
BHA	59.39 ± 1.9 ^{f}	14.31 ± 0.85 ^{de}	12.83 ± 0.86 ^{a}	14.09 ± 0.21 ^{ab}	19.37 ± 1.54 ^{bc}
BHT	85.85 ± 3.69 ^{h}	21.49 ± 1.13 ^{h}	12.80 ± 1.15 ^{a}	-	17.48 ± 0.97 ^{ab}
Acide Ascorbique	64.98 ± 1.84 ^{fg}	-	57.38 ± 5.75 ^{f}	-	15 ± 0.99 ^{a}

Means within the same column followed by same letter are not significantly different ($p < 0.05$)

Table S2: Summary of cytotoxicity profiles and selectivity indices for all tested compounds.

Compound	MDA-MB-231 IC_{50} (µM)	Vero IC_{50} (µM)	Selectivity Index
I1	ND	ND	ND
I2	54.9 ± 1.09	33.9 ± 1.29	0.62
I3	15.1 ± 1.99	ND	ND
I4	40.99 ± 2.94	14.97 ± 3.45	0.37
I5	122.6 ±2.23	>400	>3.26
I6	167.1 ± 7.58	>400	>2.39
A1	200 ± 1.09	>400	>2.00
A2	ND	80 ± 3.18	ND
A3	>400	50 ± 3.19	<0.13
A4	80 ± 8.16	70 ±3.37	0.88
A5	>400	>400	ND
A6	ND	70 ±2.21	ND

ND = Not determinable due to non-monotonic or biphasic dose-response curves.

Table S3: Cartesian coordinates and energies of "A1".

Energies (Hartree)	Cartesian Coordinates (Å)
Electronic Energy (EE) = -708.306996<br>Zero-point Energy Correction = 0.236918<br>Thermal Correction to Energy = 0.250771<br>Thermal Correction to Enthalpy = 0.251715<br>Thermal Correction to Free Energy = 0.195008<br>EE + Zero-point Energy = -708.070078<br>EE + Thermal Energy Correction = -708.056225<br>EE + Thermal Enthalpy Correction = -708.055281<br>EE + Thermal Free Energy Correction = -708.111988	C -3.525627 -0.954691 0.668960<br>C -4.298146 -0.013076 0.008762<br>C -3.674950 0.927296 -0.810101<br>C -2.301034 0.913258 -0.961390<br>C -1.502246 -0.030823 -0.297542<br>C -2.137542 -0.963708 0.522581<br>N -0.118070 -0.027852 -0.513345<br>C 0.729695 -0.757961 0.418928<br>C 2.185656 -0.479962 0.151914<br>C 3.043282 -1.447778 -0.359667<br>C 4.390924 -1.180142 -0.584469<br>C 4.893512 0.081857 -0.295930<br>C 4.054515 1.069404 0.209374<br>C 2.711260 0.786263 0.428375<br>O 1.837917 1.719563 0.914431<br>H -4.285249 1.660002 -1.323785<br>H -1.826156 1.640403 -1.611810<br>H -1.564119 -1.708190 1.059170<br>H 0.491489 -0.494673 1.461018<br>H 2.642065 -2.430616 -0.582991<br>H 5.040452 -1.950074 -0.981095<br>H 5.939989 0.305703 -0.464911<br>H 4.441384 2.058908 0.432877<br>H 2.306202 2.532803 1.121272<br>H 0.235322 0.904077 -0.686259<br>H 0.549517 -1.829301 0.296646<br>H -3.998271 -1.690052 1.313207<br>O -5.661979 0.049522 0.119127<br>H -5.969418 -0.656325 0.692838

Table S4: Cartesian coordinates and energies of "A2".

Energies (Hartree)	Cartesian Coordinates (Å)			
Electronic Energy (EE) = -708.310746<br>Zero-point Energy Correction = 0.236830<br>Thermal Correction to Energy = 0.250664<br>Thermal Correction to Enthalpy = 0.251609<br>Thermal Correction to Free Energy = 0.195126<br>EE + Zero-point Energy = -708.073916<br>EE + Thermal Energy Correction = -708.060082<br>EE + Thermal Enthalpy Correction = -708.059138<br>EE + Thermal Free Energy Correction = -708.115621	C	-3.707674	-0.660602	0.272779
	C	-4.394692	0.452785	-0.191634
	C	-3.660810	1.496384	-0.747068
	C	-2.281081	1.438495	-0.846845
	C	-1.589116	0.310413	-0.378271
	C	-2.317964	-0.740525	0.189554
	N	-0.208302	0.250073	-0.523775
	C	0.560346	-0.718954	0.243221
	C	2.038599	-0.499729	0.054877
	C	2.838520	-1.405820	-0.632642
	C	4.205779	-1.196506	-0.788805
	C	4.787613	-0.056147	-0.250269
	C	4.007706	0.868564	0.435654
	C	2.643613	0.645080	0.583585
	O	1.826003	1.524108	1.237972
	O	-4.445043	-1.669584	0.819796
	H	-5.472744	0.485474	-0.109231
	H	-4.181217	2.374380	-1.112266
	H	-1.725261	2.254323	-1.294727
	H	-1.815560	-1.621876	0.571527
	H	0.311395	-0.660344	1.313640
	H	2.376430	-2.292373	-1.053480
	H	4.808395	-1.916444	-1.327682
	H	5.850261	0.121830	-0.363391
	H	4.456139	1.763675	0.855502
	H	2.344608	2.244806	1.605423
	H	-3.862759	-2.378471	1.103989
	H	0.224859	1.162917	-0.546246
	H	0.310852	-1.725231	-0.103595

Table S5: Cartesian coordinates and energies of "A3".

Energies (Hartree)	Cartesian Coordinates (Å)			
Electronic Energy (EE) = -3281.878346<br>Zero-point Energy Correction = 0.226927<br>Thermal Correction to Energy = 0.242194<br>Thermal Correction to Enthalpy = 0.243138<br>Thermal Correction to Free Energy = 0.182411<br>EE + Zero-point Energy = -3281.651419<br>EE + Thermal Energy Correction = -3281.636152<br>EE + Thermal Enthalpy Correction = -3281.635208<br>EE + Thermal Free Energy Correction = -3281.695935	C	4.676426	-1.115552	-0.885847
	C	5.554304	-0.622984	0.068050
	C	5.062233	0.171780	1.100407
	C	3.707938	0.457282	1.177350
	C	2.809344	-0.031730	0.221907
	C	3.318748	-0.819159	-0.815122
	N	1.441864	0.246013	0.366767
	C	0.562176	0.021968	-0.770814
	C	-0.838349	0.478634	-0.454146
	C	-1.879105	-0.426688	-0.285364
	C	-3.163936	0.020071	-0.000197
	C	-3.434354	1.373248	0.126504
	C	-2.398534	2.287253	-0.032559
	C	-1.113317	1.843194	-0.318372
	O	-0.059632	2.698959	-0.472095
	O	6.880175	-0.941958	-0.055514
	Br	-4.562237	-1.244377	0.224494
	H	5.067114	-1.729520	-1.688044
	H	5.739134	0.566220	1.852391
	H	3.333599	1.062796	1.995936
	H	2.662311	-1.211107	-1.581141
	H	0.923496	0.543915	-1.670199
	H	-1.682715	-1.488329	-0.376017
	H	-4.436658	1.714428	0.349677
	H	-2.596290	3.349608	0.068708
	H	-0.362432	3.609690	-0.421705
	H	7.369734	-0.572447	0.683050
	H	1.284002	1.153392	0.785560
	H	0.538176	-1.047448	-0.992790

Table S6: Cartesian coordinates and energies of "A4".

Energies (Hartree)	Cartesian Coordinates (Å)			
Electronic Energy (EE) = -3281.881966<br>Zero-point Energy Correction = 0.226683<br>Thermal Correction to Energy = 0.242063<br>Thermal Correction to Enthalpy = 0.243007<br>Thermal Correction to Free Energy = 0.181852<br>EE + Zero-point Energy = -3281.655283<br>EE + Thermal Energy Correction = -3281.639903<br>EE + Thermal Enthalpy Correction = -3281.638959<br>EE + Thermal Free Energy Correction = -3281.700114	C	4.796011	-1.125998	-0.347538
	C	5.669081	-0.391031	0.443024
	C	5.149066	0.644040	1.213471
	C	3.796438	0.941432	1.208130
	C	2.916075	0.196170	0.408917
	C	3.431517	-0.839792	-0.377585
	N	1.554051	0.482079	0.449276
	C	0.687477	-0.005056	-0.611977
	C	-0.731077	0.465132	-0.417524
	C	-1.759944	-0.431559	-0.154885
	C	-3.067754	0.015867	-0.011043
	C	-3.372885	1.363413	-0.117298
	C	-2.349700	2.270450	-0.370314
	C	-1.042429	1.824879	-0.520879
	O	-0.001831	2.674083	-0.769311
	Br	-4.452578	-1.239689	0.318558
	O	5.326805	-2.133462	-1.097794
	H	6.722947	-0.635010	0.440388
	H	5.816545	1.230154	1.834751
	H	3.405052	1.741683	1.825573
	H	2.784479	-1.429145	-1.016981
	H	1.047750	0.317681	-1.600984
	H	-1.538266	-1.488404	-0.066553
	H	-4.392944	1.706535	-0.005085
	H	-2.575043	3.328856	-0.453056
	H	-0.324475	3.571954	-0.885439
	H	4.630084	-2.569957	-1.594453
	H	0.689806	-1.097168	-0.596233
	H	1.364900	1.444729	0.693480

Table S7: Cartesian coordinates and energies of "A5".

Energies (Hartree)	Cartesian Coordinates (Å)			
Electronic Energy (EE) = -1167.906483<br>Zero-point Energy Correction = 0.226807<br>Thermal Correction to Energy = 0.242092<br>Thermal Correction to Enthalpy = 0.243037<br>Thermal Correction to Free Energy = 0.182495<br>EE + Zero-point Energy = -1167.679676<br>EE + Thermal Energy Correction = -1167.664390<br>EE + Thermal Enthalpy Correction = -1167.663446<br>EE + Thermal Free Energy Correction = -1167.723988	C	4.112064	-0.986010	-0.869763
	C	4.961143	-0.387111	0.048238
	C	4.421102	0.399189	1.063281
	C	3.049049	0.572736	1.157429
	C	2.179001	-0.023573	0.237321
	C	2.735673	-0.801860	-0.782356
	N	0.795289	0.143784	0.398278
	C	-0.079014	-0.223358	-0.705469
	C	-1.508436	0.136926	-0.395177
	C	-2.471407	-0.836055	-0.157119
	C	-3.786565	-0.476266	0.110019
	C	-4.165006	0.855766	0.149990
	C	-3.207832	1.838121	-0.077545
	C	-1.892054	1.480972	-0.345011
	O	-0.911115	2.406780	-0.564228
	Cl	-4.974124	-1.719780	0.402175
	O	6.306971	-0.597167	-0.091306
	H	4.539776	-1.592741	-1.658497
	H	5.074787	0.874758	1.788372
	H	2.637927	1.171873	1.962941
	H	2.102708	-1.274726	-1.522014
	H	0.225218	0.268267	-1.642400
	H	-2.195152	-1.883678	-0.180884
	H	-5.192635	1.123554	0.359186
	H	-3.490602	2.885504	-0.044764
	H	-1.287111	3.290970	-0.576880
	H	6.776227	-0.162137	0.624658
	H	-0.016481	-1.303226	-0.860115
	H	0.565262	1.057588	0.766718

Table S8: Cartesian coordinates and energies of "A6".

Energies (Hartree)	Cartesian Coordinates (Å)			
Electronic Energy (EE) = -1167.910279<br>Zero-point Energy Correction = 0.227382<br>Thermal Correction to Energy = 0.242471<br>Thermal Correction to Enthalpy = 0.243415<br>Thermal Correction to Free Energy = 0.183310<br>EE + Zero-point Energy = -1167.682897<br>EE + Thermal Energy Correction = -1167.667809<br>EE + Thermal Enthalpy Correction = -1167.666864<br>EE + Thermal Free Energy Correction = -1167.726969	C	4.283284	-0.871393	-0.363007
	C	5.066946	-0.047614	0.434161
	C	4.431520	0.901425	1.228460
	C	3.052948	1.030523	1.238923
	C	2.263364	0.195242	0.433225
	C	2.893401	-0.755870	-0.376148
	N	0.877824	0.310950	0.489712
	C	0.060612	-0.277727	-0.559762
	C	-1.388092	0.092978	-0.376982
	C	-2.342216	-0.849006	-0.012738
	C	-3.673635	-0.481904	0.137133
	C	-4.076555	0.827892	-0.066278
	C	-3.129094	1.780145	-0.423197
	C	-1.797139	1.415585	-0.576724
	O	-0.824858	2.312937	-0.915825
	Cl	-4.852171	-1.687450	0.588067
	O	4.926662	-1.790626	-1.137944
	H	6.142605	-0.160443	0.419204
	H	5.029387	1.553022	1.855470
	H	2.571980	1.765877	1.873611
	H	2.314524	-1.409530	-1.018937
	H	0.398477	0.046659	-1.555548
	H	-2.046571	-1.877786	0.155506
	H	-5.116413	1.102258	0.054420
	H	-3.431891	2.810150	-0.581477
	H	-1.214874	3.171239	-1.102349
	H	4.283843	-2.305624	-1.631912
	H	0.150511	-1.365939	-0.512448
	H	0.574054	1.239969	0.747109

Table S9: Cartesian coordinates and energies of "I1".

Energies (Hartree)	Cartesian Coordinates (Å)			
Electronic Energy (EE) = -707.108971<br>Zero-point Energy Correction = 0.213395<br>Thermal Correction to Energy = 0.226184<br>Thermal Correction to Enthalpy = 0.227128<br>Thermal Correction to Free Energy = 0.173257<br>EE + Zero-point Energy = -706.895576<br>EE + Thermal Energy Correction = -706.882787<br>EE + Thermal Enthalpy Correction = -706.881843<br>EE + Thermal Free Energy Correction = -706.935714	C	-3.461095	-1.153393	0.532456
	C	-4.272101	-0.165213	-0.019131
	C	-3.700484	1.000900	-0.523137
	C	-2.324252	1.172962	-0.479238
	C	-1.499742	0.175727	0.039700
	C	-2.085917	-0.981731	0.561891
	N	-0.107222	0.403104	0.052491
	C	0.703060	-0.565674	-0.154990
	C	2.148600	-0.401163	-0.106948
	C	2.975522	-1.504450	-0.361211
	C	4.352667	-1.393208	-0.327284
	C	4.923900	-0.151666	-0.032790
	C	4.132666	0.954954	0.221411
	C	2.739560	0.847134	0.187809
	O	2.011585	1.940650	0.437414
	H	-4.332073	1.777836	-0.942127
	H	-1.871121	2.079317	-0.862710
	H	-1.463876	-1.737247	1.027924
	H	0.335052	-1.570523	-0.394787
	H	2.510051	-2.458792	-0.587125
	H	4.978912	-2.253048	-0.526550
	H	6.002610	-0.049222	-0.003099
	H	4.563938	1.921837	0.449008
	H	1.060202	1.694908	0.369575
	O	-5.615425	-0.387230	-0.022975
	H	-6.071079	0.373439	-0.392599
	H	-3.925651	-2.038667	0.948314

Table S10: Cartesian coordinates and energies of "I2".

<table>
<tr><th>Energies (Hartree)</th><th colspan="4">Cartesian Coordinates (Å)</th></tr>
<tr><td rowspan="26">Electronic Energy (EE) = -707.109282
Zero-point Energy Correction = 0.213113
Thermal Correction to Energy = 0.226047
Thermal Correction to Enthalpy = 0.226991
Thermal Correction to Free Energy = 0.172754
EE + Zero-point Energy = -706.896169
EE + Thermal Energy Correction = -706.883235
EE + Thermal Enthalpy Correction = -706.882291
EE + Thermal Free Energy Correction = -706.936528</td><td>C</td><td>-3.643556</td><td>0.788462</td><td>-0.257498</td></tr>
<tr><td>C</td><td>-4.375880</td><td>-0.280684</td><td>0.251185</td></tr>
<tr><td>C</td><td>-3.708918</td><td>-1.432654</td><td>0.645757</td></tr>
<tr><td>C</td><td>-2.326799</td><td>-1.532978</td><td>0.541269</td></tr>
<tr><td>C</td><td>-1.597182</td><td>-0.448376</td><td>0.057812</td></tr>
<tr><td>C</td><td>-2.257642</td><td>0.711781</td><td>-0.355816</td></tr>
<tr><td>N</td><td>-0.195193</td><td>-0.573002</td><td>-0.035327</td></tr>
<tr><td>C</td><td>0.555435</td><td>0.410113</td><td>0.292772</td></tr>
<tr><td>C</td><td>2.005055</td><td>0.361482</td><td>0.178336</td></tr>
<tr><td>C</td><td>2.763973</td><td>1.472748</td><td>0.574012</td></tr>
<tr><td>C</td><td>4.142274</td><td>1.471229</td><td>0.479636</td></tr>
<tr><td>C</td><td>4.784715</td><td>0.334336</td><td>-0.020846</td></tr>
<tr><td>C</td><td>4.062350</td><td>-0.777128</td><td>-0.416962</td></tr>
<tr><td>C</td><td>2.667451</td><td>-0.780762</td><td>-0.322774</td></tr>
<tr><td>O</td><td>2.009380</td><td>-1.876163</td><td>-0.714035</td></tr>
<tr><td>O</td><td>-4.340229</td><td>1.887420</td><td>-0.657625</td></tr>
<tr><td>H</td><td>-5.453341</td><td>-0.195527</td><td>0.311096</td></tr>
<tr><td>H</td><td>-4.277856</td><td>-2.269290</td><td>1.033384</td></tr>
<tr><td>H</td><td>-1.799735</td><td>-2.430403</td><td>0.838854</td></tr>
<tr><td>H</td><td>-1.694123</td><td>1.531199</td><td>-0.791406</td></tr>
<tr><td>H</td><td>0.127989</td><td>1.337250</td><td>0.693801</td></tr>
<tr><td>H</td><td>2.244264</td><td>2.343857</td><td>0.960674</td></tr>
<tr><td>H</td><td>4.716111</td><td>2.335761</td><td>0.787165</td></tr>
<tr><td>H</td><td>5.865580</td><td>0.319285</td><td>-0.100938</td></tr>
<tr><td>H</td><td>4.548654</td><td>-1.663256</td><td>-0.805232</td></tr>
<tr><td>H</td><td>1.047082</td><td>-1.722092</td><td>-0.577806</td></tr>
<tr><td></td><td>H</td><td>-3.739139</td><td>2.548754</td><td>-1.010322</td></tr>
</table>

Table S11: Cartesian coordinates and energies of "I3".

Energies (Hartree)	Cartesian Coordinates (Å)			
Electronic Energy (EE) = -3280.680274<br>Zero-point Energy Correction = 0.203316<br>Thermal Correction to Energy = 0.217616<br>Thermal Correction to Enthalpy = 0.218561<br>Thermal Correction to Free Energy = 0.160024<br>EE + Zero-point Energy = -3280.476958<br>EE + Thermal Energy Correction = -3280.462658<br>EE + Thermal Enthalpy Correction = -3280.461714<br>EE + Thermal Free Energy Correction = -3280.520251	C	-4.525052	-1.452375	0.661346
	C	-5.506779	-0.765748	-0.048759
	C	-5.190268	0.425510	-0.698052
	C	-3.895713	0.922156	-0.642306
	C	-2.897156	0.223343	0.034141
	C	-3.230567	-0.959592	0.701477
	N	-1.593115	0.764676	0.045068
	C	-0.580938	-0.014738	-0.011410
	C	0.788830	0.480686	0.043245
	C	1.844806	-0.435600	-0.051785
	C	3.152169	0.001625	-0.004947
	C	3.438030	1.359543	0.139535
	C	2.405723	2.275410	0.233904
	C	1.073169	1.855547	0.184637
	O	0.112787	2.779698	0.273115
	O	-6.758608	-1.298886	-0.058403
	Br	4.573045	-1.248838	-0.150820
	H	-4.798117	-2.358502	1.187449
	H	-5.957568	0.968096	-1.241139
	H	-3.640624	1.849959	-1.140079
	H	-2.479597	-1.478591	1.285993
	H	1.625015	-1.491271	-0.164977
	H	4.467385	1.693350	0.175278
	H	2.605813	3.333685	0.345131
	H	-0.757669	2.322832	0.215204
	H	-7.356515	-0.725220	-0.544679
	H	-0.703829	-1.098821	-0.118816

Table S12: Cartesian coordinates and energies of "I4".

Energies (Hartree)	Cartesian Coordinates (Å)			
Electronic Energy (EE) = -3280.680444<br>Zero-point Energy Correction = 0.203155<br>Thermal Correction to Energy = 0.217485<br>Thermal Correction to Enthalpy = 0.218429<br>Thermal Correction to Free Energy = 0.159789<br>EE + Zero-point Energy = -3280.477288<br>EE + Thermal Energy Correction = -3280.462958<br>EE + Thermal Enthalpy Correction = -3280.462014<br>EE + Thermal Free Energy Correction = -3280.520655	C	-4.678932	-1.280133	-0.343115
	C	-5.614415	-0.551879	0.387311
	C	-5.234591	0.648625	0.971551
	C	-3.938551	1.133251	0.838535
	C	-3.000417	0.384416	0.131077
	C	-3.372443	-0.819213	-0.473207
	N	-1.687185	0.889850	0.013951
	C	-0.691308	0.097860	0.138234
	C	0.685778	0.545834	-0.022631
	C	1.724078	-0.379539	0.151134
	C	3.037504	0.010778	-0.004909
	C	3.346725	1.330674	-0.336957
	C	2.332532	2.255052	-0.508978
	C	0.993760	1.882394	-0.354680
	O	0.053089	2.813444	-0.529921
	Br	4.433426	-1.253631	0.230665
	O	-5.101545	-2.437782	-0.919753
	H	-6.624339	-0.932520	0.469574
	H	-5.963994	1.219787	1.533245
	H	-3.636159	2.072099	1.284397
	H	-2.655409	-1.369222	-1.074750
	H	1.485061	-1.405451	0.408807
	H	4.380685	1.627820	-0.458369
	H	2.551757	3.284151	-0.764906
	H	-0.825653	2.396618	-0.379062
	H	-4.384967	-2.838783	-1.418514
	H	-0.837283	-0.960371	0.385870

Table S13: Cartesian coordinates and energies of "I5".

Energies (Hartree)	Cartesian Coordinates (Å)			
Electronic Energy (EE) = -1166.708367<br>Zero-point Energy Correction = 0.203486<br>Thermal Correction to Energy = 0.217665<br>Thermal Correction to Enthalpy = 0.218609<br>Thermal Correction to Free Energy = 0.160130<br>EE + Zero-point Energy = -1166.504880<br>EE + Thermal Energy Correction = -1166.490702<br>EE + Thermal Enthalpy Correction = -1166.489757<br>EE + Thermal Free Energy Correction = -1166.548237	C	4.001022	-1.354518	-0.575973
	C	4.923955	-0.516738	0.045464
	C	4.503081	0.687113	0.606102
	C	3.163990	1.045210	0.549159
	C	2.226212	0.198202	-0.039386
	C	2.662770	-0.997707	-0.618658
	N	0.878979	0.617074	-0.057270
	C	-0.066589	-0.237513	0.047841
	C	-1.470726	0.150393	-0.005828
	C	-2.455685	-0.838121	0.124434
	C	-3.791396	-0.499307	0.079850
	C	-4.177906	0.829751	-0.091111
	C	-3.217674	1.816456	-0.219142
	C	-1.857299	1.496318	-0.180456
	O	-0.969205	2.485925	-0.309828
	Cl	-5.009660	-1.738361	0.235071
	O	6.223555	-0.919056	0.057081
	H	4.353911	-2.270744	-1.032719
	H	5.222950	1.348446	1.077682
	H	2.825855	1.982853	0.974051
	H	1.957426	-1.636216	-1.137873
	H	-2.162484	-1.873167	0.260065
	H	-5.230887	1.080662	-0.122971
	H	-3.495924	2.854073	-0.353278
	H	-0.066989	2.092498	-0.258773
	H	6.769148	-0.254997	0.486530
	H	0.141029	-1.302988	0.200185

Table S14: Cartesian coordinates and energies of "I6".

Energies (Hartree)	Cartesian Coordinates (Å)			
Electronic Energy (EE) = -1166.708556<br>Zero-point Energy Correction = 0.203611<br>Thermal Correction to Energy = 0.217791<br>Thermal Correction to Enthalpy = 0.218735<br>Thermal Correction to Free Energy = 0.161042<br>EE + Zero-point Energy = -1166.504945<br>EE + Thermal Energy Correction = -1166.490765<br>EE + Thermal Enthalpy Correction = -1166.489821<br>EE + Thermal Free Energy Correction = -1166.547514	C	-4.174347	-1.072840	-0.323032
	C	-5.033381	-0.223060	0.368958
	C	-4.532531	0.950591	0.915542
	C	-3.191509	1.289559	0.781038
	C	-2.332274	0.420254	0.112300
	C	-2.824822	-0.758705	-0.453040
	N	-0.973511	0.783714	-0.006527
	C	-0.061424	-0.095637	0.162952
	C	1.354443	0.214380	0.011945
	C	2.299377	-0.794698	0.243516
	C	3.645355	-0.531801	0.104701
	C	4.081560	0.740007	-0.267790
	C	3.161659	1.745858	-0.497981
	C	1.791247	1.503534	-0.360738
	O	0.945026	2.510794	-0.590078
	Cl	4.813697	-1.793152	0.398420
	O	-4.713113	-2.199097	-0.863837
	H	-6.079039	-0.490289	0.451076
	H	-5.201390	1.616158	1.447928
	H	-2.794895	2.207308	1.195979
	H	-2.165375	-1.402730	-1.026783
	H	1.966910	-1.784797	0.534776
	H	5.142056	0.931990	-0.373616
	H	3.479652	2.739630	-0.786624
	H	0.028880	2.182961	-0.441028
	H	-4.038380	-2.695848	-1.334029
	H	-0.312119	-1.125033	0.445963

H NMR and ^{13}C NMR spectra of the synthesized compounds

4-((2-hydroxybenzylidene) amino) phenol (I1)

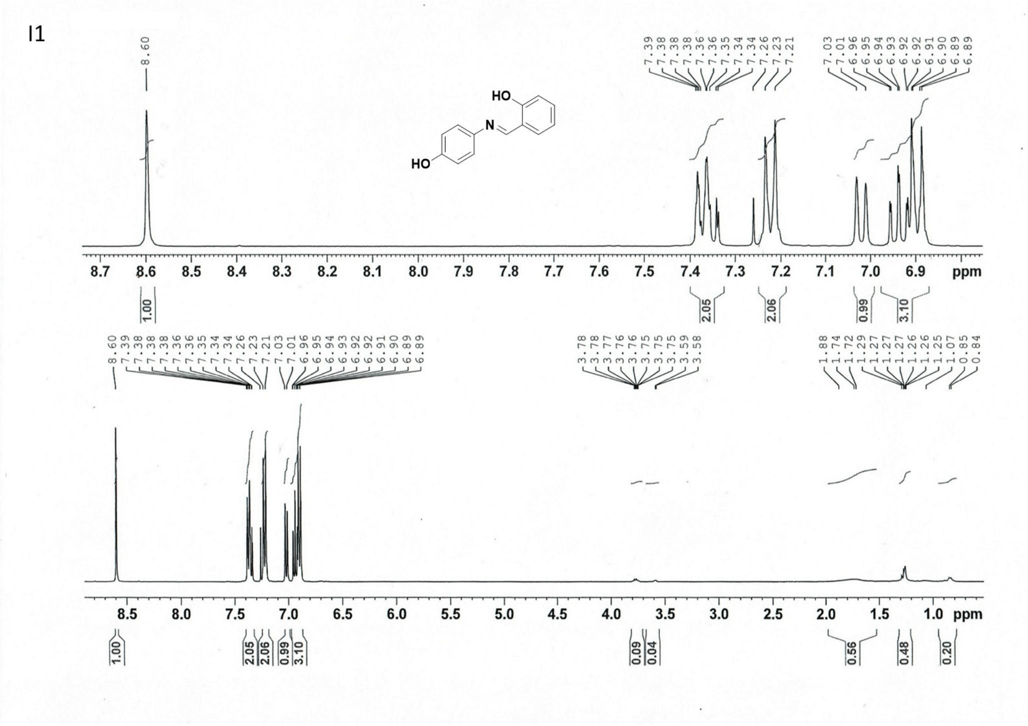

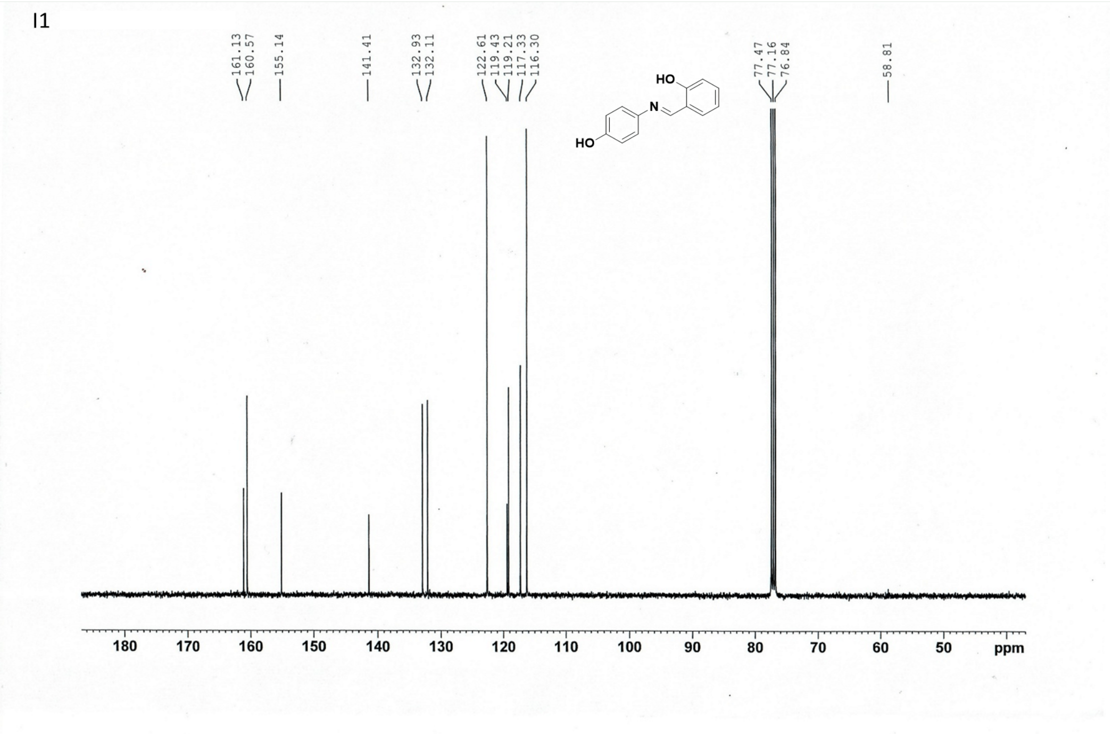

3-((2-hydroxybenzylidene) amino) phenol (I2)

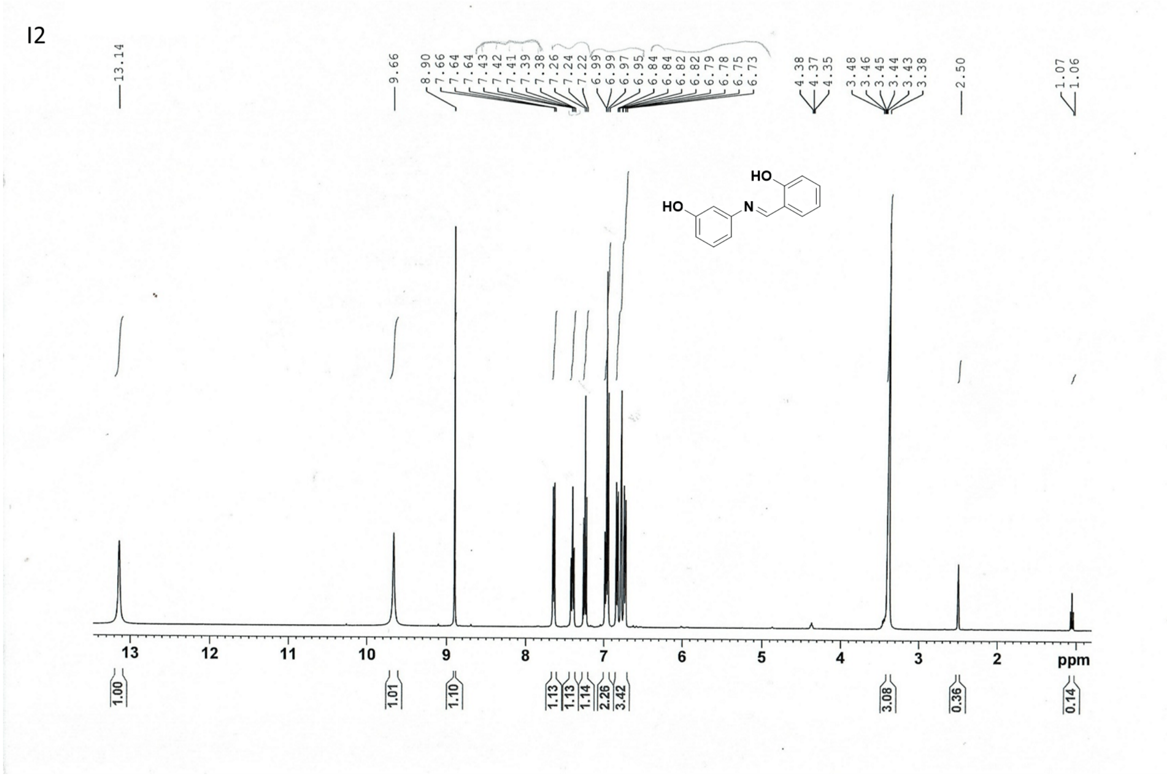

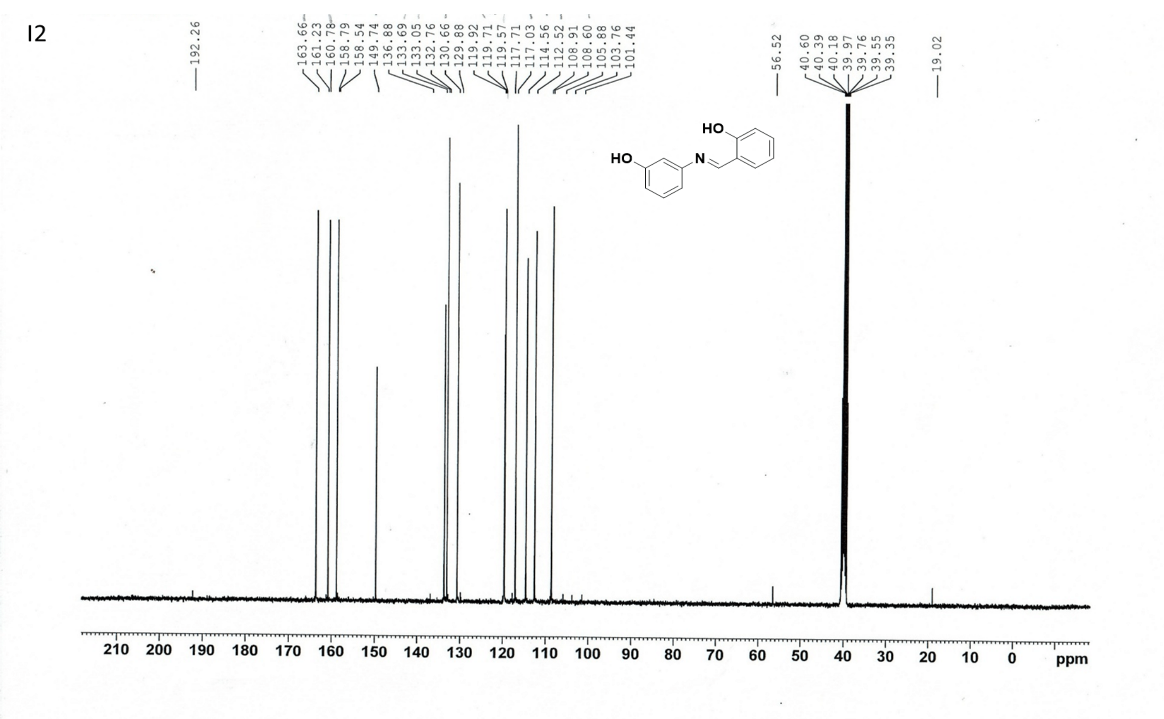

4-((5-Bromo-2-hydroxybenzylidene) amino) phenol (I3)

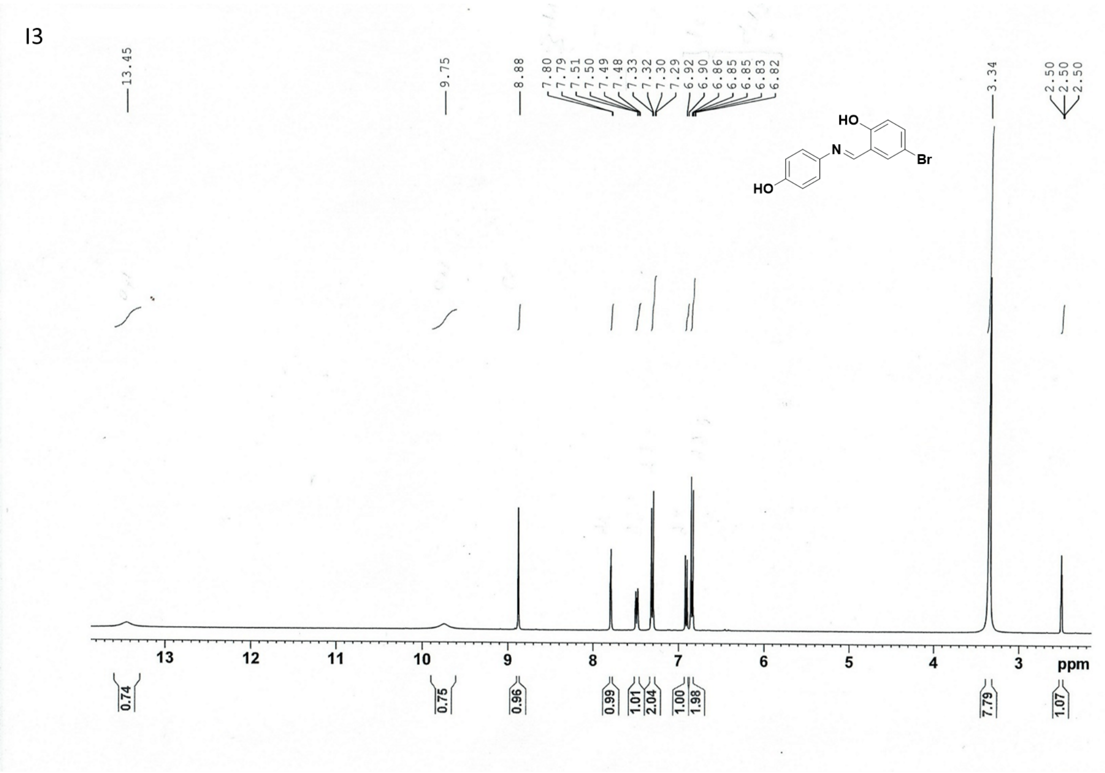

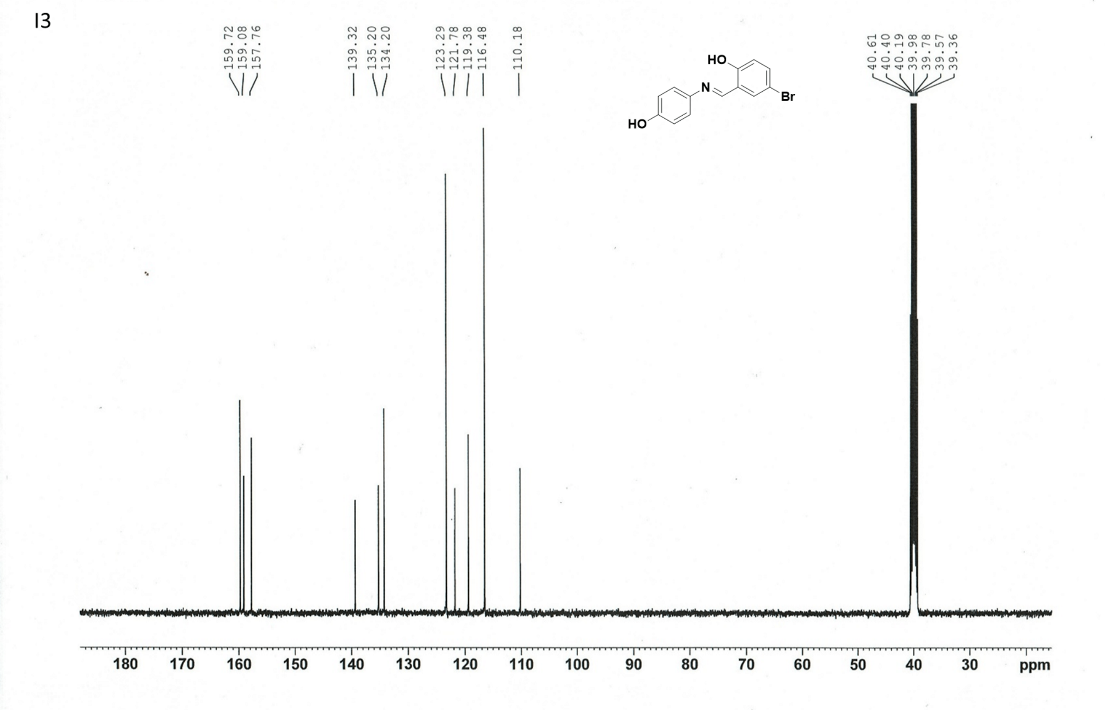

3-((5-Bromo-2-hydroxybenzylidene) amino) phenol (I4)

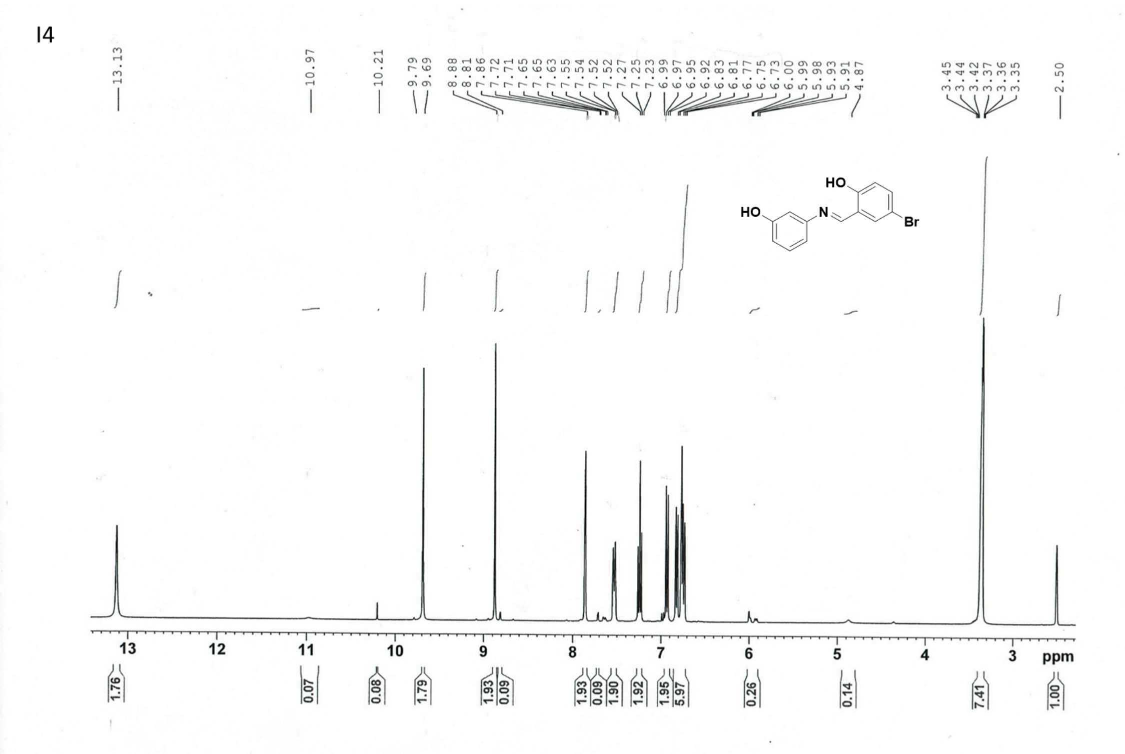

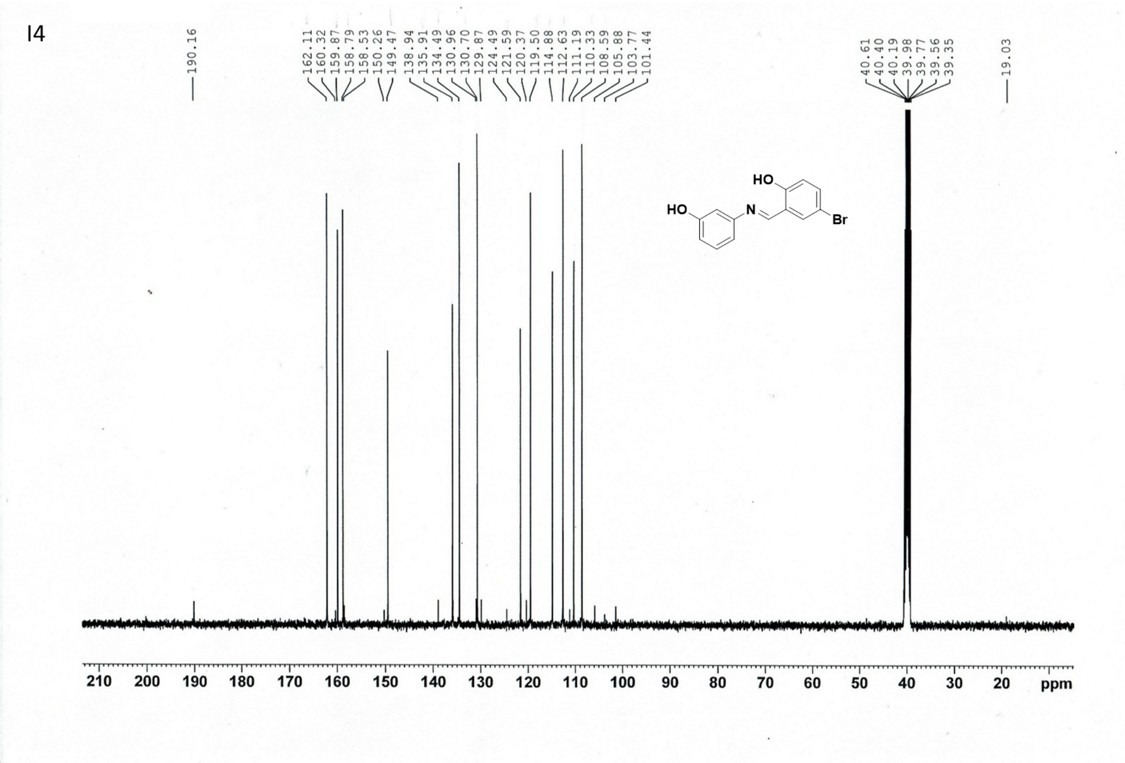

4-((5-Chloro-2-hydroxybenzylidene) amino) phenol (I5)

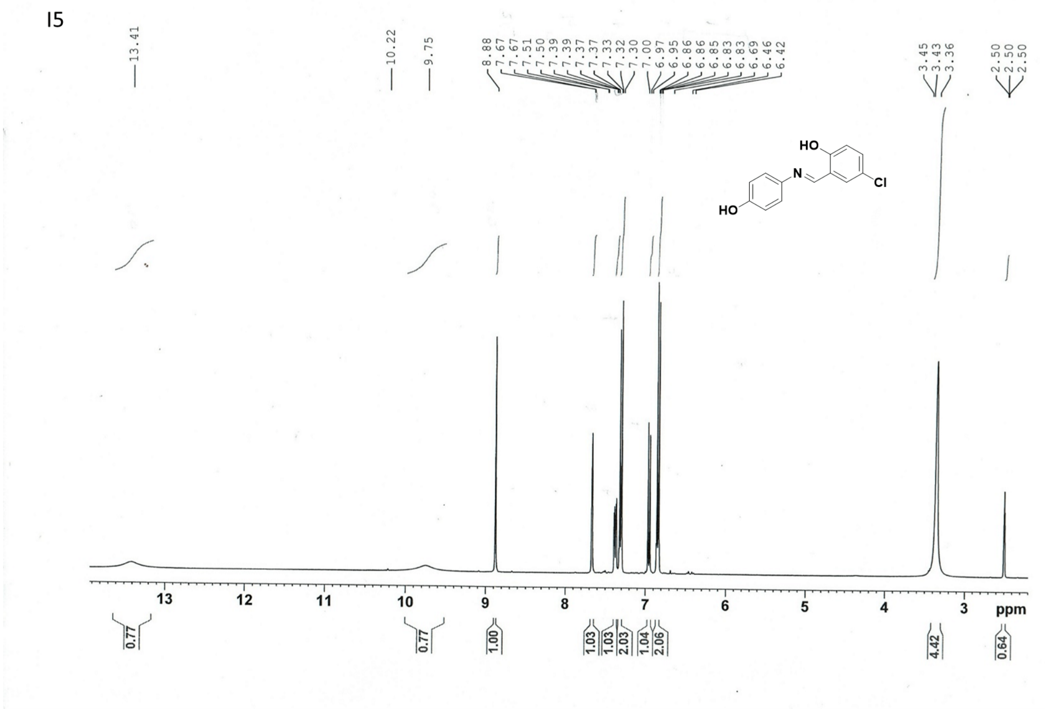

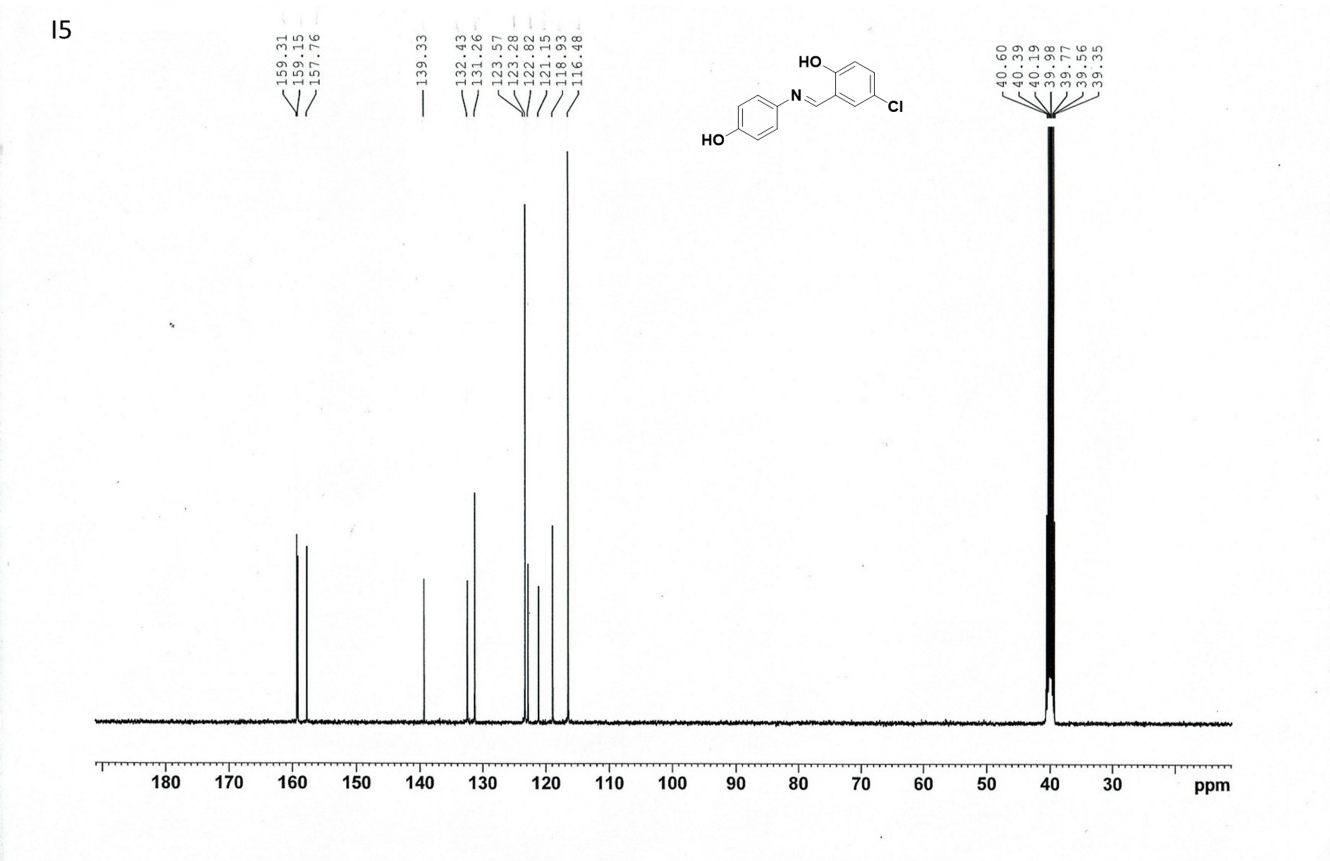

3-((5-Chloro-2-hydroxybenzylidene) amino) phenol (I6)

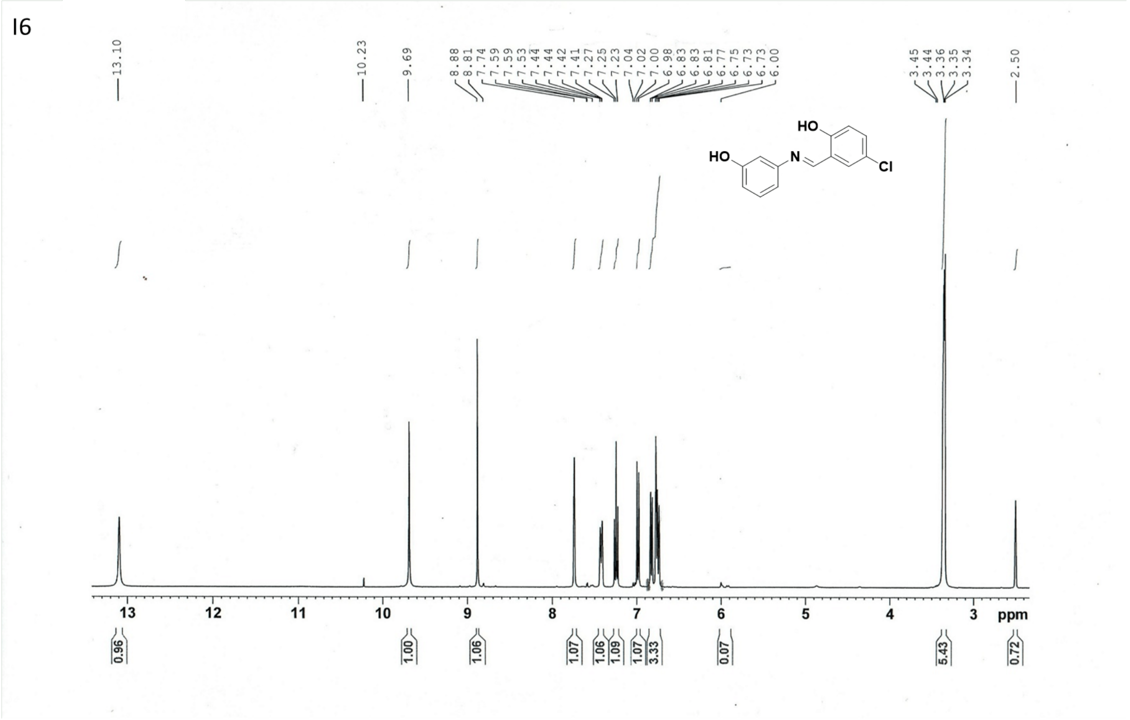

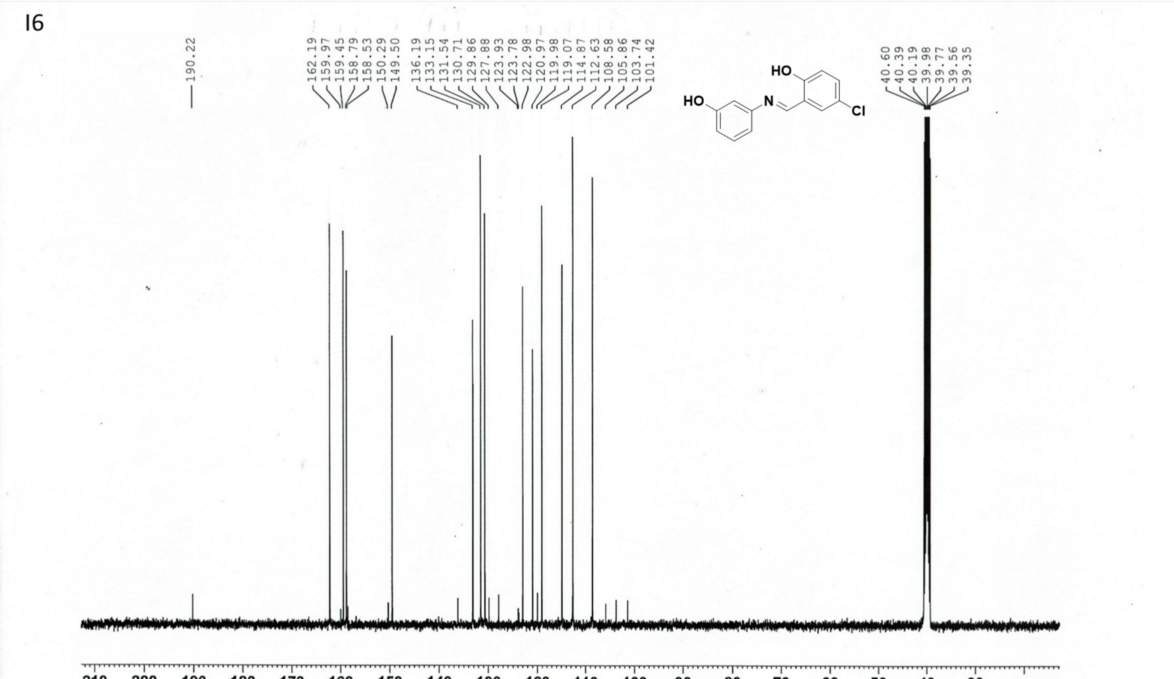

4-((2-hydroxybenzyl) amino) phenol (A1)

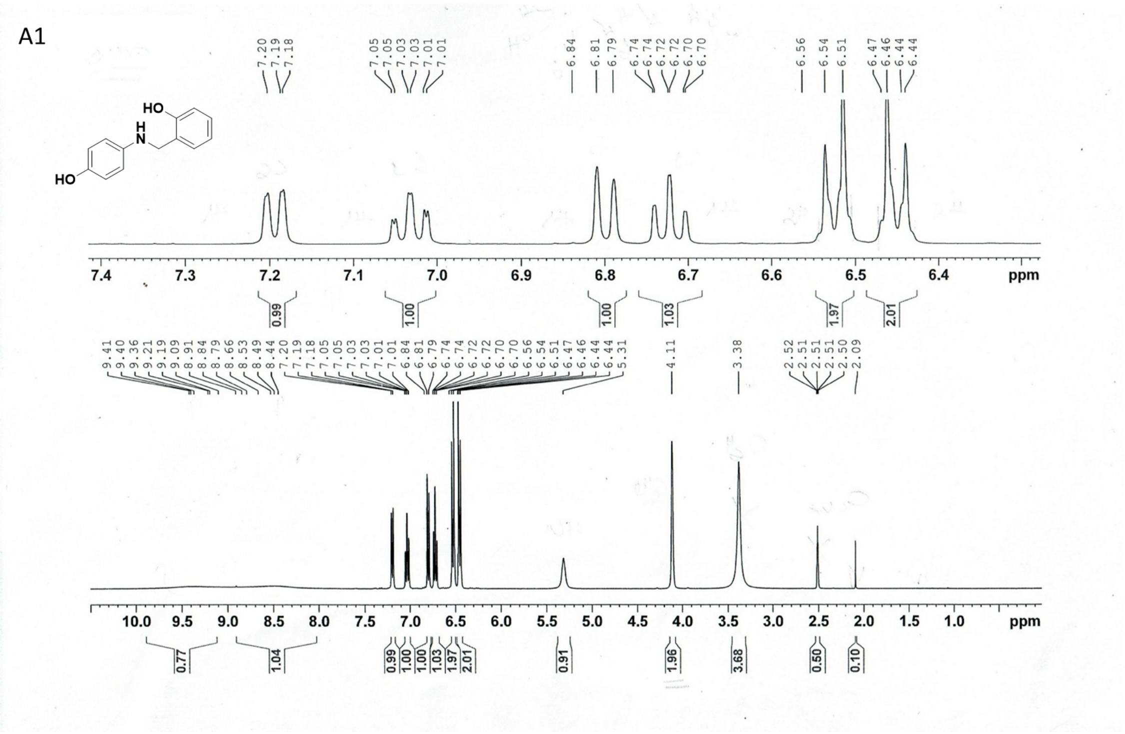

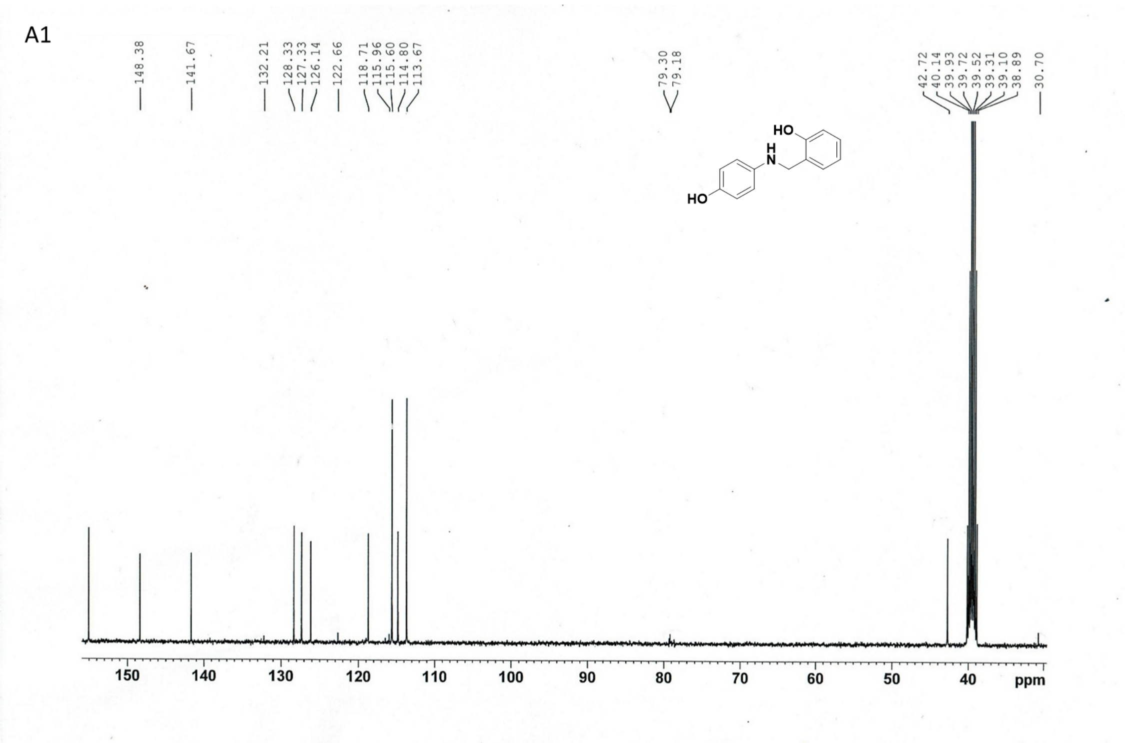

3-((2-hydroxybenzyl) amino) phenol (A2)

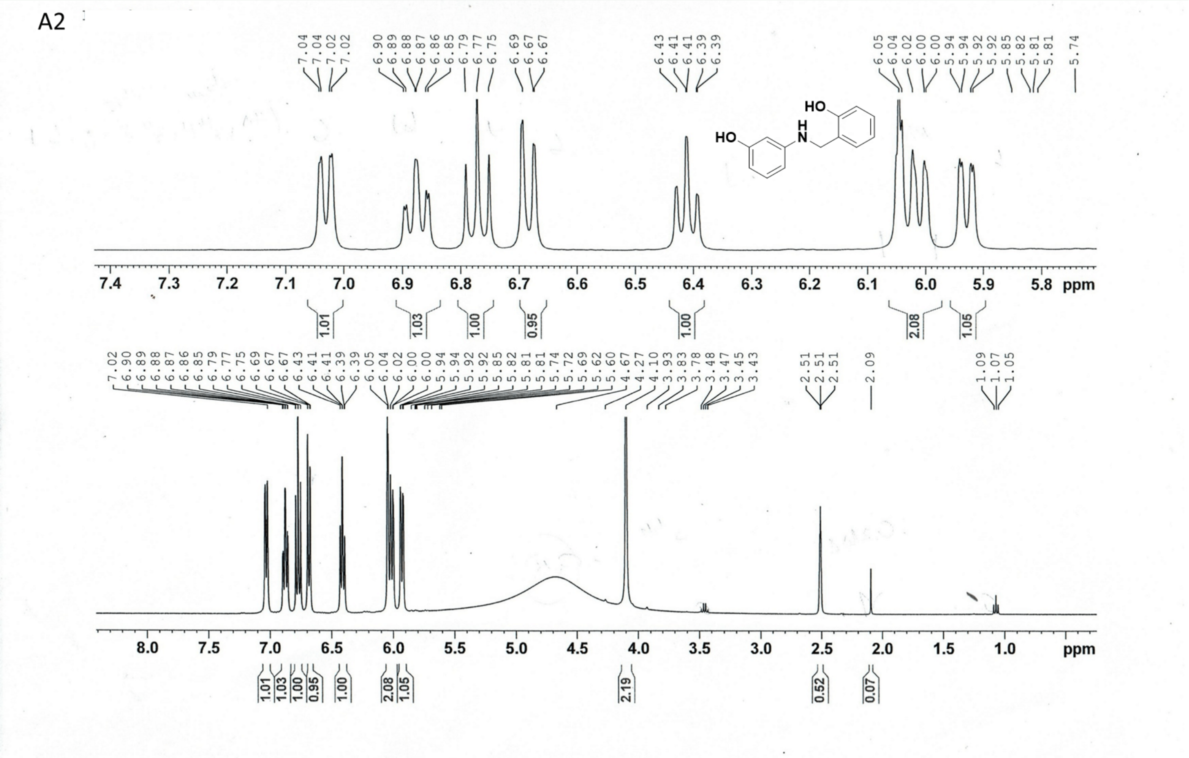

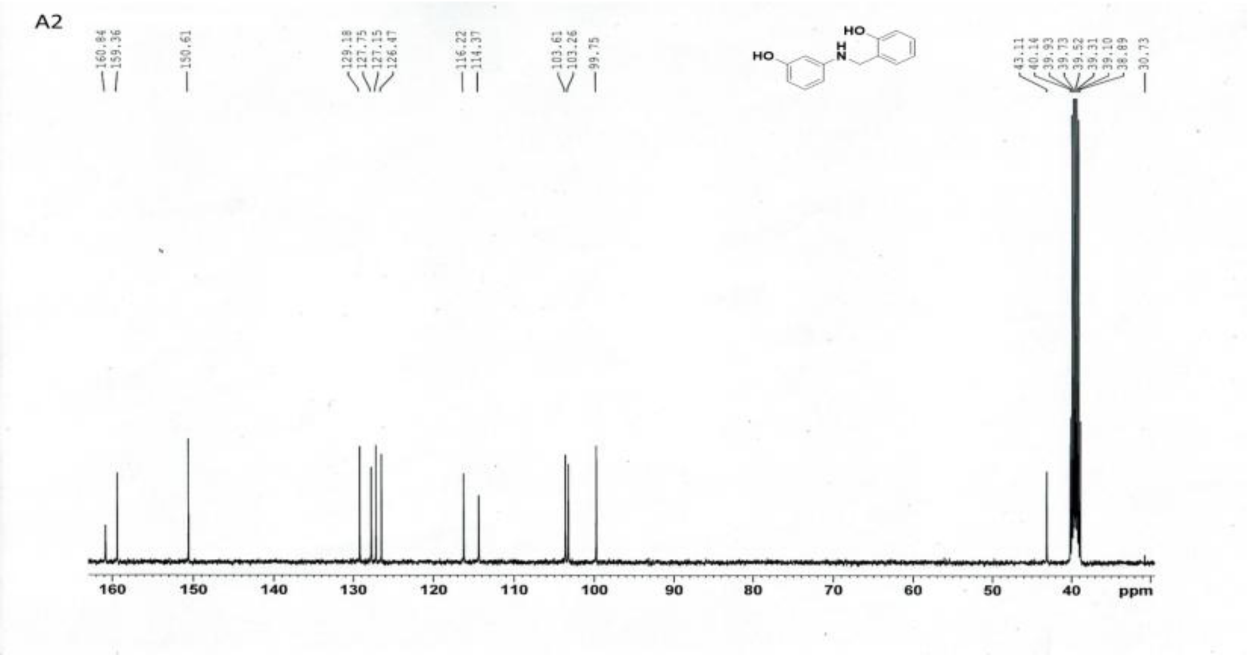

4-((5-Bromo-2-hydroxybenzyl) amino) phenol (A3)

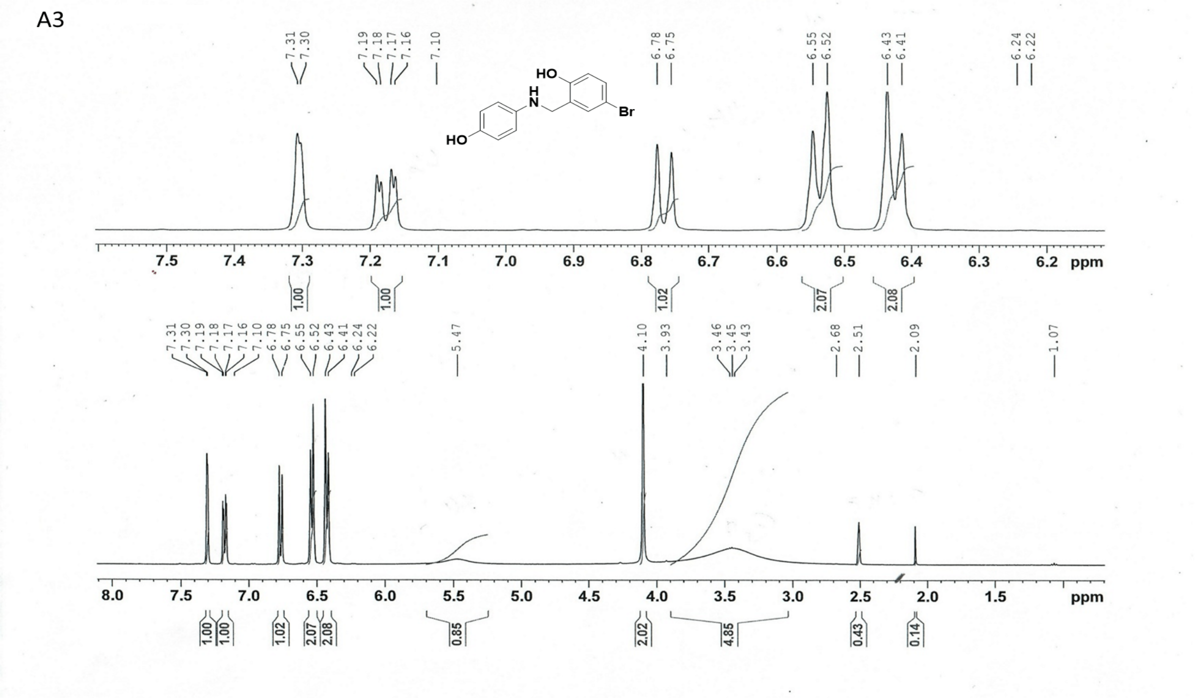

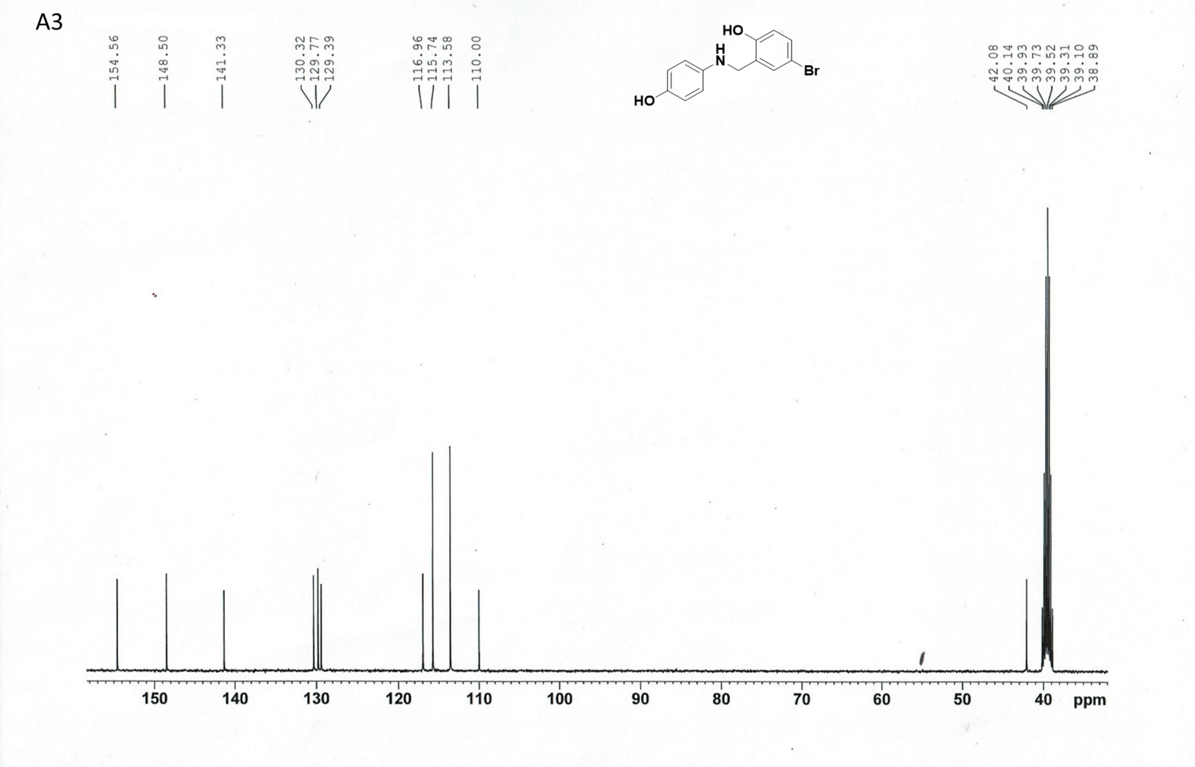

3-((5-Bromo-2-hydroxybenzyl) amino) phenol (A4)

A4

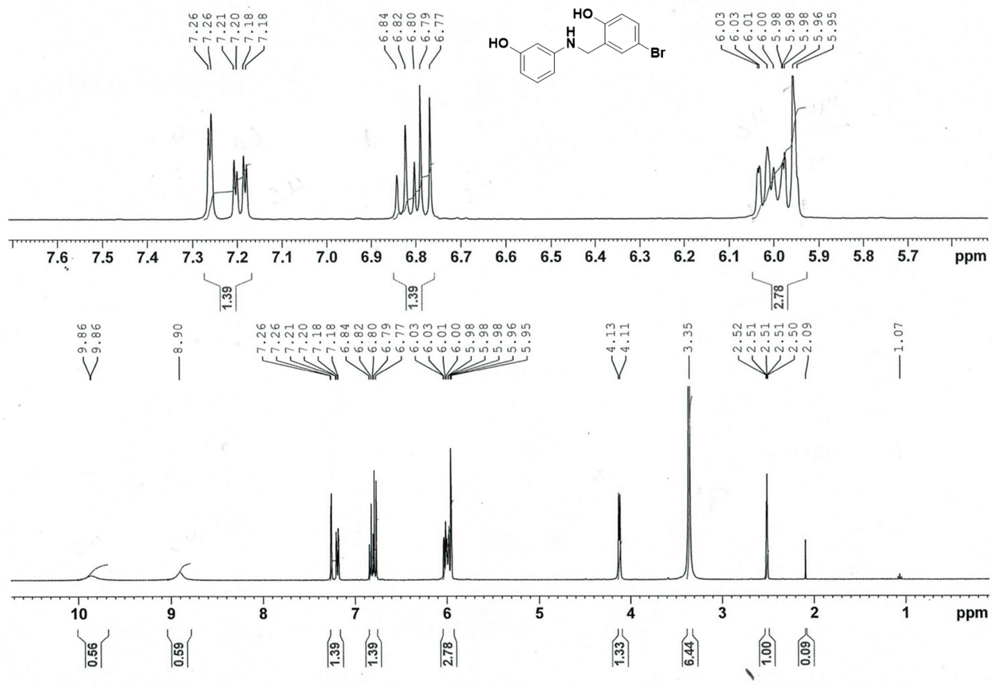

A4

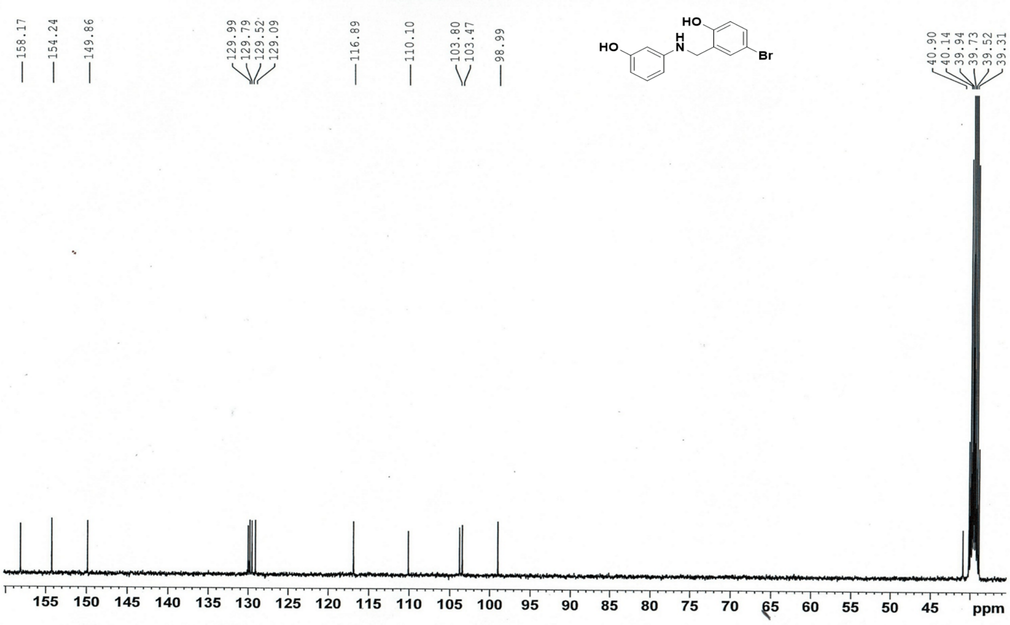

4-((5-Chloro-2-hydroxybenzyl) amino) phenol (A5)

A5

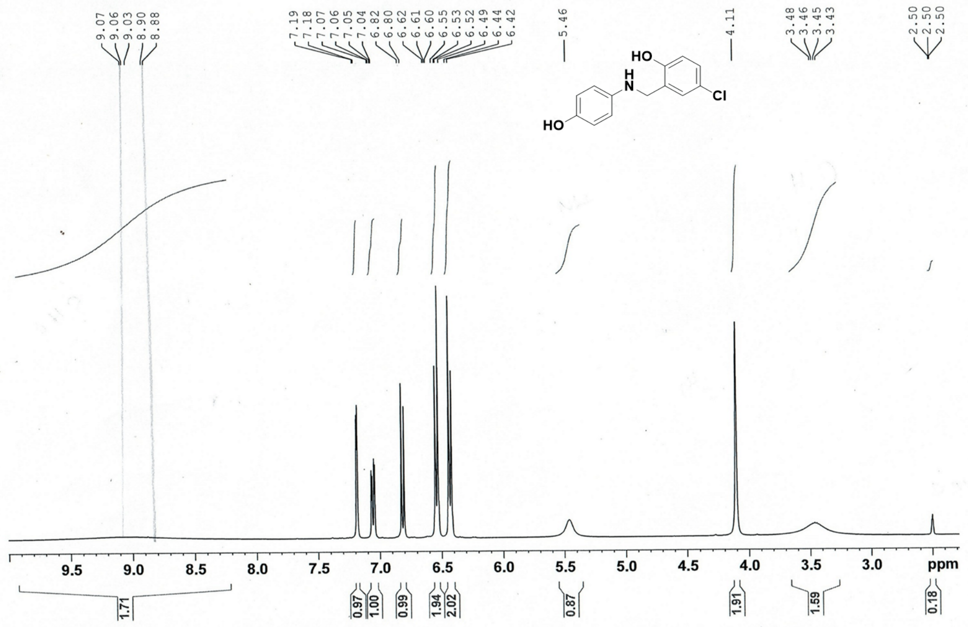

A5

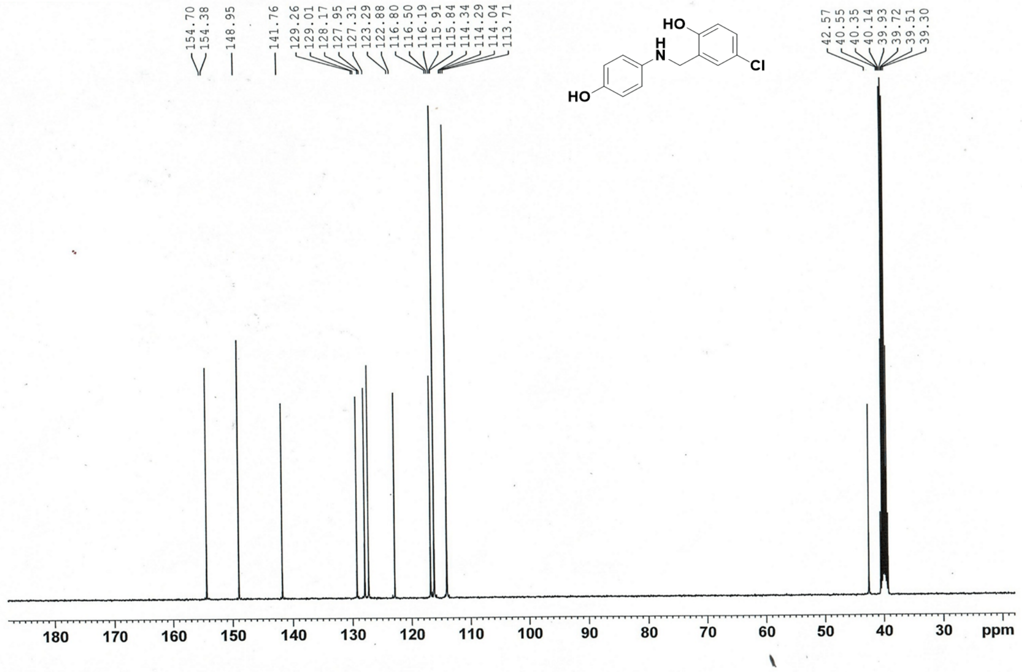

3-((5-Chloro-2-hydroxybenzyl) amino) phenol (A6)

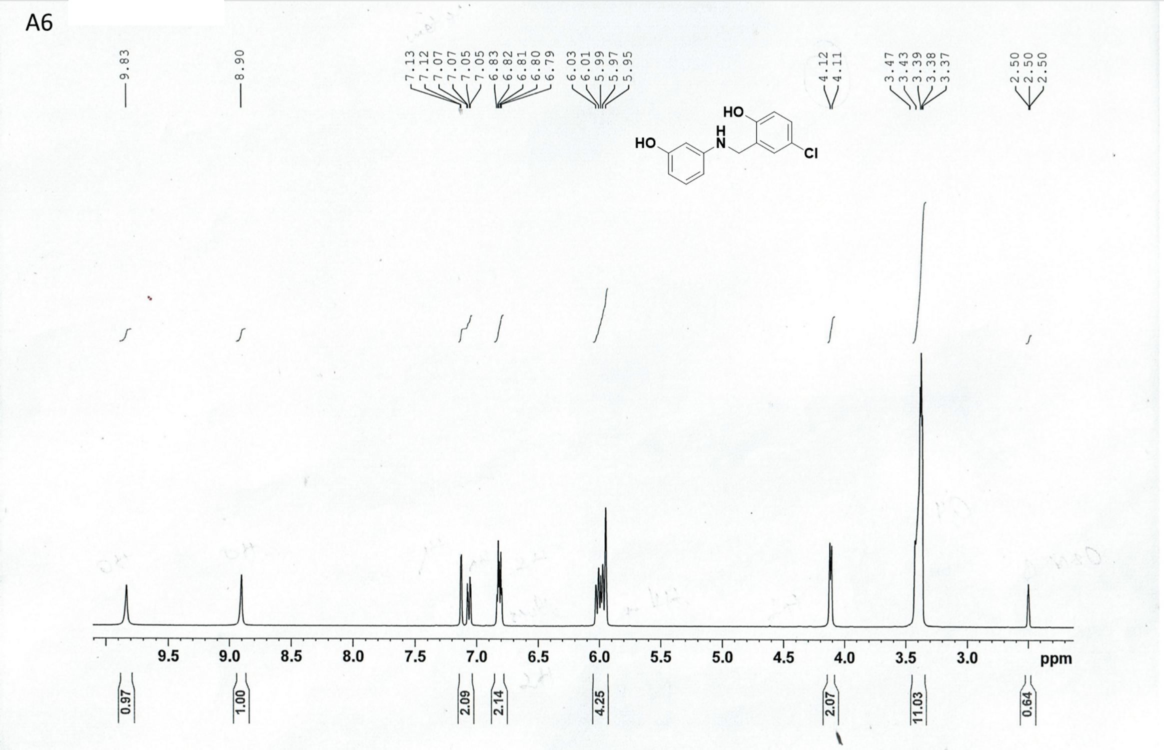

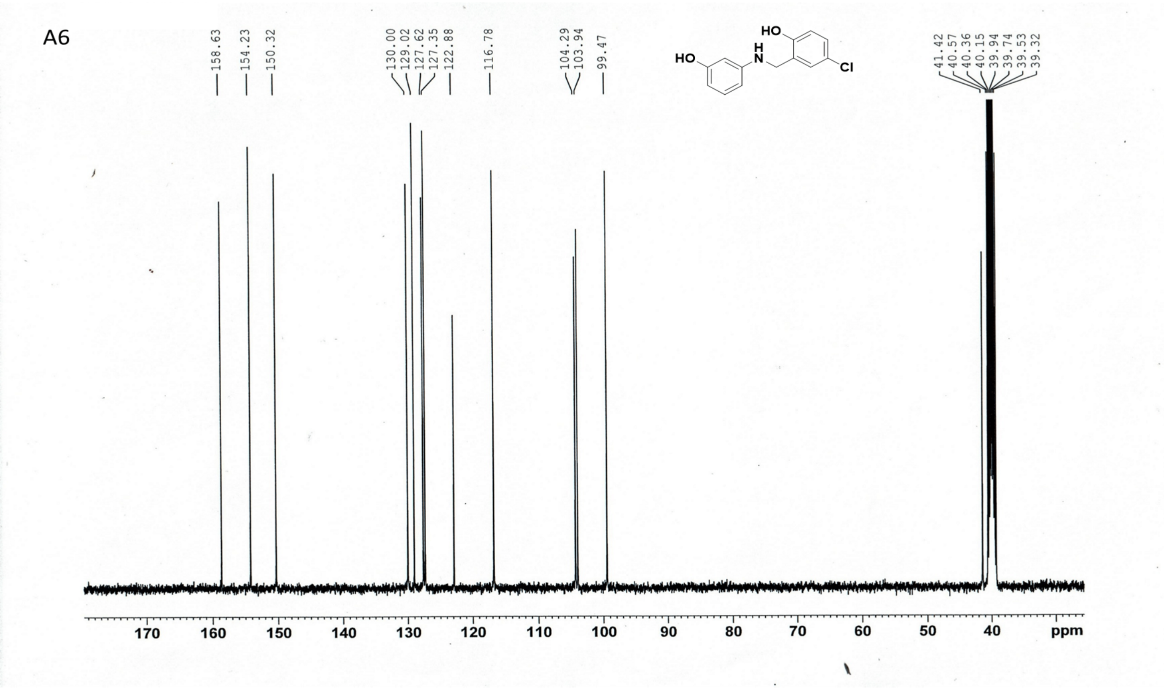

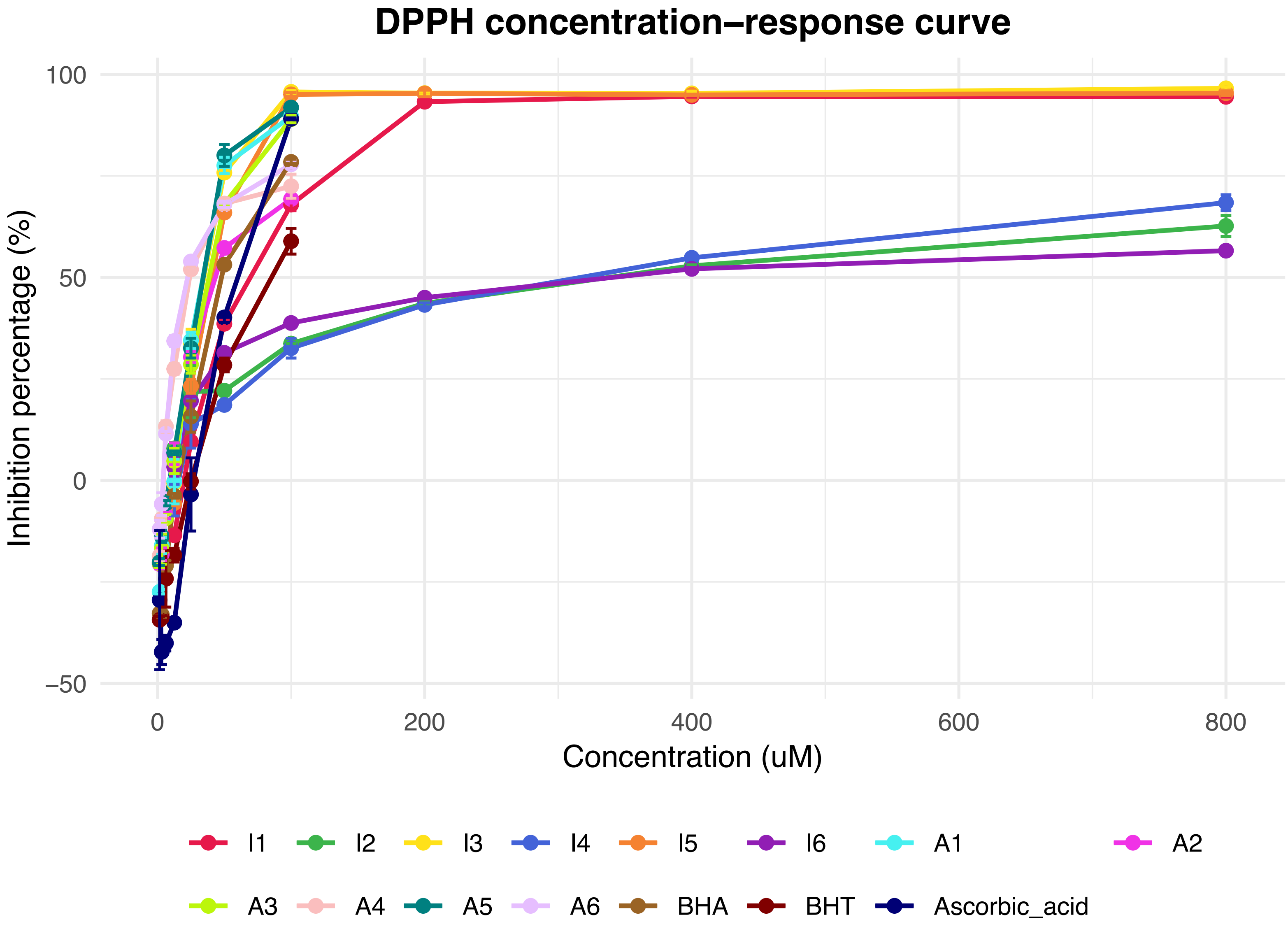
DPPH concentration−response curve
Inhibition percentage (%)
Concentration (uM)
100
50
0
−50
0
200
400
600
800
I1
I2
I3
I4
I5
I6
A1
A2
A3
A4
A5
A6
BHA
BHT
Ascorbic_acid

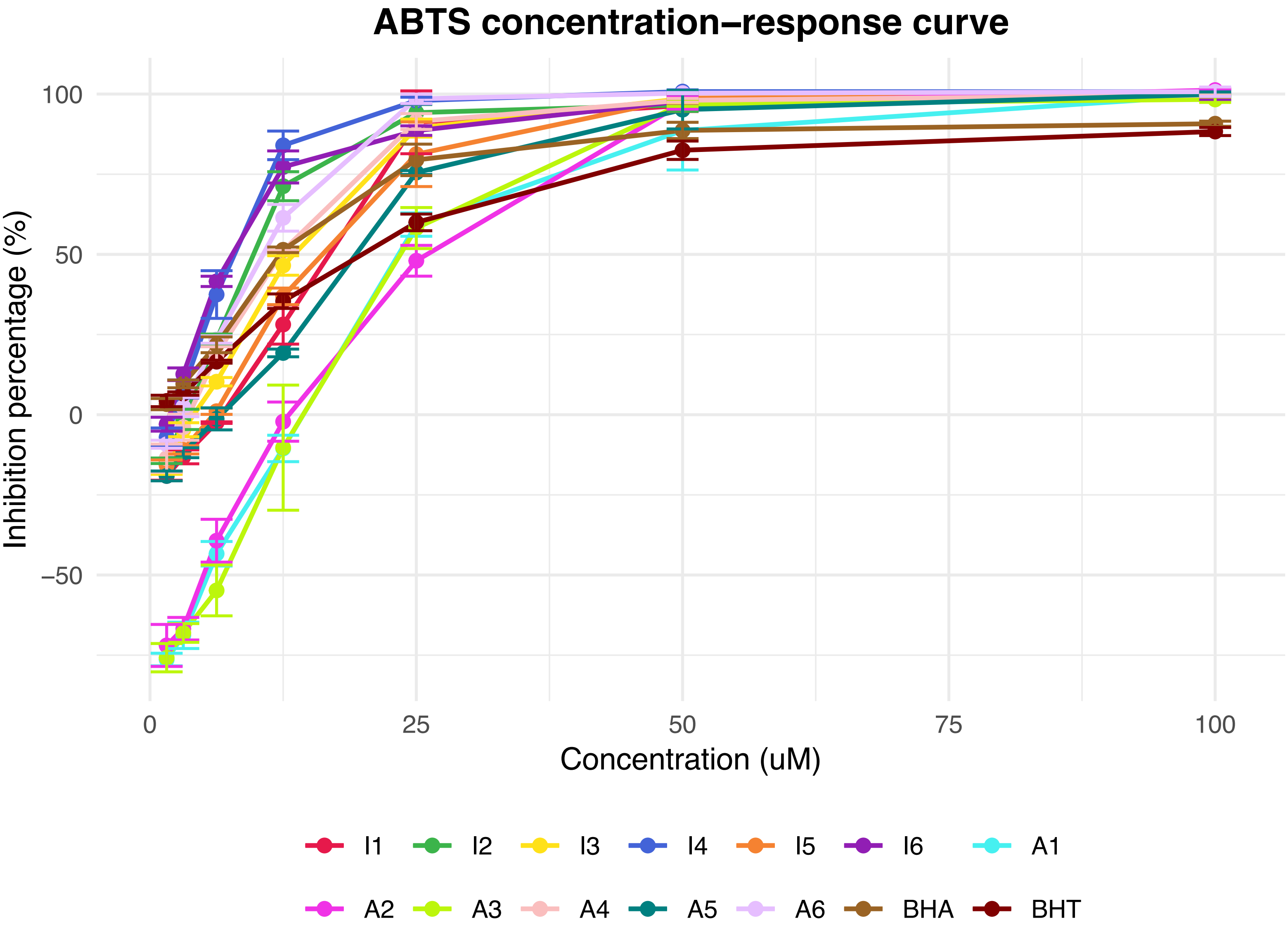
ABTS concentration–response curve
Inhibition percentage (%)
100
50
0
−50
0
25
50
75
100
Concentration (uM)
I1
I2
I3
I4
I5
I6
A1
A2
A3
A4
A5
A6
BHA
BHT

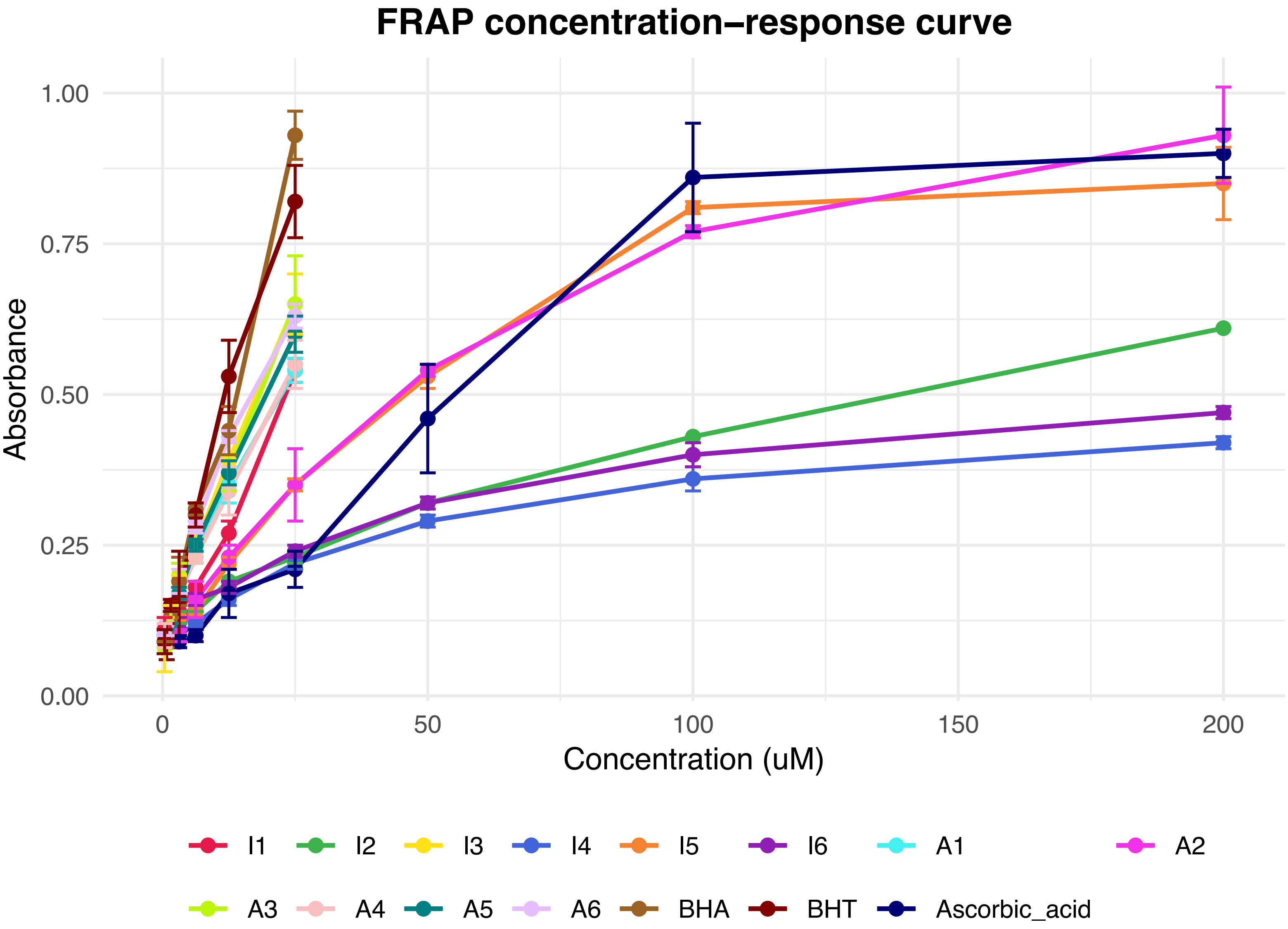
FRAP concentration−response curve
Absorbance
1.00
0.75
0.50
0.25
0.00
0
50
100
150
200
Concentration (uM)
I1
I2
I3
I4
I5
I6
A1
A2
A3
A4
A5
A6
BHA
BHT
Ascorbic_acid

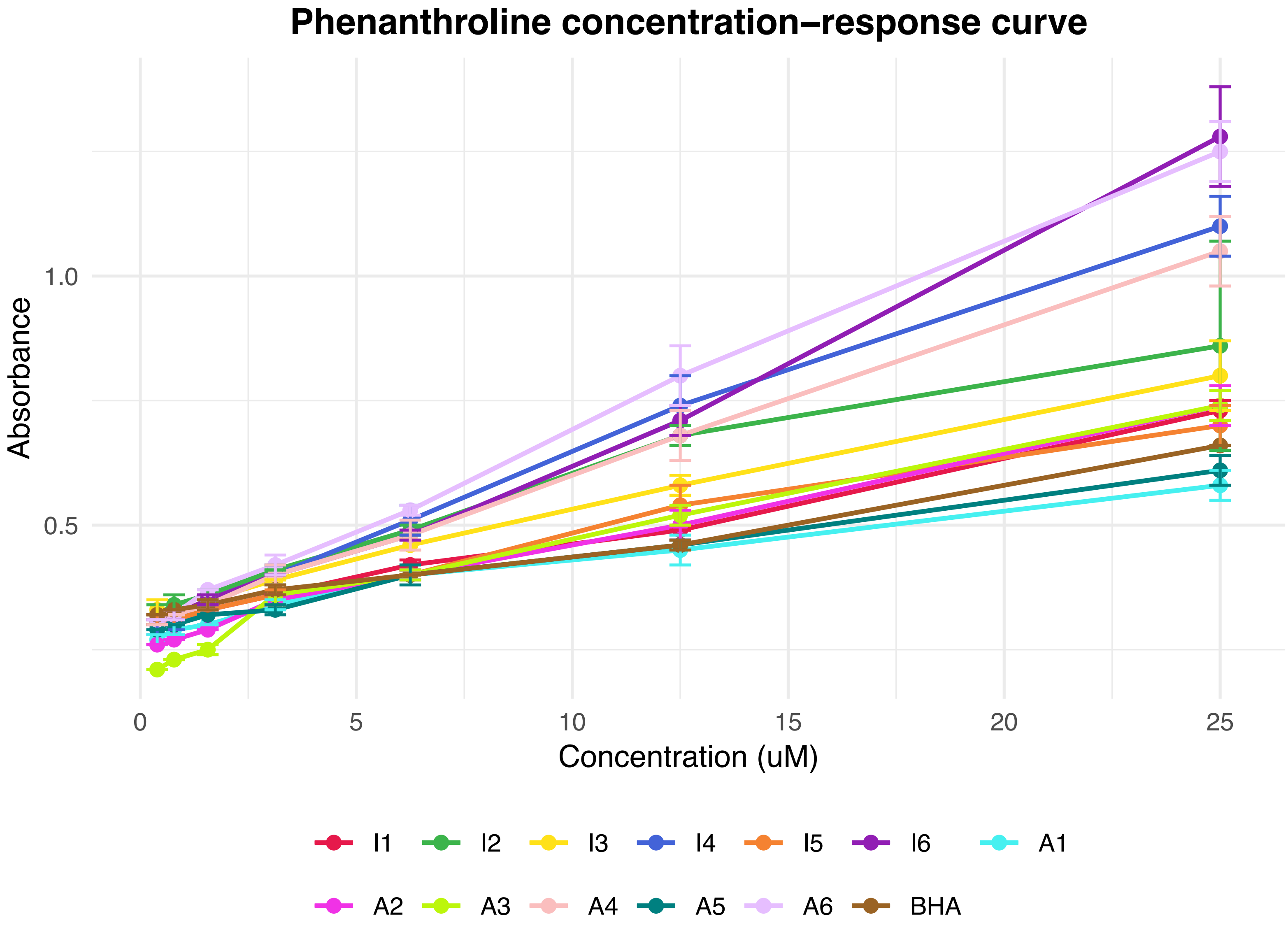

Phenanthroline concentration–response curve
Absorbance
1.0
0.5
0
5
10
15
20
25
Concentration (uM)
I1
I2
I3
I4
I5
I6
A1
A2
A3
A4
A5
A6
BHA

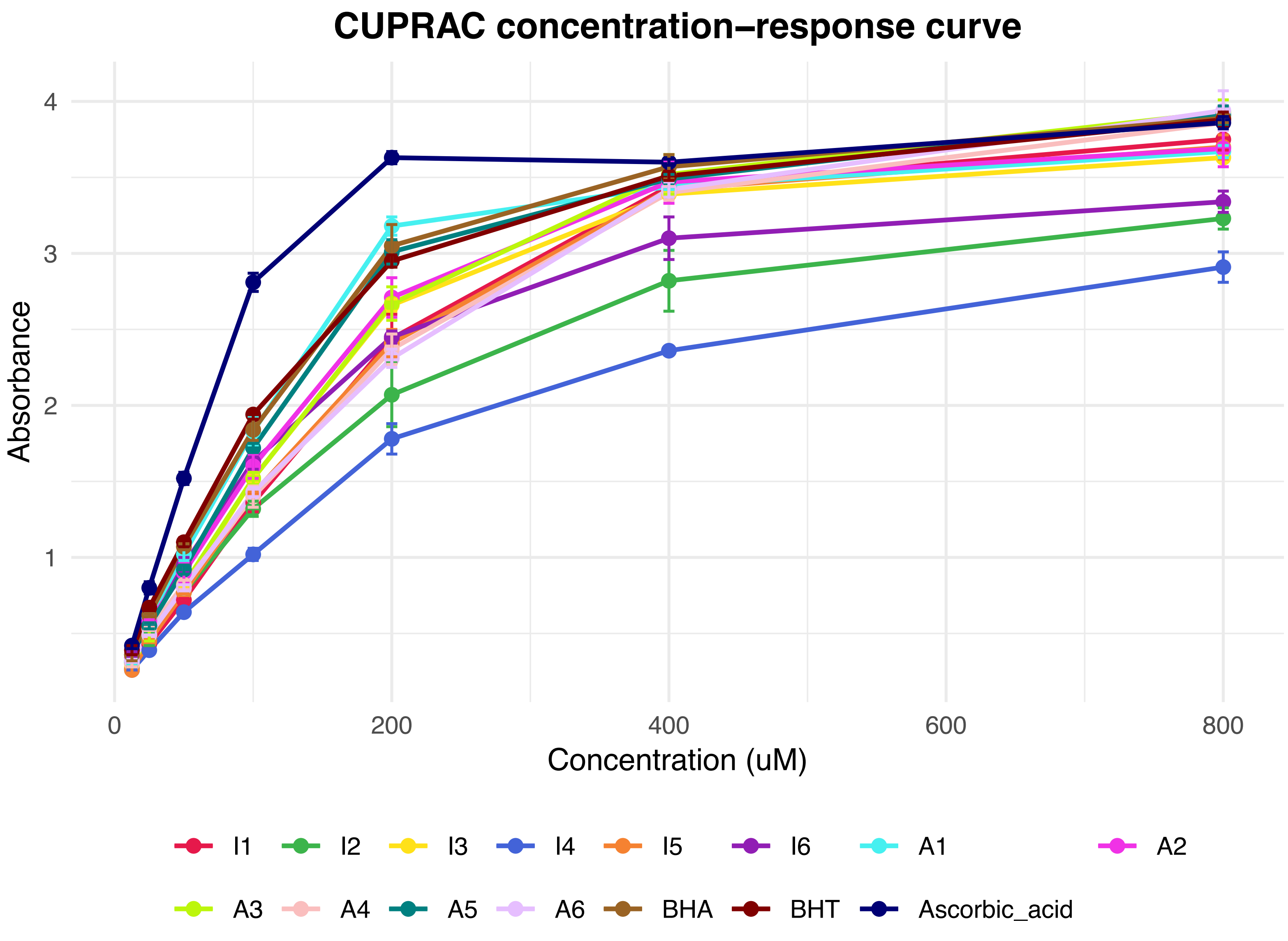
CUPRAC concentration–response curve
Absorbance
Concentration (uM)
0
200
400
600
800
1
2
3
4
I1
I2
I3
I4
I5
I6
A1
A2
A3
A4
A5
A6
BHA
BHT
Ascorbic_acid
